# Direct Synthesis of Allyl Amines with 2‐Nitrosulfonamide Derivatives via the Tsuji‐Trost Reaction

**DOI:** 10.1002/open.202100147

**Published:** 2021-08-15

**Authors:** Corentin Bon, Paola B. Arimondo, Ludovic Halby

**Affiliations:** ^1^ Epigenetic Chemical Biology Department of Structural Biology and Chemistry Institut Pasteur UMR3523 CNRS 75015 Paris France; ^2^ Ecole Doctorale MTCI Université de Paris Sorbonne Paris Cité Paris France

**Keywords:** allylation reactions, conjugated allyl amines, nosyl groups, palladium, Tsuji-Trost reactions

## Abstract

The Tsuji‐Trost Reaction is a palladium‐catalysed allylation of nucleophiles that consists in the reaction of a nitrogen, carbon or oxygen‐based nucleophiles with an allylic substrate bearing a leaving group. Here we present the use of 2‐nitrosulfonamide derivatives as nucleophile, which are reactive under mild conditions. 2‐nitrosulfonyl groups are well‐known dual protective activator groups easy to introduce in any type of amine substrates. The resulting 2‐nitrosulfonamide derivatives are ideal substrates for the Tsuji‐Trost reaction to afford a convenient and flexible access to primary and dissymmetric secondary allyl amines. The optimised procedure is flexible (for solvent, temperature, functional groups) and has been applied with good to excellent yield to access to a wide range of allyl amine derivatives.

## Graphical abstract

**A direct method** for the allylation of 2‐nitrosulfonamides is described and exemplified. The mild and flexible conditions used as well as the ease to remove the 2‐nitrosulfonamide group are compatible with a large set of chemical moieties.

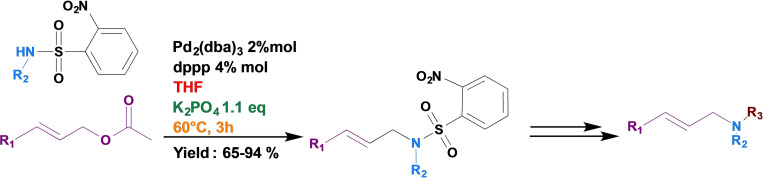

Allyl amine derivatives are recurrent moieties found as synthetic intermediates^[1]^ or bioactive compounds.[^2^, ^3^, ^4^] They are well‐known antifungal pharmacophores^[5]^ and are also commonly used in polymers for encapsulation and drug delivery (Figure 1).[^6^, ^7^, ^8^]

**Figure 1:**
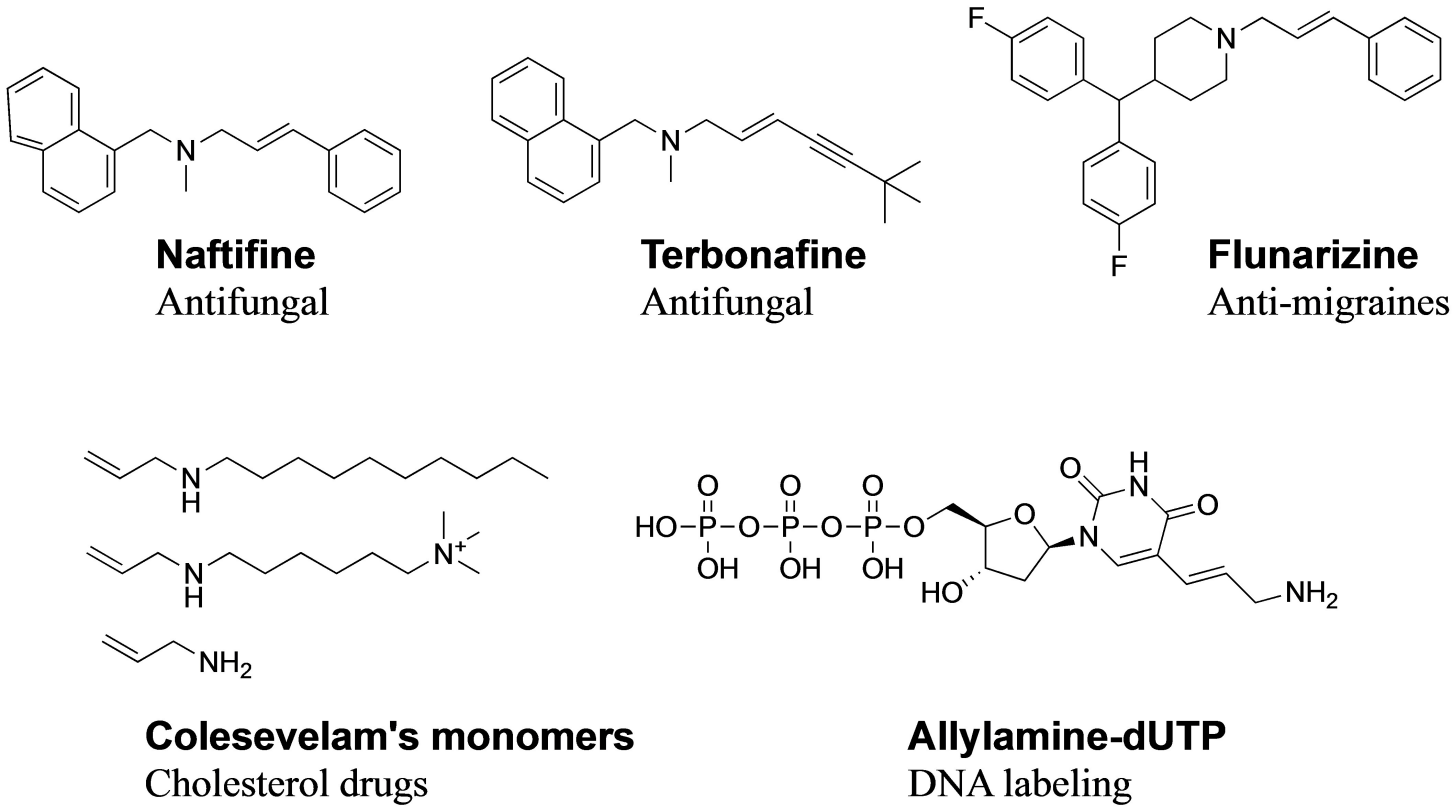
Examples of commercially used allylamine.[^9^, ^10^]

Traditionally, allylic amines are obtained by reactions of amines with reactive electrophiles^[11]^ involving hazardous reagents and often suffering from side reactions such as overalkylation, elimination, isomerization. Other methods requiring the preparation of specific reactants were developed such as imine alkenylation,^[12]^ using alkenyltrifluoroborates and *N*‐*tert*‐butanesulfinyl aldimines, or the Overman rearrangement^[13]^ of allylic trichloroacetimidates. Few example of metal‐catalysed allyl coupling are described in the literature; in addition, the diversity of products accessible is limited by the range of pKa and nucleophilicity of the corresponding moiety.[^14^, ^15^] Indeed, these nitrogen nucleophiles have to feature an appropriate electron density to allow the formation of transition metal intermediates. Amides and sulfonamides are the most common substitution,^[16]^ but such activating groups can then be difficult to remove. Also C−H amination‐based approaches feature similar amine activation limitations.[^17^, ^18^, ^19^] Cheng et al. reported a methodology using primary amines, however it is limited to alkyl amines and non‐conjugated alkenes.^[20]^ Moreover, such nitrogen‐carbon bond formations often lead to disubstitution on the amine.^[21]^ All these methods need to be optimised to allow an access to a larger diversity of allyl amine derivatives. Noteworthy, protected allyl amines are largely used to functionalize polyfunctional molecules through Upjohn dihydroxylation,^[22]^ hydroboration^[23]^ or Heck reaction^[24]^ thus there is a need to find an robust method to obtain them. An alternative option is to expand the Tsuji‐Trost reaction upon use 2‐nitrosulfonamide (nosyl) derivatives (Figure 2). In fact, this group is easily and selectively cleaved^[25]^ and leads to the corresponding primary or secondary amines in very good yield.^[26]^

**Figure 2:**
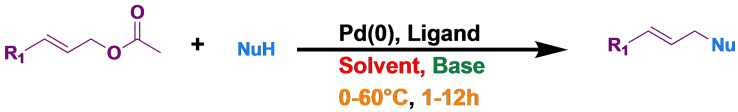
General conditions for the Tsuji‐Trost reaction.^[27]^

Sulfonamides have been studied in the past as substrate for Tsuji‐Trost coupling, especially tosylamides.^[28]^ However, the deprotection of the resulting tosyl‐bearing allyl amide requires harsh conditions such as boiling in concentrated sodium hydroxide.^[29]^ The use of the more labile 2‐nitrosulfonamide derivatives is less reported and only *N*‐(2‐nitrobenzenesulfonyl)*‐N‐*allyl amine derivatives have been described as efficient nucleophiles for Tsuji‐Trost coupling on cyclic electrophilic substrates.^[30]^ The reported method used complex and not commercially available ligands[^31^, ^32^, ^33^, ^34^, ^35^] or multistep addition of reagents leading to long reactions.[^33^, ^36^] Iridium complexes were also explored as catalyst, however only the 1,4‐addition products were obtained in good yields.^[37]^ To avoid such limitations, we optimized the conditions for the Tsuji‐Trost coupling reaction and obtained a method that allowed to obtain a wide range of allyl amine derivatives in good to excellent yields using commercially available ligands and stable palladium complex. In order to achieve this, we chose the 2‐nitrosulfonyl group (nosyl) as it is a dual protective and activating group efficiently cleavable in mild conditions and compatible with allylsulfonamide motifs.[^38^, ^39^] In addition, 2‐nitrosulfonamide derivatives are stable intermediates that are obtained upon 2‐nitrosulfonylchloride treatment of amines.

To define the most robust conditions for the reaction, unfavourable substrates were selected: the cinnamyl acetate **1 a**, as a deactivated electrophile, and 2‐nitro‐*N*‐phenylbenzenesulfonamide **2 a**, as a hindered electrophile. Based on the most common conditions for the Tsuji‐Trost reaction, catalyst, solvent, base, time and temperature were then optimized (Table 1).

**Table 1:**
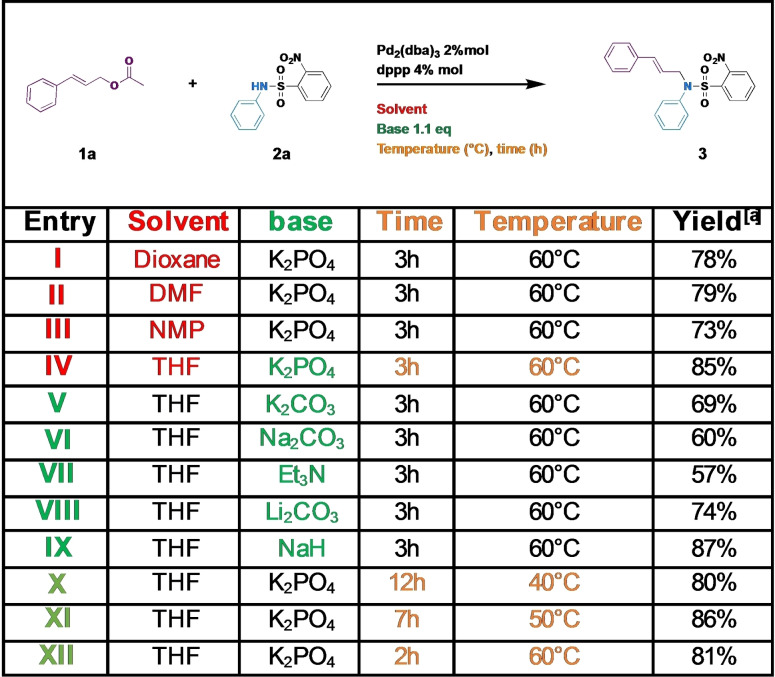
Optimization of the condition of reaction.

[a] 1 mmol scale synthesis, nucleophile (0.3 m), base (1.1 equiv). Yields are the average of three reactions.

Several common complexes of palladium were assessed but only tris(dibenzylideneacetone) dipalladium(0) [Pd_2_(dba)_3_] associated with 1,3‐Bis(diphenylphosphino)propane (dppp) allowed the reaction under normal atmosphere and was thus retained as catalyst.

The effect of the solvent was then evaluated (Table 1, entries I to IV): THF and dioxane were selected as aprotic low polar solvents and DMF and NMP as aprotic highly polar solvents. The polarity of the solvent had little effect and yields were comparable. This is a great advantage for a robust method, as it allows to apply the reaction to any substrates even those with poor solubility.

Next, six different bases were screened (Table 1, entries IV–IX). All bases afforded the desired product, with a lower efficiency for triethylamine and carbonates. Dibasic potassium phosphate and sodium hydride provided the best yields. However, we excluded sodium hydride as it was necessary to preliminary treat the nosyl derivative with sodium hydride in a dry solvent under inert atmosphere before addition to the electrophile substrate and the catalyst. Accordingly, dibasic potassium phosphate was selected as the most adequate base (Table 1, entry IV). The temperature and time were then optimised (Table 1, entries IV and X–XII) following the reaction by TLC until disappearance of the starting materials. As expected, the kinetics depended on the temperature. Interestingly, by varying the time of the reaction, the temperature can be adapted to the reactants and, for instance, take into account their stability and/or the need to control their isomerisation as illustrated below.

This optimisation led to a general procedure to obtain allyl amine derivatives based on the Tsuji‐Trost reaction using sulfonamide derivative and Pd_2_(dba)_3_ associated with dppp in the presence of dibasic potassium phosphate and adapting the solvent and temperature to the specificity of the reactants. The robustness of the procedure was demonstrated by the use of a wide range of electrophiles and nucleophiles (Table 2). In addition to cinnamyl acetate **1 a**, commercially available electrophiles (**1 b** to **1 d**) bearing aliphatic chain, conjugated diene and cyclic allyl acetate were selected to cover a broad range of functionalisation. The acetate group was used as leaving group because it can be conveniently added to alcohols either chemically or enzymatically.[^40^, ^41^] Similarly, seven sulfonamide derivatives (**2 a** to **2 g**) were selected to afford a large range of primary to secondary amines after denosylation. The corresponding nosyl derivatives were obtained by treatment of the desired amine with 2‐nitrosulfonyl chloride in pyridine and required no purification. The Tsuji‐Trost reaction tolerated a wide range of functionalised electrophiles and sulfonamide derivatives to afford polysubstituted allyl amines in good to excellent yields. No reaction was observed between the *N*‐Boc‐sulfonamide **2 b** and the cyclopentene derivative **1 d**, probably because of high steric hindrance. Lower yields were obtained using 2‐nitrosulfonamide **2 c** as nucleophile due to the occurrence of disubstituted side products, still yields remained above 60 %. Of note, the reaction with the soft nuclephiles sulfonamide derivatives^[42]^ proceeded with a double nucleophilic substitution, resulting in the conservation of the absolute configuration.[^43^, ^44^, ^45^] No isomerisation was observed except when using the diene (*E*,*E*)‐sorbyl acetate **1 c**. In this case, we observed the formation of 30 % of the (1*E*,3*Z*)‐pentadienyle **17** when the reaction was performed at 60 °C (80 % yield), 12 % when at 40 °C (78 % yield) and less than 5 % when at 0 °C (72 % yield) (Figure S1 in the Supporting Information). This underlines the advantage of the procedure, as by varying the temperature it is possible to control the isomerisation.

**Table 2:**
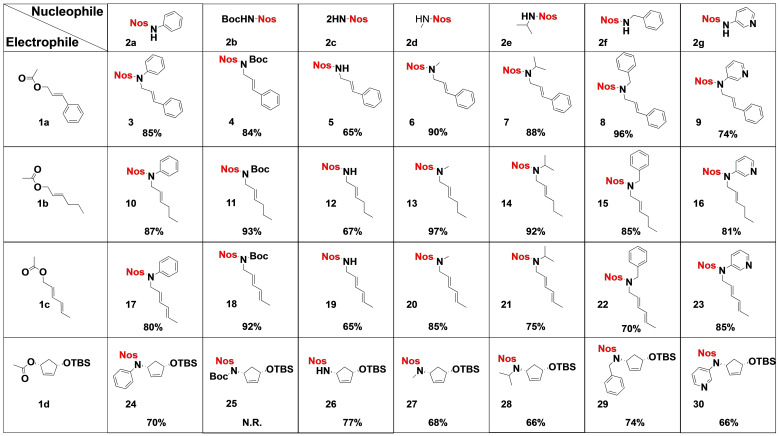
Application of the procedure: Reactants, products and yields.

As a general procedure, a solution of allyl acetate (1 mmol), Pd_2_(dba)_3_ (0.02 mmol) and dppp (0.04 mmol) in tetrahydrofuran (5 mL) was stirred at the indicated temperature for 30 min. Then, K_2_PO_4_ (1.1 mmol) and nosyl derivative (1 mmol) were added and the mixture was stirred at 60 °C for 3 h (TLC monitoring). The reaction mixture was filtered over celite, volatiles evaporated and the residue was purified by flash column chromatography with a linear 0–50 % gradient of ethyl acetate in cyclohexane.

In conclusion, we report here a robust, easy and flexible synthesis of primary and secondary allyl amine derivatives using the Tsuji‐Trost reaction. The use of 2‐nitrosulfonamide moiety instead of the usually employed toluene sulfonyl derivatives provide two advantages: 1) increased yields, due to an effective acidic component for the C−N bond formation, and 2) easily and selectively removable under mild conditions, in contrast to tosyl groups that are very difficult to cleave. Thus, the method profits from the dual properties of the nosyl group as a protective and activating agent. It proceeds under convenient conditions from easily synthetized or commercially available compounds. The yields were good to excellent using four different electrophiles and seven nosyl derivatives. The large variety of functional groups, solvent and temperature tolerance of the method allows an easy access to a wide range of allyl amine derivatives. The use of this approach to prepare more complex scaffolds is currently underway in order to reach biologically active compounds.

## Abbrevations

Boc: tert‐butoxycarbonyle
Dba: Dibenzylideneacetone
DMF: Dimethylformamide
Dppp: 1,3‐Bis(diphenylphosphino)propane
Et_3_N: triethylamine
THF: Tetrahydrofuran
NMP: *N*‐Methyl‐2‐pyrrolidone
Nos: Sulfonyl 2‐nitrobenzene
Nu: Nucleophile

## Conflict of interest

The authors declare no conflict of interest.

## Supporting information

As a service to our authors and readers, this journal provides supporting information supplied by the authors. Such materials are peer reviewed and may be re‐organized for online delivery, but are not copy‐edited or typeset. Technical support issues arising from supporting information (other than missing files) should be addressed to the authors.

Supporting Information
