# Supplementary material for: Direct Synthesis of Allyl Amines with 2‐Nitrosulfonamide Derivatives via the Tsuji‐Trost Reaction
Source: ChemistryOpen. 2021 Aug 15;10(12):1166–9. doi: 10.1002/open.202100147 (PMC8634766; doi:10.1002/open.202100147)

ChemistryOpen

Supporting Information

Direct Synthesis of Allyl Amines with 2-Nitrosulfonamide Derivatives via the Tsuji-Trost Reaction

Corentin Bon, Paola B. Arimondo,* and Ludovic Halby*

Contents

1. General information 1
2. Experimental Details and Characterization Data 1
   2.1. Sulphonamide derivatives 1
   2.2. Tert-butyl ((4-nitrophenyl)sulfonyl)carbamate (2b) 2
   2.3. Tsuji-Trost coupling 3
3. Analysis of compound 17's isomerisation depending on the temperature 13
4. NMR Spectra 14

1. General information

All chemicals were from Sigma-Aldrich, Alfa Aesar, Carbosynth and FluoChem. NMR expExperiments were recorded on an Agilent DirectDrive 500 spectrometer (Agilent Technologies, Santa Clara) with a proton resonating frequency of 499.8 MHz. Spectra were recorded using VnmrJ 4.2A (Agilent Technologies). Chemical shifts are given in ppm. Coupling constants *J* are measured in Hz. Splitting patterns are designed as follows: s, singlet; bs, broad singlet; d, doublet; bd broad doublet; t, triplet; brt, broad triplet; dd, doublet of a doublet; m, multiplet; ddd, doublet of a doublet of a doublet;ddt, doublet of doublet of a triplet;dq, doublet of quartet; q, quartet. MS-ESI were obtained on a Bruker MicroTOF. HRMS analyses were performed on a Q Exactive mass Spectrometer (Thermofisher) using direct injection. Samples were previously dissolved in a mix of water and acetonitrile (50/50) and 0.1% of formic acid. Full scans (150-2000Da) were acquired in positive ion mode with a resolution of 70,000. Isomerization was evaluated using reversed-phase HPLC system (Agilent 1200 series) equipped with a diode-array detector on a C18 reverse phase column (Kromasil, 5 µm, 100 Å; 4.6 × 150 mm) at a flow rate of 1 mL.min^{-1} using an isocratic elution with 58% acetonitrile in 10 mM triethylammonium acetate buffer over 20 min. FT-IR spectroscopy were performed with a Bruker Tensor 27 FT-IR-spectrophotometer between 500 and 4000 cm-1 with a resolution of 4 cm-1 and 50 scans per sample.

2. Experimental Details and Characterization Data

2.1. Sulphonamide derivatives

To a solution of 10.1 mmol of the desired amine in pyridine (50mL) at 0°C was added 10mmol of 2-nitrosulfonyl chloride by portion over 20min. The mixture was stirred at RT for 4h then volatiles were evaporated and the residual oil partitioned between ethyl acetate (150 mL) and 1M citric acid (50mL). The organic phase was washed with 1M citric acid, then with a solution of saturated $NaHCO_3$, brine and dried over Na_2SO_4. The organic phase was evaporated to yield nosyl amine derivatives as solids. Higher purity was achieved by recrystallisation in ethanol using approximatively 5mL/mmol.

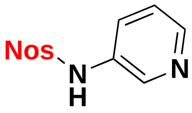

***N*-(pyridin-3-yl)-2-nitrobenzenesulfonamide (2g)**
2.4g, 8.1mmol, 80%, reddish solid.
^{1}H NMR (500 MHz, DMSO-*d*$_6$) δ 11.03 (s, 1H, NH), 8.34 – 8.26 (m, 2H), 8.01 – 7.95 (m, 2H), 7.88 – 7.76 (m, 2H), 7.51 (ddd, *J* = 8.3, 2.7, 1.5 Hz, 1H), 7.31 (ddd, *J* = 8.3, 4.7, 0.7 Hz, 1H).
^{13}C NMR (126 MHz, DMSO-*d*$_6$) δ 150.9, 145.2, 138.0, 135.8, 134.2, 133.0, 131.3, 127.8, 127.2.
HRMS-ESI(m/z) calculated for $C_{11}H_9N_3O_4S$ [M+H]$^+$: 280.0387; Found 280.0384

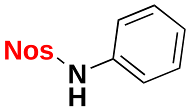

2-Nitro-*N*-phenylbenzenesulfonamide (2a)

2.6g, 9.0mmol, 90%, off-white solid.
^{1}H NMR (500 MHz, DMSO-*d*$_6$) δ 10.69 (s, 1H, NH), 7.97 – 7.91 (m, 2H), 7.84 – 7.73 (m, 2H), 7.28 – 7.21 (m, 2H), 7.14 – 7.08 (m, 2H), 7.11 – 7.01 (m, 1H).
^{13}C NMR (126 MHz, DMSO-*d*$_6$) δ 151.0, 139.7, 137.7, 135.6, 134.4, 133.0, 132.4, 127.7, 123.5.
HRMS-ESI(m/z) calculated for $C_{12}H_{10}N_2O_4S$ $[M+H]^+$: 279.0434; Found 279.0435.

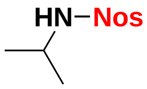

***N*-Isopropyl-2-nitrobenzenesulfonamide (2e)**
2.3g, 9.4mmol, 94%, white crystals.
^{1}H NMR (500 MHz, CDCl$_3$) δ 8.23 – 8.17 (m, 1H), 7.94 – 7.82 (m, 1H), 7.81 – 7.71 (m, 2H), 5.15 (d, *J* = 7.2 Hz, 1H), 3.69 (ddt, *J* = 13.1, 7.5, 6.6 Hz, 1H), 1.19 (s, 3H), 1.17 (s, 3H).
^{13}C NMR (126 MHz, CDCl$_3$) δ 135, 133.40, 132.8, 130.8, 125.4, 47.1, 23.6.
HRMS-ESI(m/z) calculated for $C_9H_{12}N_2O_4S$ $[M+H]^+$: 245.0591; Found: 245.0586.

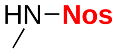

***N*-Methyl-2-nitrobenzenesulfonamide (2d)**
2.1g, 9.7mmol, 97%, white crystals.
^{1}H NMR (500 MHz, CDCl$_3$) δ 8.20-8.14 (d, 1H), 7.90-9.85 (d, 1H), 7.80-7.70 (m, 2H), 5.20 (bs, 1H), 2.80 (s, 3H).
^{13}C NMR (126 MHz, CDCl$_3$) δ 148.2, 133.7, 132.7, 132.4, 131.5 125.4,29.8**.**
HRMS-ESI(m/z) calculated for $C_7H_8N_2O_4S$ $[M+H]^+$: 217.0278; Found: 217.028.

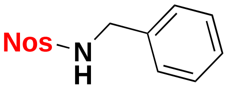

***N*-Benzyl-2-nitrobenzenesulfonamide (2f)**
2.8g, 9.5mmol, 95%, off white solid.
^{1}H NMR (500 MHz, DMSO-*d*$_6$) δ 8.62 (s, 1H), 7.90 (ddd, *J* = 15.4, 7.8, 1.4 Hz, 2H), 7.76 (ddt *J* = 25.2, 7.5, 1.4 Hz, 2H), 7.25 – 7.16 (m, 5H), 4.14 (s, 2H).
^{13}C NMR (126 MHz, DMSO-*d*$_6$) δ 150.6, 140.4, 137.0, 136.2, 135.6, 132.6, 131.3, 130.7, 130.3, 127.4, 49.3.
HRMS-ESI(m/z) calculated for $C_{13}H_{12}N_2O_4S$ $[M+H]^+$: 293.0591; Found 293.0587.

2.2. Tert-butyl ((4-nitrophenyl)sulfonyl)carbamate (2b)

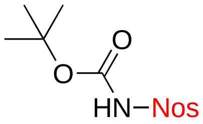

To a solution of 2-nitrosulfonylamide (7.28 g, 36.4 mmol) in CH_2Cl_2 (70 mL) was added Et_3N (7.7 mL, 60.9 mmol), DMAP (126mg mg, 1 mmol) and Boc_2O (9.5 g, 43.4 mmol). The reaction mixture was stirred at RT for 2h. The solution was diluted with 100 mL of 1M citric acid and the organic phase was washed with water, brine and dried over Na_2SO_4. The solvents were removed under vacuum to afford **2b** (10.6 g, 35mmol) as crystalline white powder.

^{1}H NMR (500 MHz, DMSO-*d*$_6$) δ 8.23 – 8.15 (m, 1H), 7.96 – 7.89 (m, 1H), 7.66 – 7.53 (m, 2H), 6.97 – 6.92 (m, 1H), 1.19 (s, 9H).
^{13}C NMR (126 MHz, DMSO-*d*$_6$) δ 159.8, 151.0, 143.0, 134.1, 133.7, 125.8, 110.0, 48.8, 31.2.
HRMS-ESI(m/z) calculated for $C_{11}H_{14}N_2O_6S$ $[M+H]^+$: 303.0645; Found 303.0637.

2.3. Tsuji-Trost coupling

A solution of allyl acetate (1mmol), $Pd_2(dba)_3$ (0.02mmol) and dppp (0.04mmol) in 5mL of tetrahydrofuran was stirred at RT for 30min. Then K_2PO_4 (1.1mmol) and the nosyl derivative (1mmol) were added and the mixture stirred at 60°C for 3h (TLC monitoring). The crude was filtered on celite, volatiles evaporated and the residue was purified by flash column chromatography using a linear gradient 0-50 % ethyl acetate in cyclohexane.

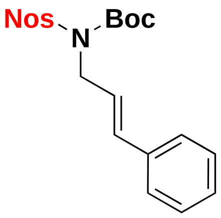

***tert*-Butyl cinnamyl((2-nitrophenyl)sulfonyl)carbamate (4)**
Experiment n°1: 0.8 mmol.
Experiment n° 2: 0.85 mmol.
Experiment n° 3: 0.87mmol.
Average Yield: 84%, clear oil.

^{1}H NMR (500 MHz, DMSO-*d*$_6$) δ 8.20 (dd, J = 7.9, 1.4 Hz, 1H), 8.09 (dd, J = 7.7, 1.5 Hz, 1H), 8.03 – 7.87 (m, 2H), 7.48 – 7.42 (m, 2H), 7.38 – 7.31 (m, 2H), 7.31 – 7.23 (m, 1H), 6.66 – 6.59 (m, 1H), 6.35 (dt, J = 15.9, 6.0 Hz, 1H), 4.45 (dd, J = 6.0, 1.4 Hz, 2H), 1.97 (s, 1H), 1.39 (d, J = 9.6 Hz, 2H), 1.25 (s, 9H).
^{13}C NMR (126 MHz, DMSO-*d*$_6$) δ 152.7, 150.2, 139.0, 138.7, 135.9, 134.8, 134.6, 131.8, 131.1, 129.6, 128.0, 127.4, 88.1, 52.0, 30.4.
HRMS-ESI(m/z) calculated for $C_{20}H_{22}N_2O_6S$ $[M+H]^+$: 419.1271; Found 419.1265.

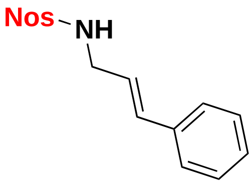

***N*-Cinnamyl-2-nitrobenzenesulfonamide (5)**
Experiment n° 1: 0.63 mmol.
Experiment n° 2: 0.60 mmol.
Experiment n° 3: 0.72 mmol.
Average Yield: 65%, clear oil.

^{1}H NMR (500 MHz, DMSO-*d*$_6$) δ 8.36 (s, 1H), 8.06 – 7.99 (m, 1H), 7.99 – 7.90 (m, 1H), 7.87 – 7.77 (m, 2H), 7.32 – 7.25 (m, 4H), 7.25 – 7.18 (m, 1H), 6.47 (dt, J = 15.9, 1.5 Hz, 1H), 6.09 (dt, J = 15.9, 6.0 Hz, 1H), 3.75 (dd, J = 6.0, 1.6 Hz, 2H), 1.16 (t, J = 7.1 Hz, 0H).
^{13}C NMR (126 MHz, DMSO-*d*$_6$) δ 150.8, 139.2, 137.A, 136.2, 135.7, 134.6, 132.8, 131.7, 130.8, 129.4, 129.3, 128.3, 127.4.
HRMS-ESI(m/z) calculated for $C_{15}H_{14}N_2O_4S$ $[M+Na]^+$: 341.0566; Found 341.0566.
IR (cm^{-1}): 1541 (NO_2), 1361 (NO_2), 1337 (SO), 1229 (NH), 1057 (SO).

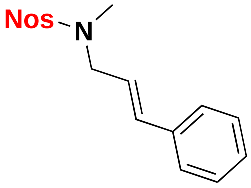

***N*-Cinnamyl-*N*-methyl-2-nitrobenzenesulfonamide (6)**
90%, clear oil.

^{1}H NMR (500 MHz, DMSO-*d*$_6$) δ 8.07 – 8.02 (m, 1H), 7.99 (dd, *J* = 7.8, 1.4 Hz, 1H), 7.87 (m, 2H), 7.42 – 7.37 (m, 2H), 7.40 – 7.28 (m, 2H), 7.28 – 7.21 (m, 1H), 6.59 (dd, *J* = 15.9, 1.5 Hz, 1H), 6.17 (dt, *J* = 15.9, 6.5 Hz, 1H), 3.99 (dd, *J* = 6.6, 1.4 Hz, 2H), 2.83 (s, 3H).
^{13}C NMR (126 MHz, DMSO-*d*$_6$) δ 150.9, 139.1, 137.7, 136.8, 135.5, 133.6, 133.1, 131.8, 131.7, 131.1, 129.6, 129.5, 127.4, 126.6, 54.7, 37.3, 29.5.
HRMS-ESI(m/z) calculated for $C_{16}H_{16}N_2O_4S$ [M+Na]$^+$: 355.0723; found 355.0719.
IR (cm^{-1}): 1540 (NO_2), 1540 (C=C), 1491 (C=C), 1373 (NO_2) 1350 (SO),1059 (SO).

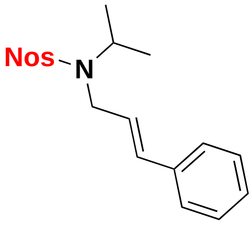

***N*-Cinnamyl-*N*-isopropyl-2-nitrobenzenesulfonamide (7)**

Experiment n° 1: 0.89 mmol.
Experiment n° 2: 0.86 mmol.
Experiment n° 3: 0.89 mmol.
Average Yield: 88%, yellow oil.
^{1}H NMR (500 MHz, DMSO-*d*$_6$) δ 8.06 (dd, *J* = 7.8, 1.5 Hz, 1H), 7.94 (dd, *J* = 7.8, 1.4 Hz, 1H), 7.81 (ddt, *J* = 23.9, 7.6, 1.4 Hz, 2H), 7.38 – 7.32 (m, 2H), 7.34 – 7.27 (m, 2H), 7.27 – 7.20 (m, 1H), 6.61 – 6.53 (m, 1H), 6.16 (dt, *J* = 15.9, 6.4 Hz, 1H), 4.14 – 4.03 (m, 3H), 1.12 (d, *J* = 6.7 Hz, 6H).
^{13}C NMR (126 MHz, DMSO-*d*$_6$) δ 150.6, 139.3, 137.5, 135.7, 135.5, 135.0, 133.3, 131.8, 130.8, 130.4, 129.3, 127.3, 53.02, 47.7, 24.0.
HRMS-ESI(m/z) calculated for $C_{18}H_{20}N_2O_4S$ [M+Na]$^+$: 383.1036; found 383.1027.
IR (cm^{-1}): 1540 (NO_2), 1370 (NO_2), 1338 (SO), 1058 (SO).

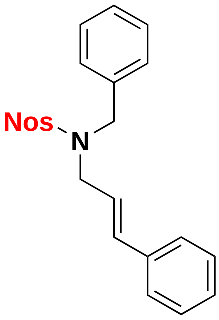

***N*-Benzyl-*N*-cinnamyl-2-nitrobenzenesulfonamide (8)**
Experiment n° 1: 0.94 mmol.
Experiment n° 2: 0.98 mmol.
Experiment n° 3: 0.96 mmol.
Average Yield: 96%, yellow oil.
^{1}H NMR (500 MHz, DMSO-*d*$_6$) δ 8.11 (dd, *J* = 7.9, 1.4 Hz, 1H), 8.00 (dd, *J* = 7.9, 1.3 Hz, 1H), 7.87 (td, *J* = 7.7, 1.4 Hz, 1H), 7.81 (td, *J* = 7.7, 1.3 Hz, 1H), 7.37 – 7.17 (m, 11H), 6.36 (d, *J* = 16.0 Hz, 1H), 5.94 (dt, *J* = 15.9, 6.6 Hz, 1H), 4.53 (s, 2H), 3.99 (dd, *J* = 6.6, 1.4 Hz, 2H).

^{13}C NMR (126 MHz, DMSO-*d*$_6$) δ 150.6, 139.1, 139.0, 137.8, 136.7, 135.6, 135.2, 133.3, 131.7, 131.6, 131.2, 131, 130.8, 129.4, 127.4, 126.3, 53.6, 52.1.
HRMS-ESI(m/z) calculated for $C_{22}H_{20}N_2O_4S$ [M+H]$^+$: 431.1036; found 431.1032.
IR (cm^{-1}): 1539(NO$_2$), 1438 (C=C), 1490 (C=C), 1353 (NO$_2$), 1341 (SO), 1065 (SO).

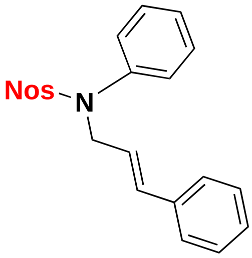

***N*-Cinnamyl-2-nitro-*N*-phenylbenzenesulfonamide (3)**
Experiment n° 1: 0.82 mmol.
Experiment n° 2: 0.85 mmol.
Experiment n° 3: 0.88 mmol.
Average Yield: 85%, clear oil.

^{1}H NMR (500 MHz, DMSO-*d*$_6$) δ 8.00 (dd, *J* = 8.0, 1.2 Hz, 1H), 7.90 (ddd, *J* = 8.0, 7.4, 1.4 Hz, 1H), 7.81 – 7.75 (m, 1H), 7.72 (dd, *J* = 8.0, 1.4 Hz, 1H), 7.40 – 7.17 (m, 11H), 6.50 – 6.43 (m, 1H), 6.20 (dt, *J* = 15.8, 6.5 Hz, 1H), 4.53 (dd, *J* = 6.5, 1.4 Hz, 2H).
^{13}C NMR (126 MHz, DMSO-*d*$_6$) δ 153, 142.9, 141.2, 140.2, 138.7, 137.5, 135.9, 135.7, 134.6, 134.4, 133.9, 133.7, 133.2, 131.7, 131.6, 129.6, 129.4, 58.8.
HRMS-ESI(m/z) calculated for $C_{21}H_{18}N_2O_4S$ [M+Na]$^+$: 417.0879; found 417.0871.

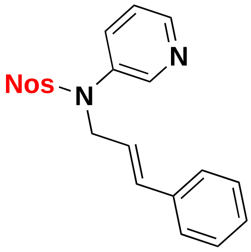

***N*-Cinnamyl-2-nitro-*N*-(pyridin-3-yl)benzenesulfonamide (9)**

Experiment n° 1: 0.70 mmol.
Experiment n° 2: 0.74 mmol.
Experiment n° 3: 0.78 mmol.
Average Yield: 74%, clear oil.
^{1}H NMR (500 MHz, DMSO-*d*$_6$) δ 8.50 (dd, *J* = 4.7, 1.5 Hz, 1H), 8.43 (dd, *J* = 2.6, 0.7 Hz, 1H), 7.99 (dd, *J* = 7.9, 1.1 Hz, 1H), 7.92 (ddd, *J* = 8.0, 6.8, 2.0 Hz, 1H), 7.88 – 7.77 (m, 2H), 7.70 (ddd, *J* = 8.2, 2.6, 1.5 Hz, 1H), 7.41 (ddd, *J* = 8.2, 4.7, 0.8 Hz, 1H), 7.35 – 7.30 (m, 2H), 7.30 – 7.23 (m, 2H), 7.27 – 7.17 (m, 1H), 6.51 – 6.43 (m, 1H), 6.19 (dt, *J* = 15.8, 6.6 Hz, 1H), 4.57 (dd, *J* = 6.5, 1.4 Hz, 2H).
^{13}C NMR (126 MHz, DMSO-*d*$_6$) δ 153.1, 152.2, 150.8, 139.8, 138.9, 138.4, 137.5, 137.2, 135.6, 133.7, 133.0, 131.73, 131.1, 129.5, 127.5, 127.2, 126.7, 56.4.
HRMS-ESI(m/z) calculated for $C_{20}H_{17}N_3O_4S$ [M+H]$^+$: 396.1013; found 396.1010.
IR (cm^{-1}): 1540(NO$_2$), 1475 (C=C), 1418 (C=C), 1394 (C=C) 1066 (SO), 1370(NO$_2$/SO).

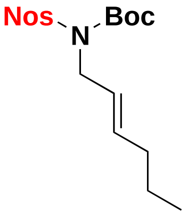

***Tert*-butyl (*E*)-hex-2-en-1-yl((2-nitrophenyl)sulfonyl)carbamate (11)**

Experiment n° 1: 0.92 mmol.
Experiment n° 2: 0.91 mmol.
Experiment n° 3: 0.95 mmol.

Average Yield: 93%, clear oil.

^{1}H NMR (500 MHz, DMSO-*d*$_6$) δ 8.16 – 8.10 (m, 1H), 8.10 – 8.02 (m, 1H), 8.02 – 7.85 (m, 2H), 5.69 (dtt, *J* = 14.9, 6.8, 1.3 Hz, 1H), 5.59 – 5.47 (m, 1H), 4.21 (dq, *J* = 6.0, 1.1 Hz, 2H), 1.99 (qd, *J* = 7.0, 1.3 Hz, 2H), 1.41 – 1.29 (m, 4H), 1.22 (s, 10H), 0.85 (dt, *J* = 11.5, 7.4 Hz, 3H).
^{13}C NMR (126 MHz, DMSO-*d*$_6$) δ 152.7, 138.5, 137.5, 135.7, 134.9, 134.4, 127.9, 127.9, 87.8, 51.6, 36.6, 30.3, 30.33, 24.8, 16.4.
HRMS-ESI(m/z) calculated for $C_{17}H_{24}N_2O_6S$ [M+H]$^+$: 385.1428 ; found 285.1422.

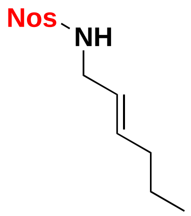

(*E*)-*N*-(Hex-2-en-1-yl)-2-nitrobenzenesulfonamide (12)
Experiment n° 1: 0.63 mmol.
Experiment n° 2: 0.69 mmol.
Experiment n° 3: 0.68 mmol.
Average Yield: 67%, clear oil.

^{1}H NMR (500 MHz, DMSO-*d*$_6$) δ δ 8.14 – 8.08 (m, 1H), 8.03 – 7.99 (m, 1H), 7.99 – 7.80 (m, 2H), 7.71 (s, 1H), 5.59 (dtt, *J* = 14.9, 6.8, 1.3 Hz, 1H), 5.59 – 5.47 (m, 1H), 4.21 (dq, *J* = 6.0, 1.1 Hz, 2H), 1.99 (qd, *J* = 7.0, 1.3 Hz, 2H), 1.41 – 1.29 (m, 4H), 0.91 (dt, *J* = 11.4, 7.2 Hz, 3H).
^{13}C NMR (126 MHz, DMSO-*d*$_6$) δ 150.3, 135.1, 134.8, 132.9, 129.2, 128.6, 124.2, 53.1, 35.6, 23.3, 15.5.
HRMS-ESI(m/z) calculated for $C_{12}H_{16}N_2O_4S$ [M+H]$^+$: 285.0904; Found 285.0900.

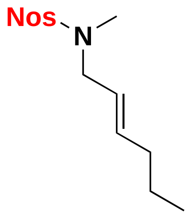

(*E*)-*N*-(Hex-2-en-1-yl)-*N*-methyl-2-nitrobenzenesulfonamide (13)

Experiment n° 1: 0.97 mmol.
Experiment n° 2: 0.97 mmol.
Experiment n° 3: 0.98 mmol.
Average Yield: 97%, clear oil.

^{1}H NMR (500 MHz, DMSO-*d*$_6$) δ 8.06 – 7.79 (m, 4H), 5.71 – 5.59 (m, 1H), 5.30 (m, 3.75 (dd, *J* = 6.6, 1.2 Hz, 2H), 2.75 (s, 3H), 1.98 – 1.91 (m, 2H), 1.30 (h, *J* = 7.4 Hz, 2H), 0.85 – 0.81 (m, 3H).
^{13}C NMR (126 MHz, DMSO-*d*$_6$) δ 150.8, 138.9, 137.6, 135.5, 133.7, 133.1, 127.3, 126.8, 54.5, 36.9, 36.7, 24.7, 16.5.
HRMS-ESI(m/z) calculated for $C_{13}H_{19}N_2O_4S$ [M+H]$^+$: 299.1060; found 299.1058.
IR (cm^{-1}): 1541(NO_2), 1375(NO_2), 1339 (SO), 1065 (SO).

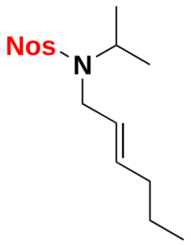

(*E*)-*N*-(Hex-2-en-1-yl)-*N*-isopropyl-2-nitrobenzenesulfonamide (14)
Experiment n° 1: 0.80 mmol.
Experiment n° 2: 0.86 mmol.
Experiment n° 3: 0.90 mmol.
Average Yield: 85%, clear oil.

^{1}H NMR (500 MHz, DMSO-d_6) δ 8.04 – 7.98 (m, 1H), 7.97 – 7.90 (m, 1H), 7.90 – 7.76 (m, 2H), 5.66 – 5.56 (m, 1H), 5.36 (dtt, *J* = 15.5, 6.4, 1.5 Hz, 1H), 3.83 (dq, *J* = 6.3, 1.1 Hz, 2H), 1.89 (qd, *J* = 7.0, 1.4 Hz, 2H), 1.27 (p, *J* = 7.3 Hz, 2H), 1.08 (s, 3H), 0.81 (t, *J* = 7.4 Hz, 3H).
^{13}C NMR (126 MHz, DMSO-d_6) δ 150.6, 137.4, 136.4, 135.9, 135.4, 133.2, 130.8, 127.2, 52.9, 47.5, 36.6, 24.7, 16.5.
HRMS-ESI(m/z) calculated for $C_{15}H_{22}N_2O_4S$ $[M+Na]^+$: 349.1192; found 349.1186.

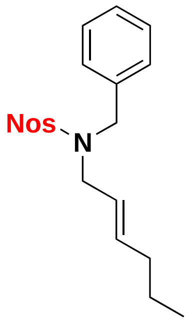

(*E*)-*N*-Benzyl-*N*-(hex-2-en-1-yl)-2-nitrobenzenesulfonamide (15)
Experiment n° 1: 0.88 mmol.
Experiment n° 2: 0.94 mmol.
Experiment n° 3: 0.93 mmol.
Average Yield: 92%, clear oil.

^{1}H NMR (500 MHz, DMSO-d_6) δ 8.13 – 7.78 (m, 4H), 7.41 – 7.18 (m, 5H), 5.49 – 5.38 (m, 1H), 5.12 (dtt, *J* = 14.9, 6.7, 1.5 Hz, 1H), 3.75 (d, *J* = 6.6 Hz, 2H), 1.85 – 1.76 (m, 2H), 1.17 (h, *J* = 7.3 Hz, 2H), 0.74 (t, *J* = 7.4 Hz, 3H).
^{13}C NMR (126 MHz, DMSO-d_6) δ 150.5, 139.1, 138.9, 137.7, 135.6, 135.3, 133.2, 131.6, 131.1, 130.7, 127.4, 126.3, 53.2, 51.8, 36.6, 24.5, 16.5.
HRMS-ESI(m/z) calculated for $C_{19}H_{22}N_2O_4S$ $[M+Na]^+$: 397.1192; found 397.1185.

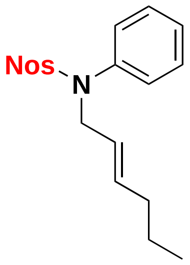

(*E*)-*N*-(Hex-2-en-1-yl)-2-nitro-*N*-phenylbenzenesulfonamide (10)
Experiment n° 1: 0.83 mmol.
Experiment n° 2: 0.91 mmol.
Experiment n° 3: 0.88 mmol.
Average Yield: 87%, yellow oil.

^{1}H NMR (500 MHz, DMSO-d_6) δ 7.98 – 7.94 (m, 1H), 7.90 – 7.84 (m, 1H), 7.77 – 7.73 (m, 1H), 7.67 (dd, *J* = 8.0, 1.3 Hz, 1H), 7.38 – 7.28 (m, 3H), 7.16 – 7.13 (m, 2H), 5.46 (dtt, *J* = 14.9, 6.8, 1.2 Hz, 1H), 5.36 – 5.27 (m, 1H), 4.27 (dq, *J* = 6.5, 1.0 Hz, 2H), 1.83 (qd, *J* = 7.0, 1.3 Hz, 2H), 1.16 (q, *J* = 7.3 Hz, 2H), 0.65 (t, *J* = 7.4 Hz, 2H).
^{13}C NMR (126 MHz, DMSO-d_6) δ 150.8, 140.6, 138.7, 138.0, 135.2, 133.6, 132.3, 132.2, 131.3, 127.4, 127.3, 56.3, 36.6, 24.6, 16.2.
HRMS-ESI(m/z) calculated for $C_{18}H_{20}N_2O_4S$ $[M+Na]^+$: 383.1036; found 383.1021.

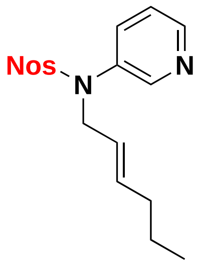

(*E*)-*N*-(Hex-2-en-1-yl)-2-nitro-*N*-(pyridin-3-yl)benzenesulfonamide (16)

Experiment n° 1: 0.75 mmol.
Experiment n° 2: 0.84 mmol.
Experiment n° 3: 0.83 mmol.
Average Yield: 81%, yellow oil.

^{1}H NMR (500 MHz, DMSO-d6) δ 8.51 (ddd, *J* = 7.3, 4.7, 1.5 Hz, 1H), 8.41 – 8.33 (m, 1H), 8.00 – 7.95 (m, 1H), 7.91 (ddd, *J* = 8.0, 5.6, 3.2 Hz, 1H), 7.87 – 7.77 (m, 2H), 7.64 (dddd, *J* = 26.9, 8.2, 2.6, 1.5 Hz, 1H), 7.45 – 7.38 (m, 1H), 5.48 (dtt, *J* = 14.9, 6.8, 1.1 Hz, 1H), 5.37 – 5.27 (m, 1H), 4.32 (dq, *J* = 6.6, 1.0 Hz, 2H), 1.82 (dddd, *J* = 12.6, 6.9, 5.9, 1.4 Hz, 2H), 1.15 (td, *J* = 7.2, 3.3 Hz, 2H), 0.64 (dt, *J* = 18.7, 7.4 Hz, 3H).
^{13}C NMR (126 MHz, DMSO-*d*$_6$) δ 153.1, 152.0, 150.7, 139.8, 139.6, 138.3, 138.3, 137.3, 135.5, 133.6, 133.6, 133.1, 127.4, 127.1, 127.0, 56.0, 36.5, 24.5, 16.1.
HRMS-ESI(m/z) calculated for $C_{17}H_{19}N_3O_4S$ [M+H]$^+$: 362.1169; found 362.1161.

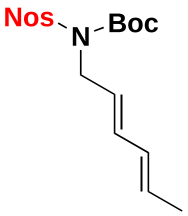

***Tert*-butyl ((2*E*,4*E*)-hexa-2,4-dien-1-yl)((2-nitrophenyl)sulfonyl)carbamate (18)**
Experiment n° 1: 0.88 mmol.
Experiment n° 2: 0.91 mmol.
Experiment n° 3: 0.94 mmol.
Average Yield: 92%, clear oil.

^{1}H NMR (500 MHz, DMSO-*d*$_6$) δ 8.21 – 7.85 (m, 4H), 6.26 – 5.98 (m, 2H), 5.77 – 5.58 (m, 2H), 4.27 (d, *J* = 6.3 Hz, 2H), 1.38 (d, *J* = 9.5 Hz, 3H), 1.22 (s, 9H).
^{13}C NMR (126 MHz, DMSO-*d*$_6$) δ 152.7, 150.2, 138.6, 136.8, 135.8, 134.9, 134.4, 133.6, 128.1, 128, 127.9, 87.9, 51.6, 30.4, 21.1.
HRMS-ESI(m/z) calculated for $C_{17}H_{22}N_2O_6S$ [M+Na]$^+$: 405.1091; found 405.1080.

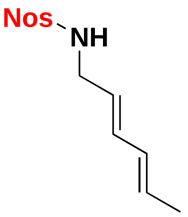

***N*-((2*E*,4*E*)-Hexa-2,4-dien-1-yl)-2-nitrobenzenesulfonamide (19)**
Experiment n° 1: 0.68 mmol.
Experiment n° 2: 0.62 mmol.
Experiment n° 3: 0.64 mmol.
Average Yield: 65%, clear oil.

^{1}H NMR (500 MHz, DMSO-*d*$_6$) δ 8.16 – 8.06 (m, 1H), 7.91 – 7.82 (m, 1H), 7.77 – 7.66 (m, 2H), 6.22 – 5.97 (m, 1H), 6.00 – 5.79 (m, 1H), 5.79 – 5.60 (m, 1H), 5.42 – 5.25 (m, 2H), 3.77 (td, *J* = 6.1, 5.5, 0.8 Hz, 1H), 1.79 – 1.61 (m, 3H).
^{13}C NMR (126 MHz, DMSO-*d*$_6$) δ 136.9, 136.1, 135.4, 133.9, 133.8, 132.6, 128.0, 126.3, 48.5, 20.7.
HRMS-ESI(m/z) calculated for $C_{12}H_{14}N_2O_4S$ [M+Na]$^+$: 305.0566; found 305.0562.

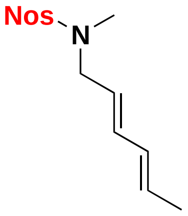

***N*-((2*E*,4*E*)-Hexa-2,4-dien-1-yl)-*N*-methyl-2-nitrobenzenesulfonamide (20)**
Experiment n° 1: 0.82 mmol.
Experiment n° 2: 0.87 mmol.

Experiment n° 3: 0.88 mmol.
Average Yield: 85%, clear oil.

^{1}H NMR (500 MHz, DMSO-*d*$_6$) δ 8.07 – 7.94 (m, 2H), 7.94 – 7.80 (m, 2H), 6.18 (td, *J* = 14.7, 14.2, 10.7 Hz, 1H), 6.12 – 5.94 (m, 1H), 5.86 – 5.65 (m, 1H), 5.59 – 5.38 (m, 1H), 2.80 – 2.71 (m, 3H), 1.81 – 1.63 (m, 3H).
^{13}C NMR (126 MHz, DMSO-*d*$_6$) δ 140.0, 139.9, 137.8, 137.7, 135.8, 135.8, 135.7, 135.3, 129.5, 129.3, 56.6, 39.3, 23.2.
HRMS-ESI(m/z) calculated for $C_{13}H_{16}N_2O_4S$ $[M+Na]^+$: 319.0723; found 319.0700.
IR (cm^{-1}): 2990 (C=C)**,** 1540 (NO_2), 1339 (NO_2), 1374 (SO), 1057 (SO).

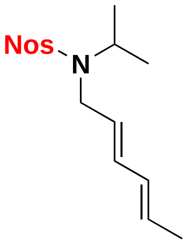

***N*-((2*E*,4*E*)-Hexa-2,4-dien-1-yl)-*N*-isopropyl-2-nitrobenzenesulfonamide (21)**
Experiment n° 1: 0.68 mmol.
Experiment n° 2: 0.80 mmol.
Experiment n° 3: 0.76 mmol.
Average Yield: 75%, yellow oil.

^{1}H NMR (500 MHz, DMSO-*d*$_6$) δ 8.02 (ddd, *J* = 7.8, 6.3, 1.6 Hz, 1H), 7.93 (dt, *J* = 7.8, 1.7 Hz, 1H), 7.91 – 7.75 (m, 2H), 6.15 (dd, *J* = 15.3, 10.5 Hz, 1H), 6.04 – 5.90 (m, 1H), 5.70 – 5.53 (m, 1H), 5.53 – 5.40 (m, 1H), 4.09 – 3.86 (m, 3H), 1.74 – 1.64 (m, 3H), 1.07 (dd, *J* = 7.9, 6.7 Hz, 6H).
^{13}C NMR (126 MHz, DMSO-*d*$_6$) δ 150.5, 137.6, 137.5, 135.7, 135.7, 135.4, 133.8, 133.6, 133.3, 133.2, 132.7, 131.2, 127.2, 52.9, 47.4, 29.5, 23.9, 21.0.
HRMS-ESI(m/z) calculated for $C_{15}H_{21}N_2O_4S$ $[M+H]^+$: 325.1217 ; found 325.1212.

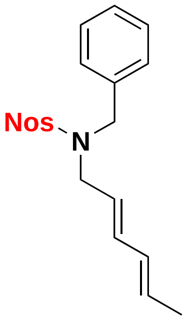

***N*-Benzyl-*N*-((2*E*,4*E*)-hexa-2,4-dien-1-yl)-2-nitrobenzenesulfonamide (22)**
Experiment n° 1: 0.68 mmol.
Experiment n° 2: 0.66 mmol.
Experiment n° 3: 0.76 mmol.
Average Yield: 70%, clear oil.

^{1}H NMR (500 MHz, DMSO-*d*$_6$) δ 8.09 – 8.04 (m, 1H), 8.02 – 7.97 (m, 1H), 7.92 – 7.79 (m, 2H), 7.36 – 7.20 (m, 6H), 6.01 – 5.84 (m, 2H), 5.65 – 5.56 (m, 1H), 5.27 – 5.18 (m, 1H), 4.44 (s, 2H), 3.82 (d, *J* = 6.8 Hz, 2H), 1.68 – 1.64 (m, 3H).
^{13}C NMR (126 MHz, DMSO-*d*$_6$) δ 150.5, 139, 137.8, 135.6, 135.2, 133.5, 133.4, 133.4, 133.23 133.2, 131.8, 131.7, 131.6, 131.2,130.8, 127.4, 126.6, 53.2, 20.9.
HRMS-ESI(m/z) calculated for $C_{19}H_{20}N_2O_4S$ $[M+H]^+$: 373.1217 ; found 373.1219.

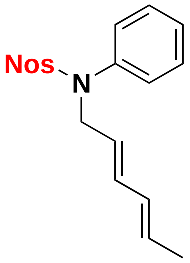

***N*-((2*E*,4*E*)-hexa-2,4-dien-1-yl)-2-nitro-*N*-phenylbenzenesulfonamide (17)**
Experiment n° 1: 0.71 mmol.
Experiment n° 2: 0.80 mmol.
Experiment n° 3: 0.87 mmol.
Average Yield: 80%, clear oil.

^{1}H NMR (500 MHz, DMSO-*d*$_6$) δ 7.96 (ddd, *J* = 8.0, 3.1, 1.2 Hz, 1H), 7.92 – 7.85 (m, 1H), 7.78 – 7.71 (m, 1H), 7.66 (ddd, *J* = 8.0, 4.3, 1.3 Hz, 1H), 7.40 – 7.29 (m, 4H), 7.21 – 7.13 (m, 2H), 6.08 – 5.85 (m, 2H), 5.65 – 5.52 (m, 1H), 5.49 – 5.39 (m, 1H), 4.34 (d, *J* = 6.6 Hz, 2H), 1.66 – 1.62 (m, 3H).
^{13}C NMR (126 MHz, DMSO-*d*$_6$) δ 150.7, 140.7, 138.1, 137.3, 135.2, 133.7, 133.6, 133.5, 133.4, 132.3, 132.1, 132.0, 129.9, 131.4, 131.3, 127.7, 56.3, 20.9.
HRMS-ESI(m/z) calculated for $C_{18}H_{18}N_2O_4S$ [M+Na]$^+$: 381.0879 ; found 381.0868.

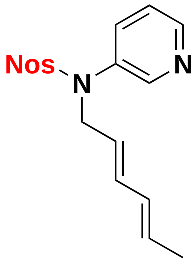

***N*-((2*E*,4*E*)-Hexa-2,4-dien-1-yl)-2-nitro-*N*-(pyridin-3-yl)benzenesulfonamide (23)**
Experiment n° 1: 0.86 mmol.
Experiment n° 2: 0.83 mmol.
Experiment n° 3: 0.84 mmol.
Average Yield: 85%, yellow wax.

^{1}H NMR (500 MHz, DMSO-*d*$_6$) δ 8.51 (ddd, *J* = 4.7, 2.4, 1.5 Hz, 1H), 8.37 (ddd, *J* = 10.7, 2.6, 0.8 Hz, 1H), 8.01 – 7.95 (m, 1H), 7.95 – 7.89 (m, 1H), 7.84 – 7.78 (m, 2H), 7.69 – 7.59 (m, 1H), 7.46 – 7.37 (m, 1H), 6.09 – 5.85 (m, 2H), 5.65 – 5.56 (m, 1H), 5.48 – 5.40 (m, 1H), 4.42 – 4.38 (m, 2H), 1.66 – 1.62 (m, 3H).
^{13}C NMR (126 MHz, DMSO-*d*$_6$) δ 153.0, 152.1, 139.6, 138.4, 138.0, 137.4, 135.5, 133.8, 133.6, 133.5, 133,0, 127.5, 127.19, 127.1, 56.0, 20.9.
HRMS-ESI(m/z) calculated for $C_{17}H_{17}N_3O_4S$ [M+H]$^+$: 360.1013 ; found 360.1000.

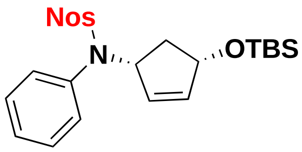

***N*-((1*R*,4*S*)-4-((*tert*-butyldimethylsilyl)oxy)cyclopent-2-en-1-yl)-2-nitro-*N*-phenylbenzenesulfonamide (24)**

Experiment n° 1: 0.64 mmol.
Experiment n° 2: 0.71 mmol.
Experiment n° 3: 0.75 mmol.
Average Yield: 70%, white solid.

^{1}H NMR (500 MHz, DMSO-*d*$_6$) δ 8.00 (dd, *J* = 8.0, 1.2 Hz, 1H), 7.89 (ddd, *J* = 8.0, 7.5, 1.4 Hz, 1H), 7.77 (ddd, *J* = 8.0, 7.4, 1.3 Hz, 1H), 7.70 (dd, *J* = 8.0, 1.3 Hz, 1H), 7.41 – 7.31 (m, 3H), 7.10 – 7.01 (m, 2H), 5.85 (ddd, *J* = 5.6, 2.0, 1.4 Hz, 1H), 5.71 (dt, *J* = 5.6, 2.0 Hz, 1H), 5.18 – 5.10 (m, 1H), 4.61 (ddq, *J* = 7.1, 5.2, 1.6 Hz, 1H), 2.68 (ddd,

J = 13.2, 8.0, 7.2 Hz, 1H), 1.38 (s, 4H), 0.71 (s, 8H), -0.09 (d, *J* = 8.7 Hz, 6H).
^{13}C NMR (126 MHz, DMSO-*d6*) δ 150.5, 140.8, 138, 136.9, 135.5, 135.4, 134.9, 134.6, 133.9, 132.2, 132.0, 127.5, 77.3, 66.0, 29.5, 28.8, 20.7, -1.7, -1.6.
HRMS-ESI(m/z) calculated for $C_{23}H_{30}N_2O_5SSi$ $[M+Na]^+$: 497.1536; found 497.1539

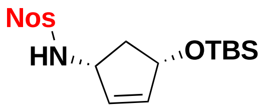

***N*-((1R,4S)-4-((*Tert*-butyldimethylsilyl)oxy)cyclopent-2-en-1-yl)-2-nitrobenzenesulfonamide (26)**
Experiment n° 1: 0.78 mmol.
Experiment n° 2: 0.73 mmol.
Experiment n° 3: 0.81 mmol.
Average Yield: 77%, yellow solid.

^{1}H NMR (500 MHz, DMSO-d_6) δ 8.43 (s, 1H), 8.06 – 7.99 (m, 1H), 7.98 – 7.91 (m, 1H), 7.90 – 7.82 (m, 2H), 5.78 (dt, *J* = 5.6, 2.0 Hz, 1H), 5.56 (dt, *J* = 5.6, 1.7 Hz, 1H), 4.65 – 4.59 (m, 1H), 4.12 (d, *J* = 8.4 Hz, 1H), 2.54 – 2.43 (m, 1H), 1.40 – 1.29 (m, 1H), 0.82 (s, 9H), 0.00 (s, 6H).
^{13}C NMR (126 MHz, DMSO-d_6) δ 150.7, 140.1, 137.2, 136.4, 135.6, 135.4, 132.8, 127.3, 77.9, 59.8, 45.0, 28.8, 20.8, -1.6.
HRMS-ESI(m/z) calculated for $C_{17}H_{26}N_2O_5SSi$ $[M+H]^+$: 399.1404; Found: 399.1399.

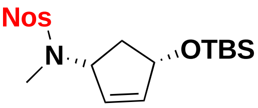

***N*-((1*R*,4*S*)-4-((*Tert*-butyldimethylsilyl)oxy)cyclopent-2-en-1-yl)-*N*-methyl-2-nitrobenzenesulfonamide (27)**

Experiment n° 1: 0.68 mmol.
Experiment n° 2: 0.65 mmol.
Experiment n° 3: 0.71 mmol.
Average Yield: 68%, clear oil.

^{1}H NMR (500 MHz, DMSO-d_6) δ8.08 – 8.03 (m, 1H), 8.00 – 7.94 (m, 1H), 7.93 – 7.81 (m, 2H), 5.96 (dt, *J* = 5.6, 2.0 Hz, 1H), 5.60 (ddd, *J* = 5.6, 2.3, 1.1 Hz, 1H), 4.84 – 4.78 (m, 1H), 4.65 (dddt, *J* = 7.4, 3.2, 2.0, 0.9 Hz, 1H), 2.66 (s, 3H), 2.44 (ddd, *J* = 14.3, 8.5, 7.4 Hz, 1H), 1.38 (s, 2H), 1.23 (ddd, *J* = 14.3, 4.3, 3.6 Hz, 1H), 0.81 (s, 9H), 0.01 (d, *J* = 9.9 Hz, 6H).
^{13}C NMR (126 MHz, DMSO-d_6) δ150.6, 141.5, 137.8, 135.5, 134.1, 133.9, 133.4, 127.3, 127.3, 77.5, 64.2, 40.1, 32.2, 29.5, 28.8, 28.8, 20.8, -1.6, -1.8.
HRMS-ESI(m/z) calculated for $C_{18}H_{28}N_2O_5SSi$ $[M+Na]^+$: 435.1380; found 435.1379.
IR (cm^{-1}): 1542 (NO_2), 1375 (NO_2/SO), 1075 (SO).

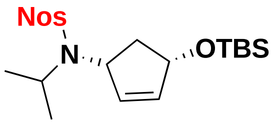

***N*-((1R,4S)-4-((*Tert*-butyldimethylsilyl)oxy)cyclopent-2-en-1-yl)-*N*-isopropyl-2-nitrobenzenesulfonamide (28)**
Experiment n° 1: 0.60 mmol.
Experiment n° 2: 0.73 mmol.
Experiment n° 3: 0. 63 mmol.
Average Yield: 66%, white solid.

^{1}H NMR (500 MHz, DMSO-*d6*) δ 8.00 – 7.94 (m, 1H), 7.94 – 7.88 (m, 1H), 7.88 – 7.79 (m, 2H), 5.93 (dt, *J* = 5.6, 2.1 Hz, 1H), 5.83 (ddd, *J* = 5.5, 2.2, 1.1 Hz, 1H), 4.69 – 4.62 (m, 1H), 4.62 – 4.55 (m, 1H), 3.73 (p, *J* = 6.9 Hz, 1H), 2.58 (ddd, *J* = 14.1, 8.5, 7.4 Hz, 1H), 1.55 – 1.47 (m, 1H), 1.26 – 1.19 (m, 6H), 0.82 (s, 9H).
^{13}C NMR (126 MHz, DMSO-*d6*) δ 150.9, 140.4, 137.4, 136.6, 135.6, 135.3, 132.5, 127.3, 77.5, 65.1, 52.0, 28.7, 25.6, 25.3, 20.7, -1.6, -1.7.
HRMS-ESI(m/z) calculated for $C_{20}H_{32}N_2O_5SSi$ $[M+Na]^+$: 463.1693; Found: 463.1688.

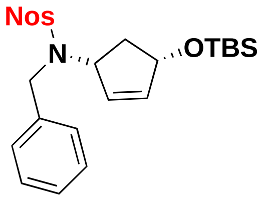

N-Benzyl-N-((1R,4S)-4-((tert-butyldimethylsilyl)oxy)cyclopent-2-en-1-yl)-2-nitrobenzenesulfonamide (29)
Experiment n° 1: 0.71 mmol.
Experiment n° 2: 0.69 mmol.
Experiment n° 3: 0.82 mmol.
Average Yield: 74%, white solide.

^{1}H NMR (500 MHz, DMSO-d_6) δ 8.05 (dd, *J* = 8.0, 1.3 Hz, 1H), 7.98 (dd, *J* = 8.0, 1.2 Hz, 1H), 7.89 (ddd, *J* = 8.0, 7.5, 1.3 Hz, 1H), 7.78 (ddd, *J* = 8.0, 7.5, 1.3 Hz, 1H), 7.31 – 7.16 (m, 6H), 5.83 (dt, *J* = 5.6, 2.1 Hz, 1H), 5.46 (ddd, *J* = 5.6, 2.3, 1.0 Hz, 1H), 4.90 (ddt, *J* = 8.4, 4.1, 2.1 Hz, 1H), 4.60 (dddd, *J* = 7.4, 3.1, 2.0, 0.9 Hz, 1H), 4.49 (d, *J* = 16.7 Hz, 1H), 4.39 (d, *J* = 16.7 Hz, 1H), 2.39 (ddd, *J* = 14.4, 8.5, 7.4 Hz, 1H), 1.19 (dt, *J* = 14.3, 3.6 Hz, 1H), 0.77 (s, 9H), -0.04 (d, *J* = 24.8 Hz, 6H).
^{13}C NMR (126 MHz, DMSO-*d6*) δ 150.6, 141.6, 141.2, 137.9, 135.5, 134.8, 134.4, 133.7, 131.3, 130.3, 130.1, 127.3, 77.38, 65.1, 49.9, 28.8, 20.7, -1.6, -1.8.
HRMS-ESI(m/z) calculated for $C_{24}H_{32}N_2O_5SSi$ [M+H]$^+$: 489.1874; found 489.1869.

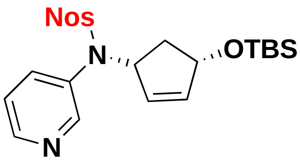

N-((1R,4S)-4-((Tert-butyldimethylsilyl)oxy)cyclopent-2-en-1-yl)-2-nitro-N-(pyridin-3-yl)benzenesulfonamide (30)
Experiment n° 1: 0.68 mmol.
Experiment n° 2: 0.71 mmol.
Experiment n° 3: 0.59 mmol.
Average Yield: 66%, yellow powder.

^{1}H NMR (500 MHz, DMSO-d_6) δ 8.54 (dd, *J* = 4.8, 1.5 Hz, 1H), 8.23 (dd, *J* = 2.5, 0.8 Hz, 1H), 8.04 – 7.96 (m, 1H), 7.92 (ddd, *J* = 8.0, 6.8, 2.0 Hz, 1H), 7.84 – 7.75 (m, 2H), 7.62 – 7.51 (m, 1H), 7.49 – 7.31 (m, 2H), 5.88 (ddd, *J* = 5.6, 2.1, 1.3 Hz, 1H), 5.82 – 5.68 (m, 2H), 5.24 – 5.16 (m, 1H), 4.62 – 4.52 (m, 1H), 2.73 (ddd, *J* = 13.8, 8.3, 7.3 Hz, 1H), 1.40 – 1.31 (m, 2H), 0.90 – 0.79 (m, 1H), 0.70 (s, 8H), -0.06 – -0.15 (m, 6H).
^{13}C NMR (126 MHz, DMSO-*d6*) δ 155.6, 152.8, 150.5, 143.2, 141.5, 138.3, 135.7, 134.5, 134.2, 133.9, 133.8, 132.1, 131.7, 127.7, 127.0, 77.2, 66.4, 28.8, 28.7, 20.7, -1.6, -1.7.
HRMS-ESI(m/z) calculated for $C_{23}H_{29}N_3O_5SSi$ [M+H]$^+$: 476.1670; found 476.1666.

3. Analysis of compound 17's isomerisation depending on the temperature

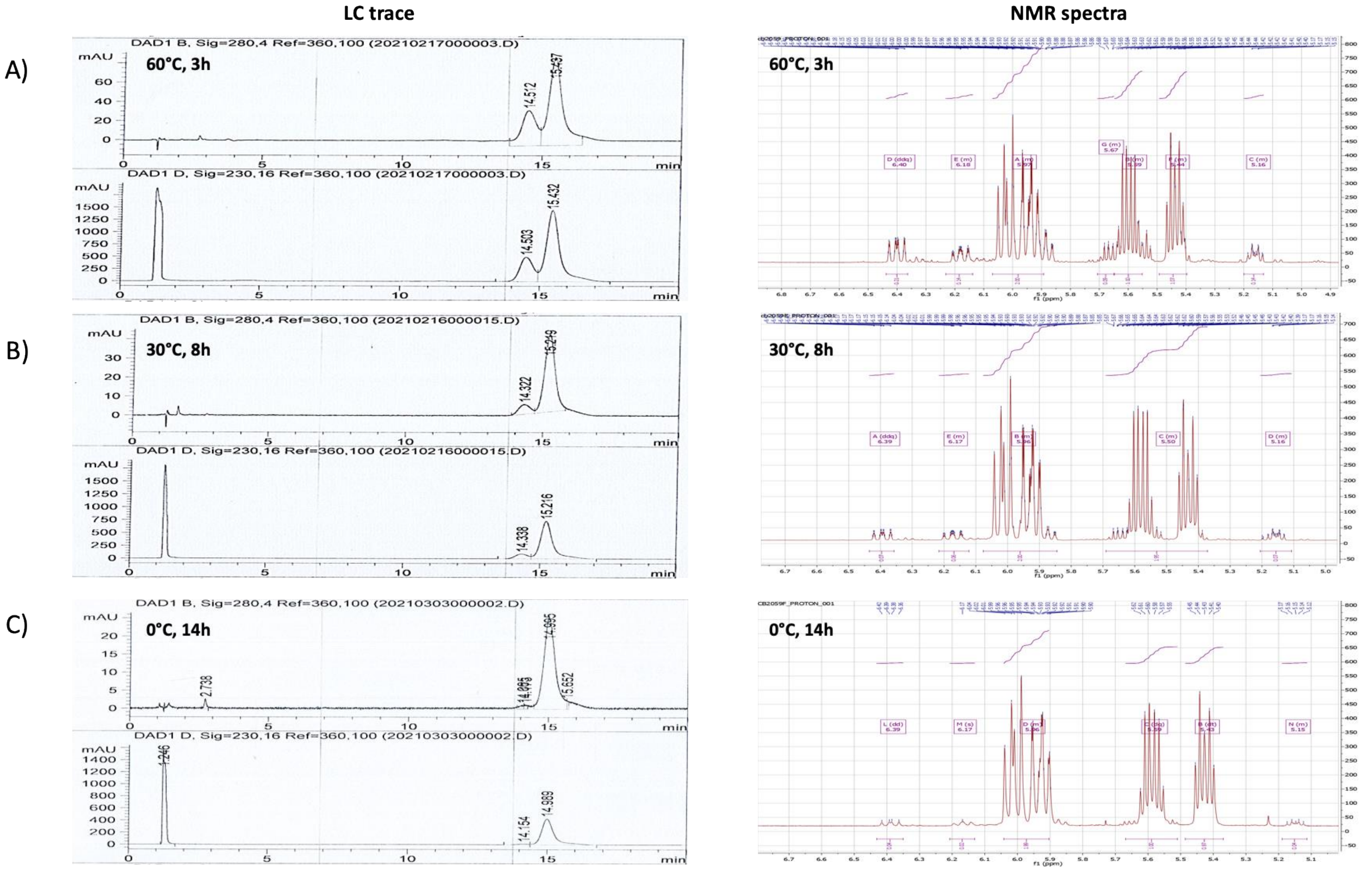

Supplementary Figure 1 Quantification of the isomerization in compound **17** synthesis by LC trace analysis using a linear gradient 58% acetonitrile in 10 mM triethylammonium acetate buffer over 20 min and by ^{1}H NMR. The reactions were carried out following the general procedure at A) 60°C for 3h, B) 30°C for 8h and C) at 0°C for 14h.

4. NMR Spectra

Spectrum compound 3

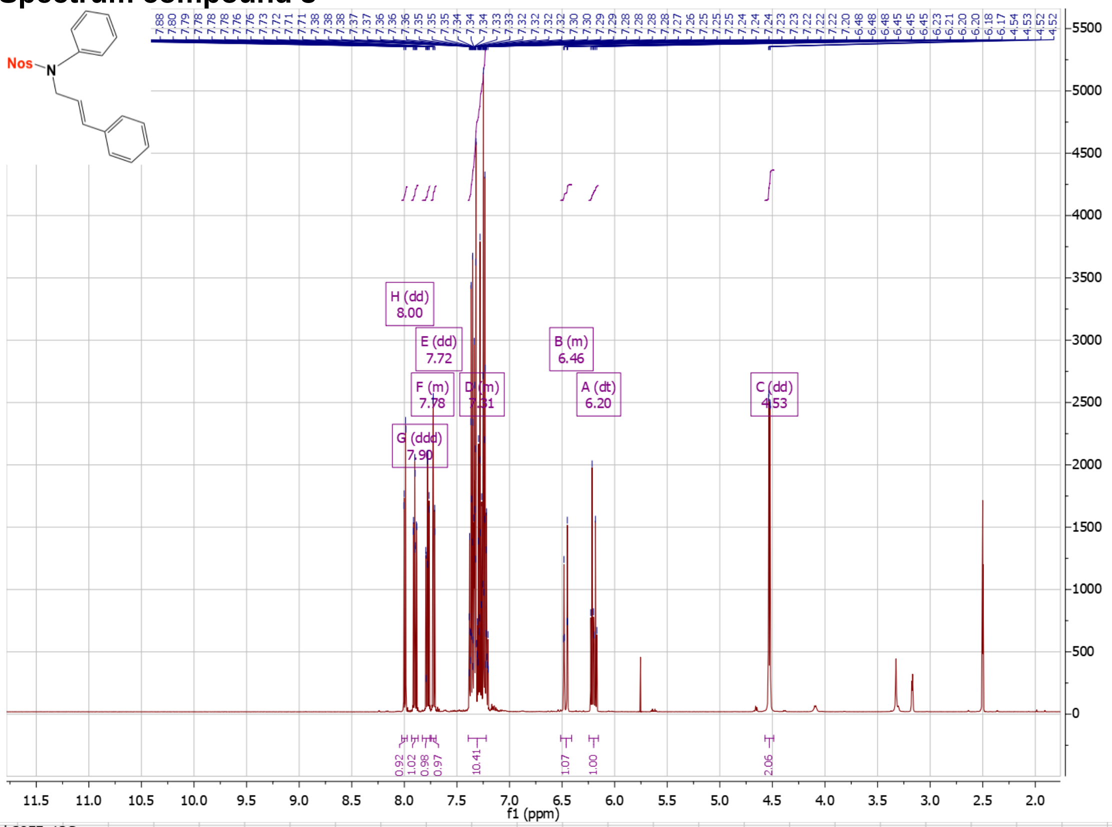

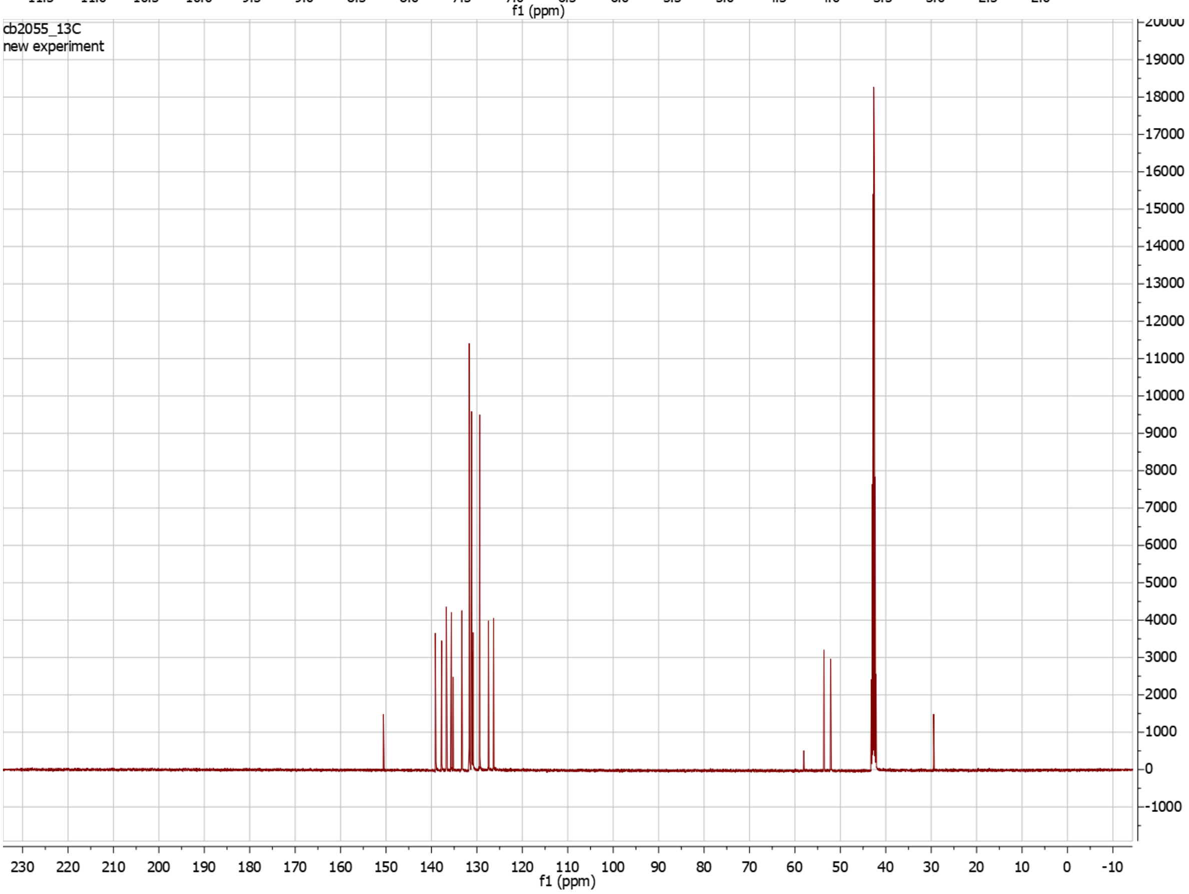

Spectrum Compound 4

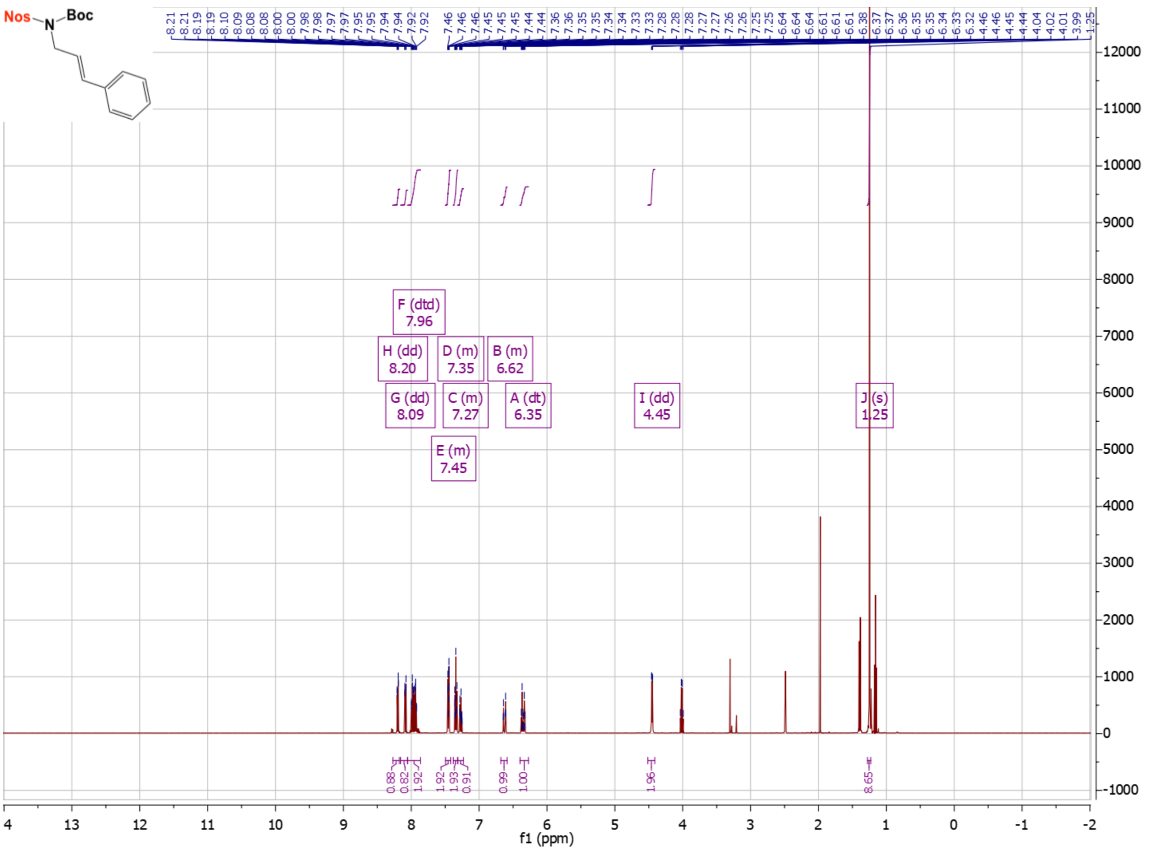

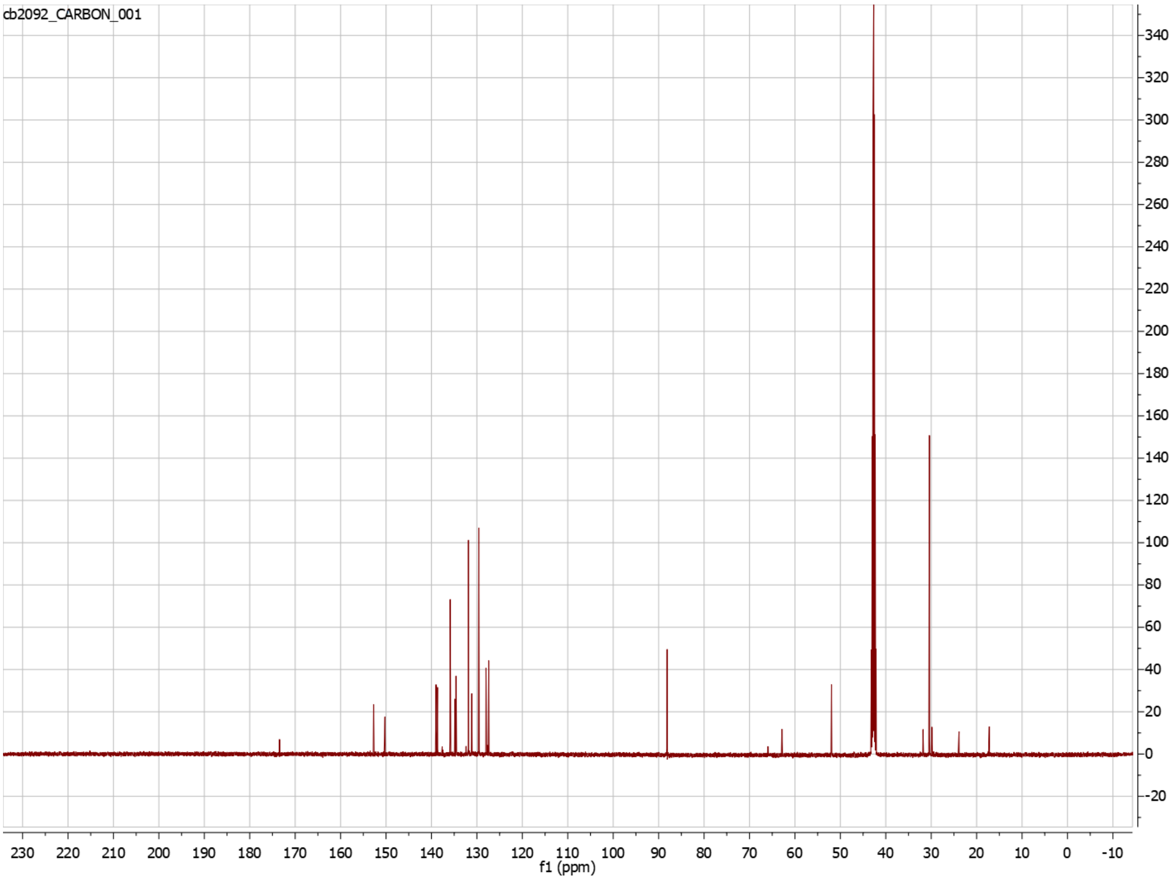

Spectrum compound 5

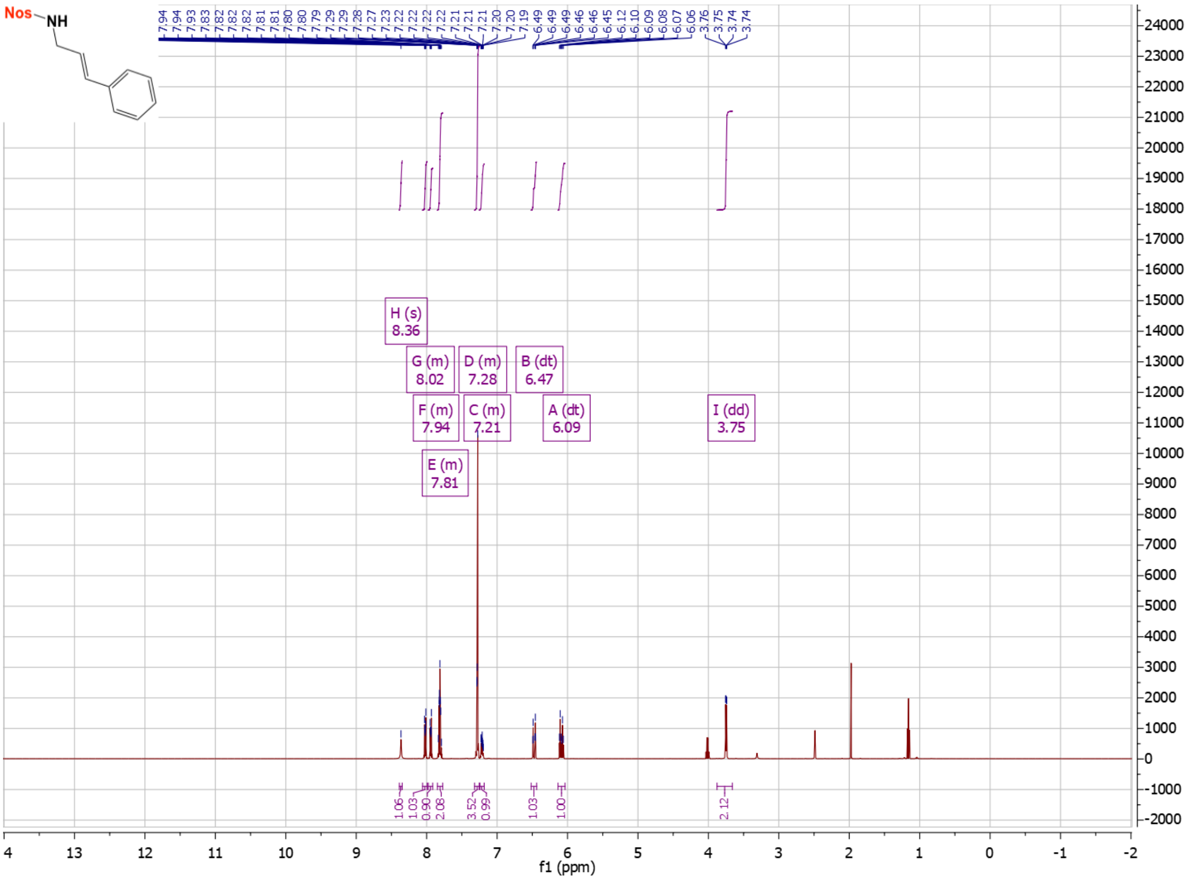

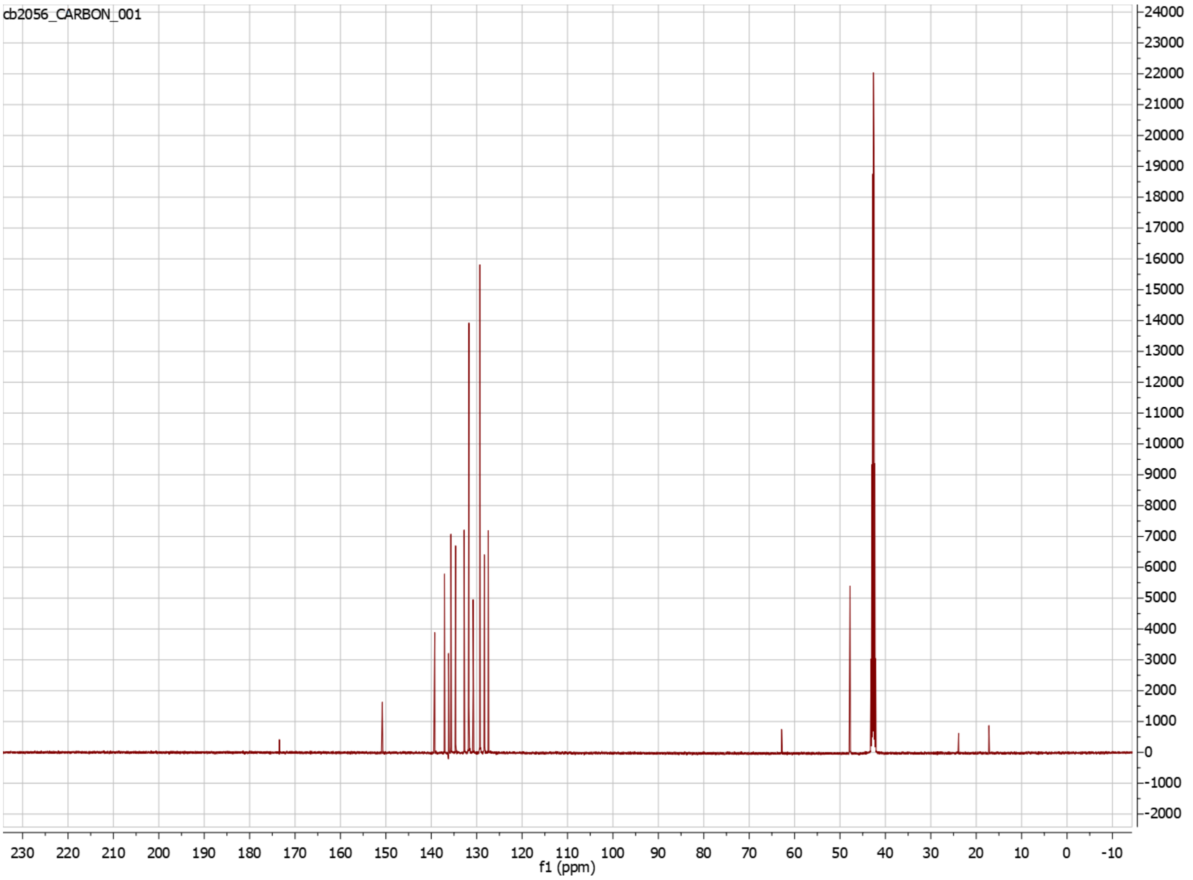

Spectrum compound 6

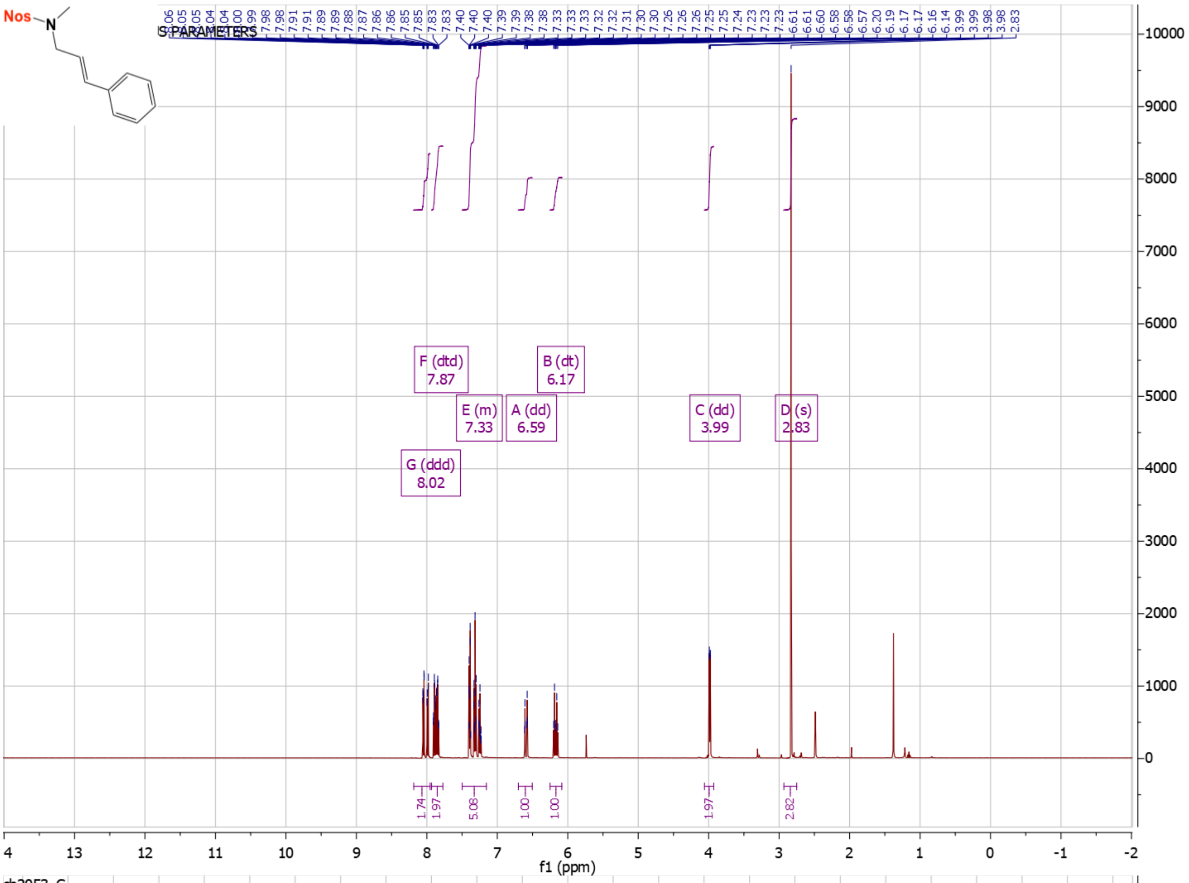

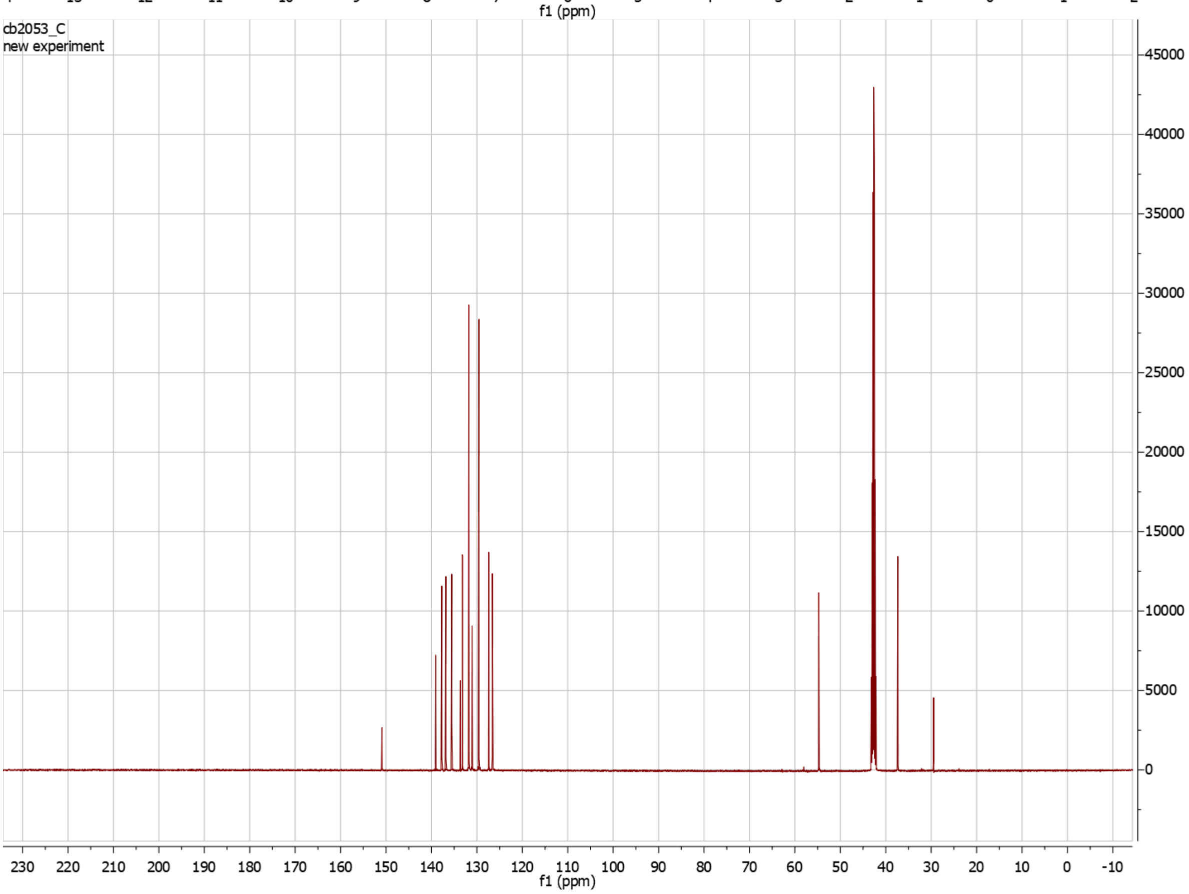

Spectrum compound 7

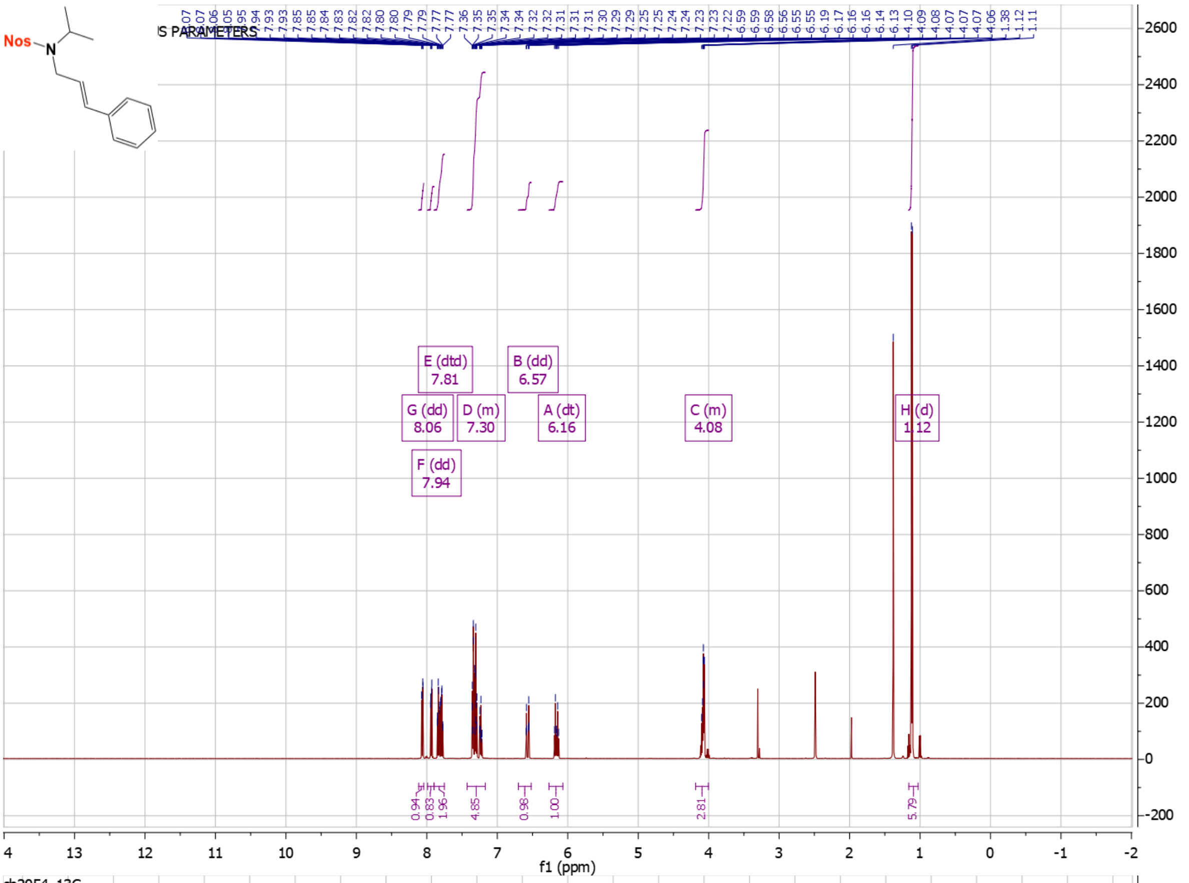

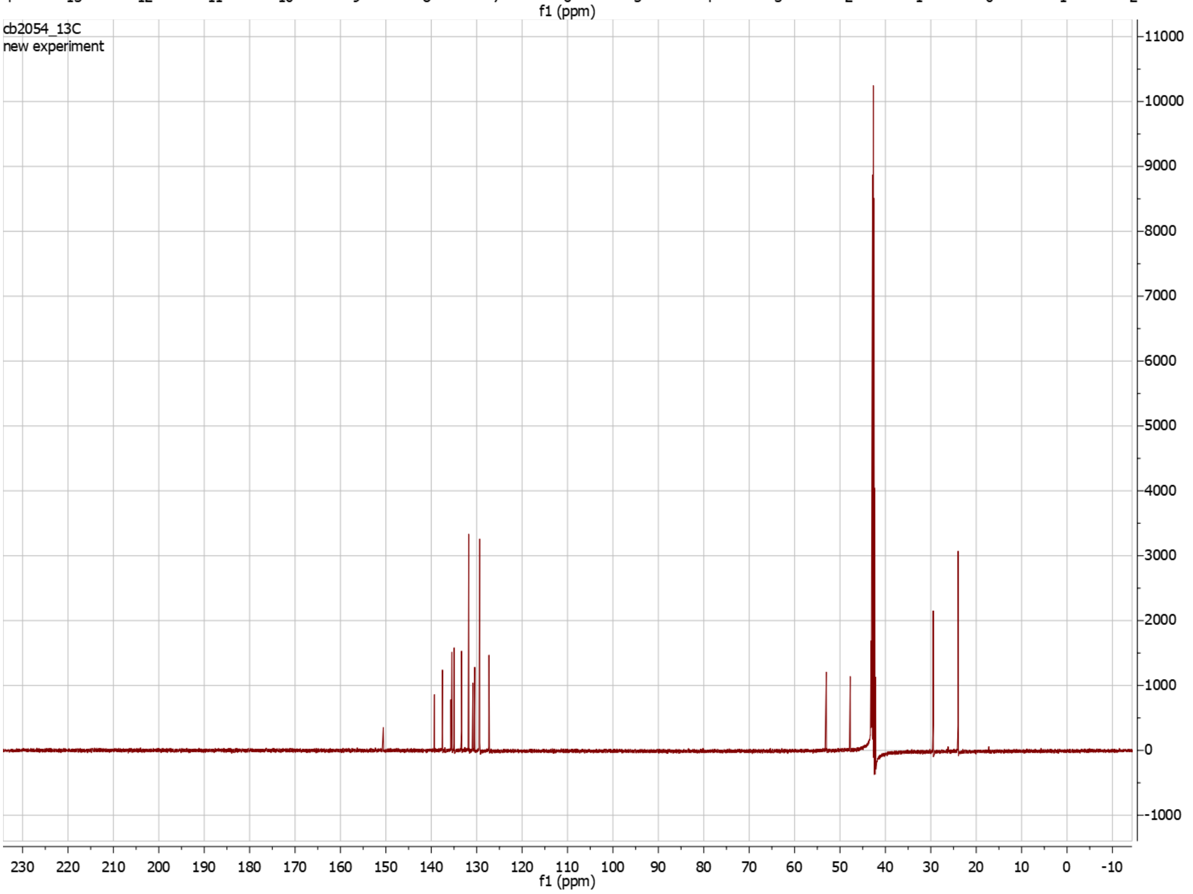

Spectrum compound 8

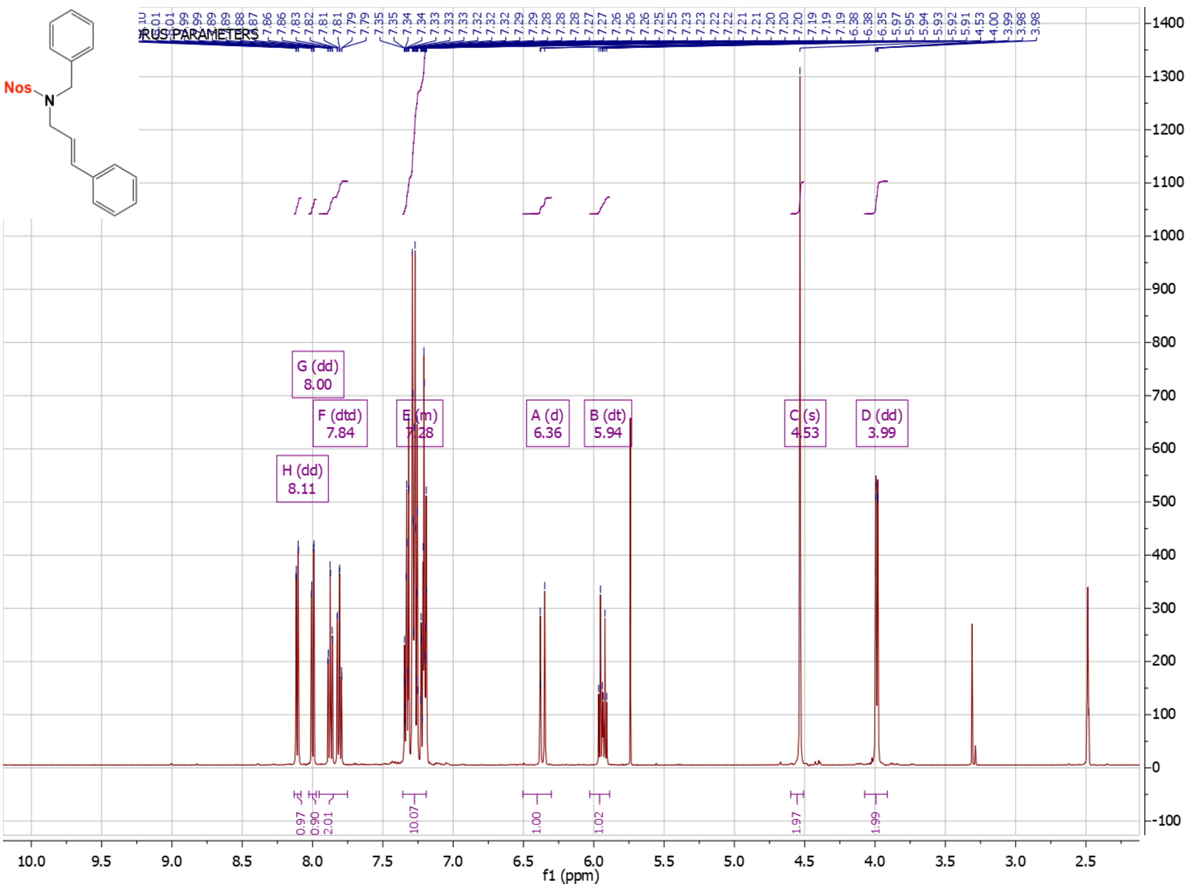

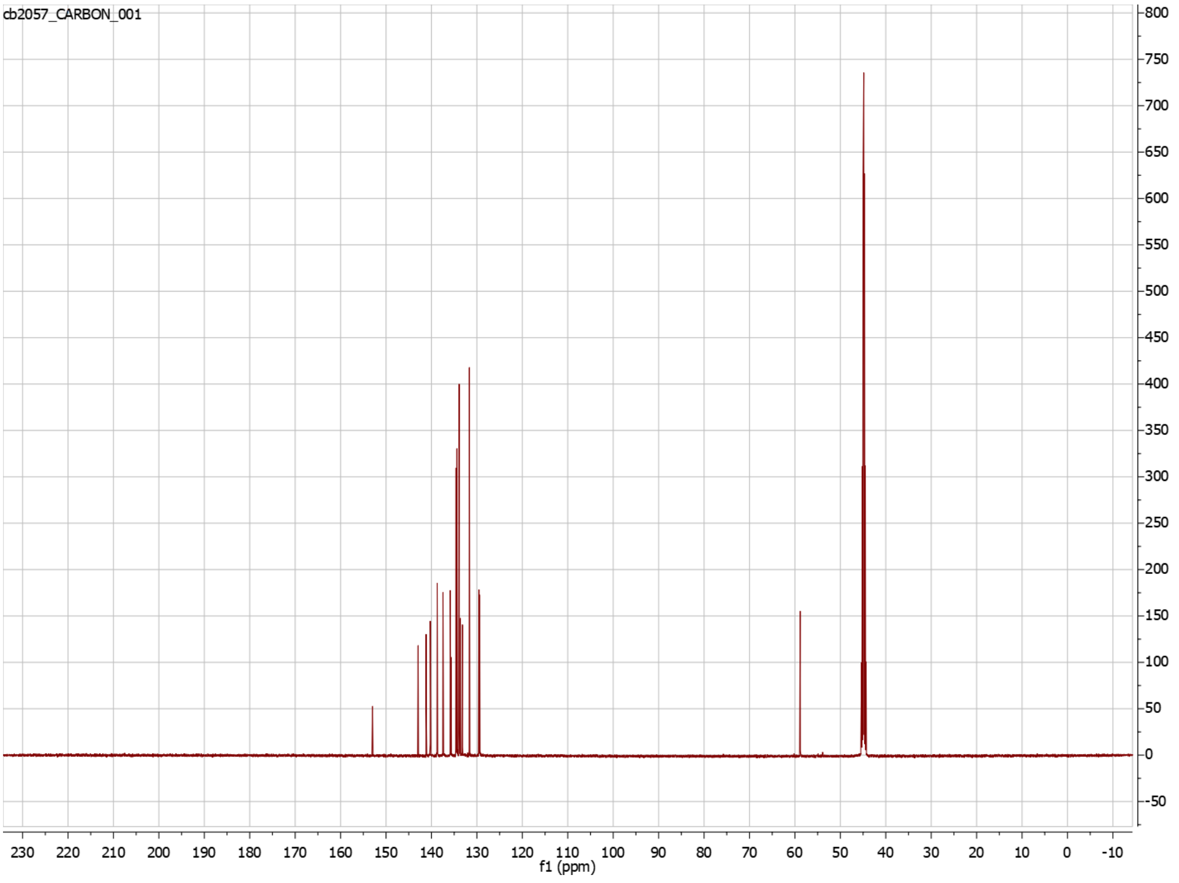

Spectrum compound 9

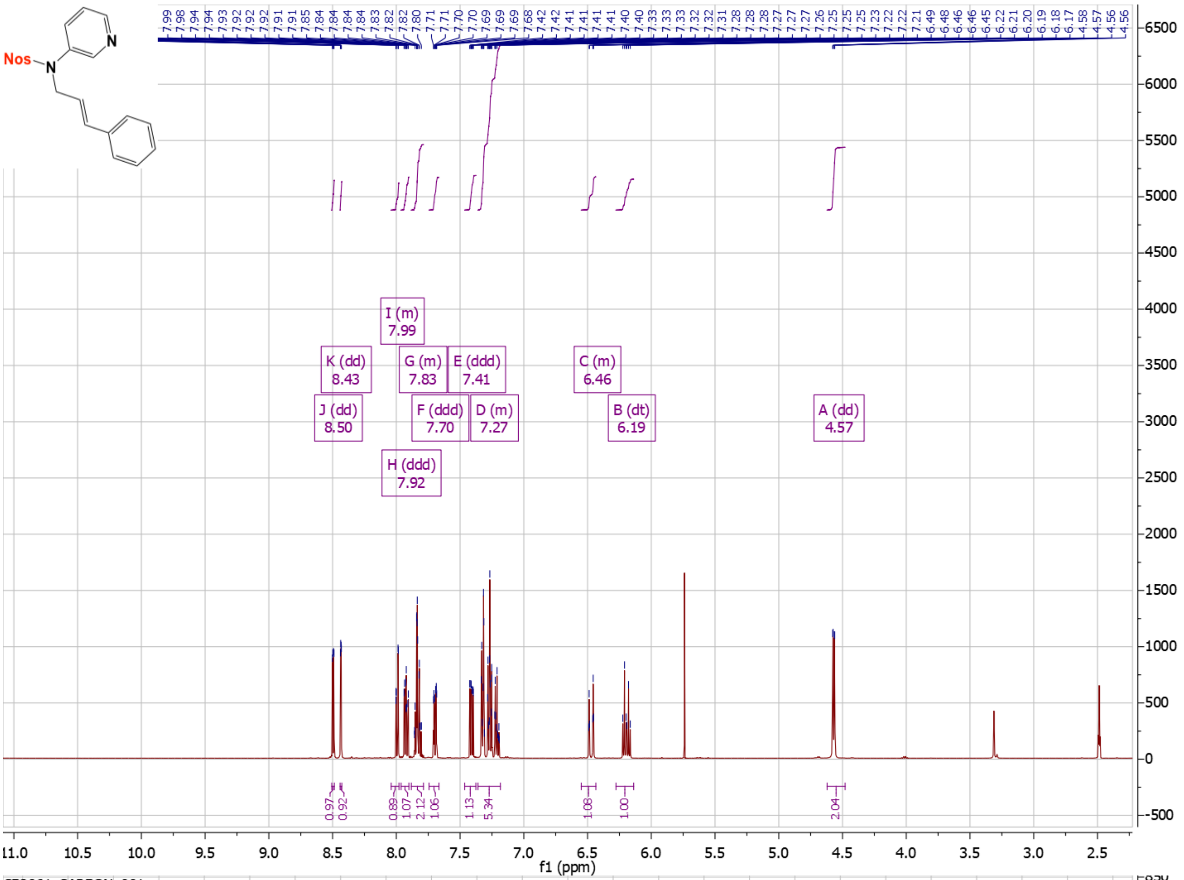

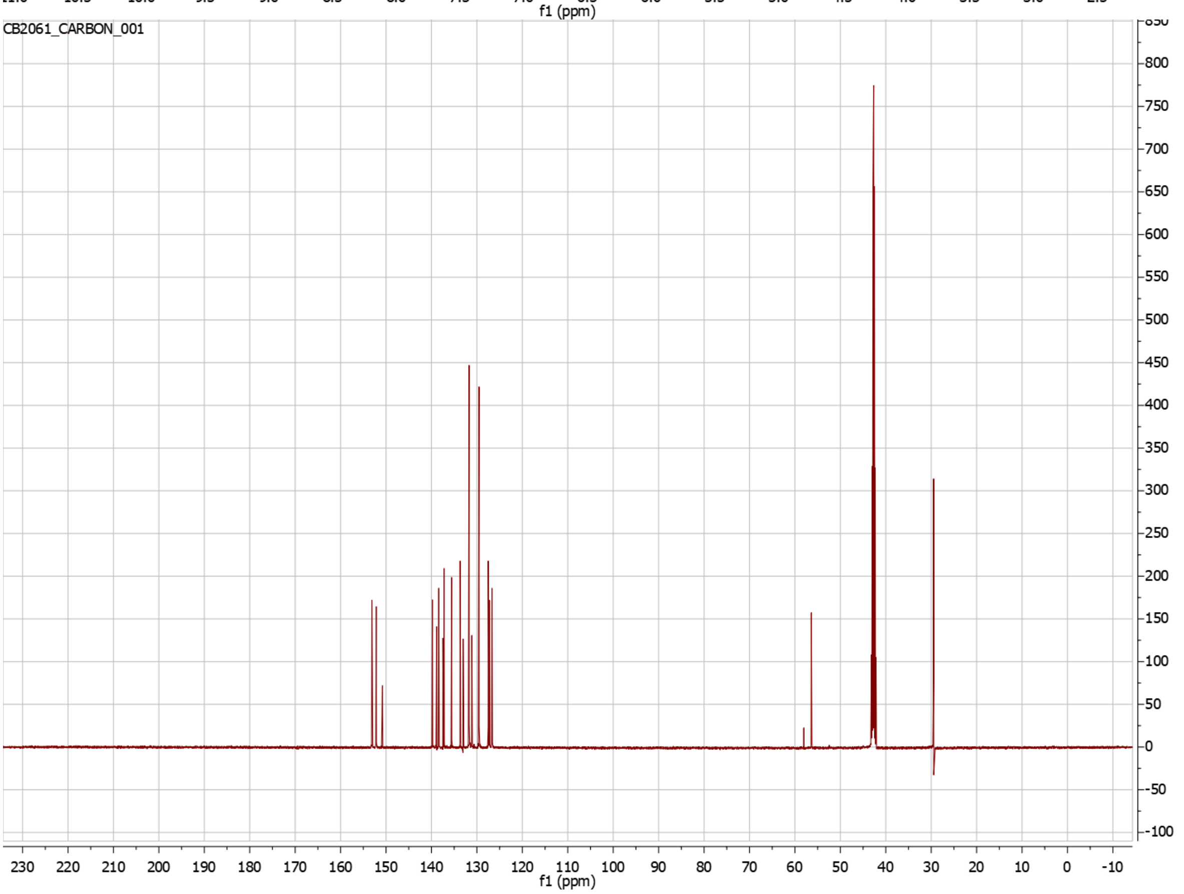

Spectrum compound 11

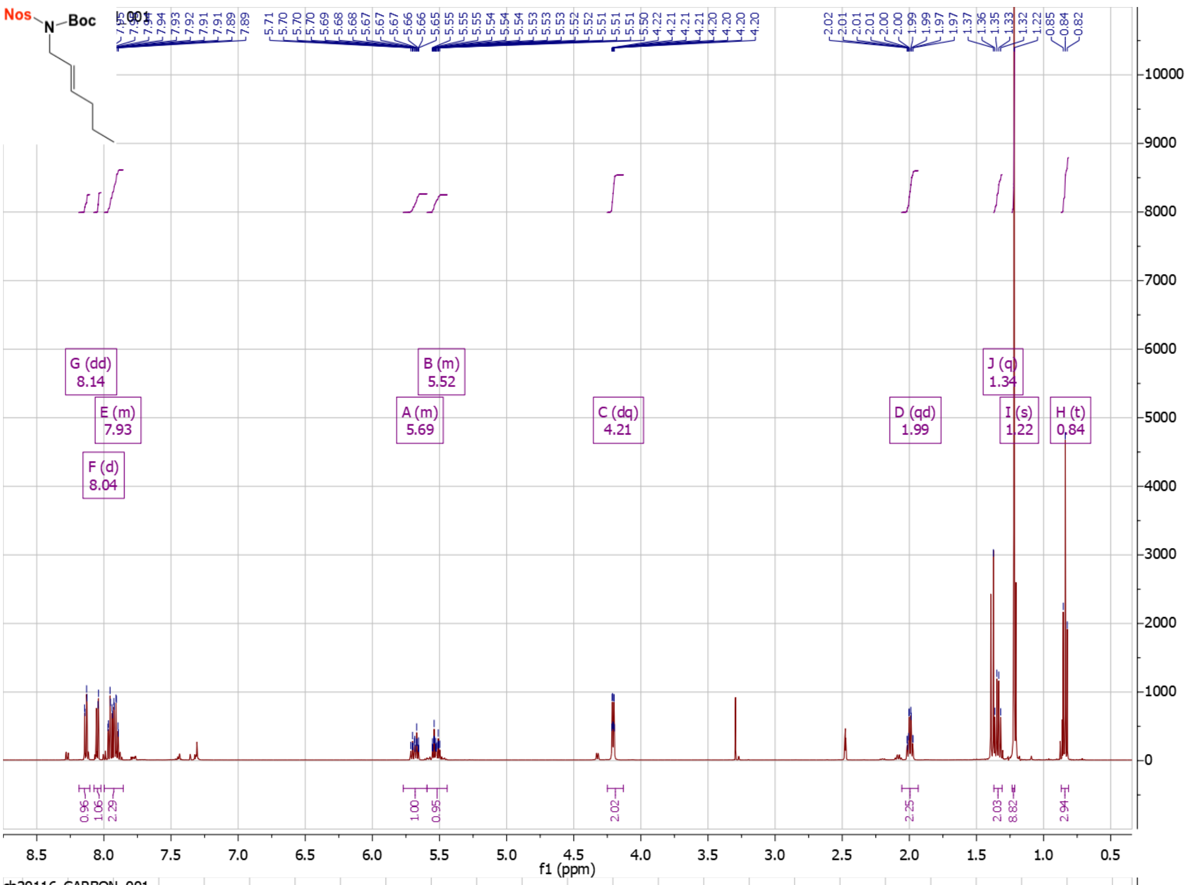

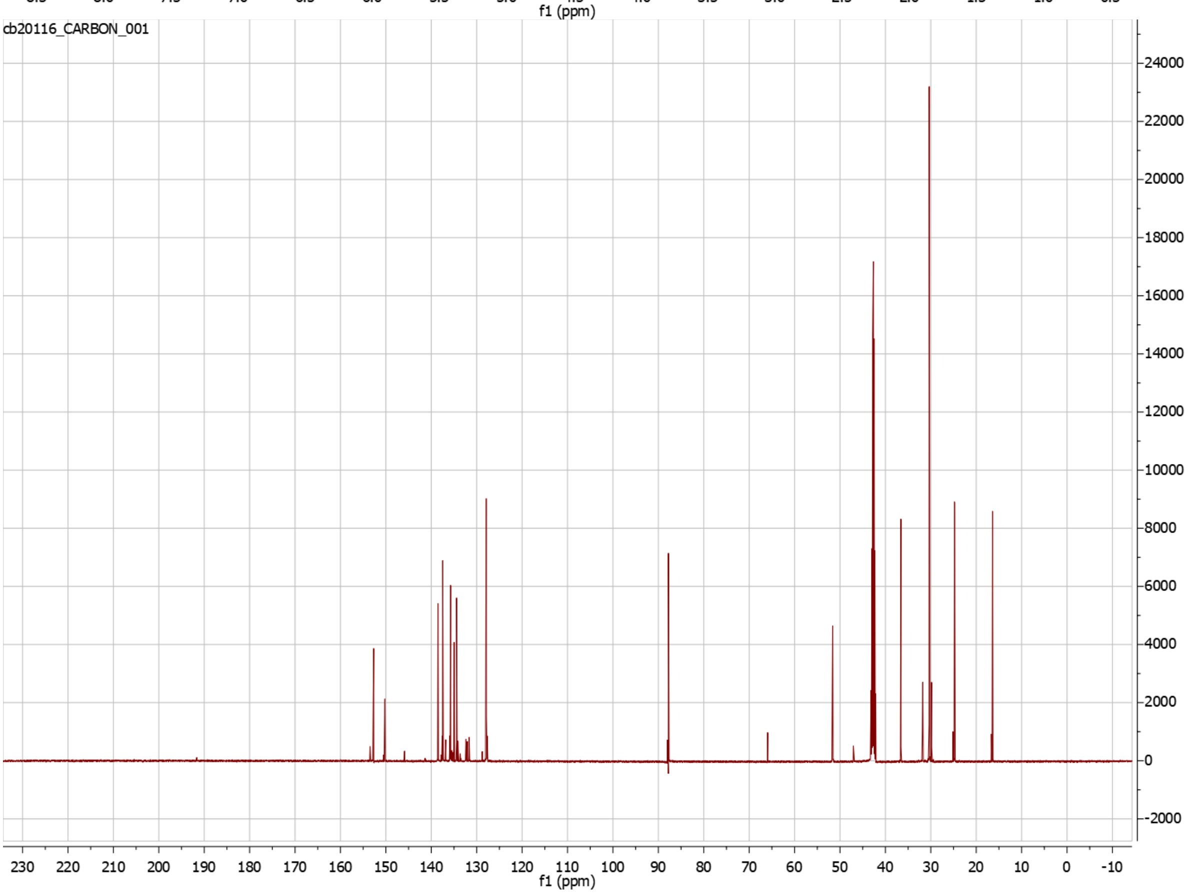

Spectrum compound 13

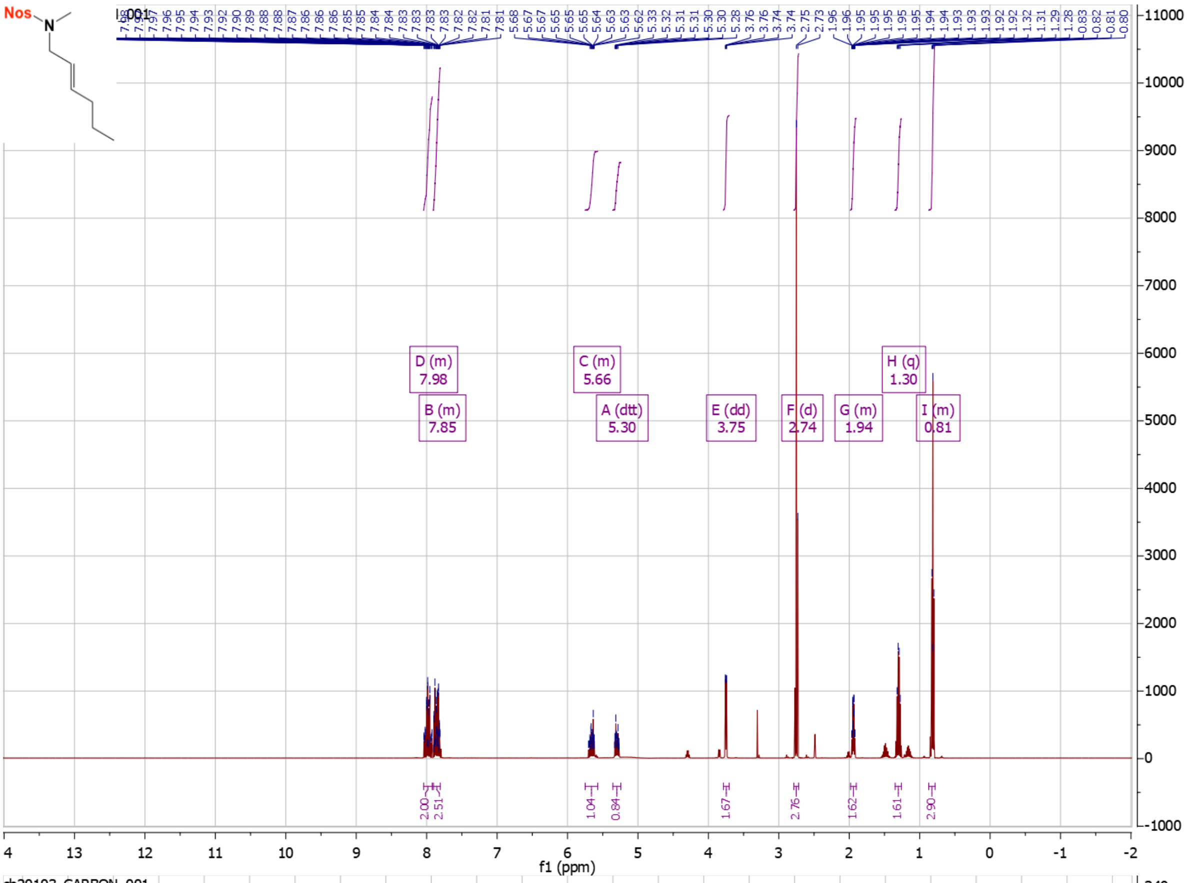

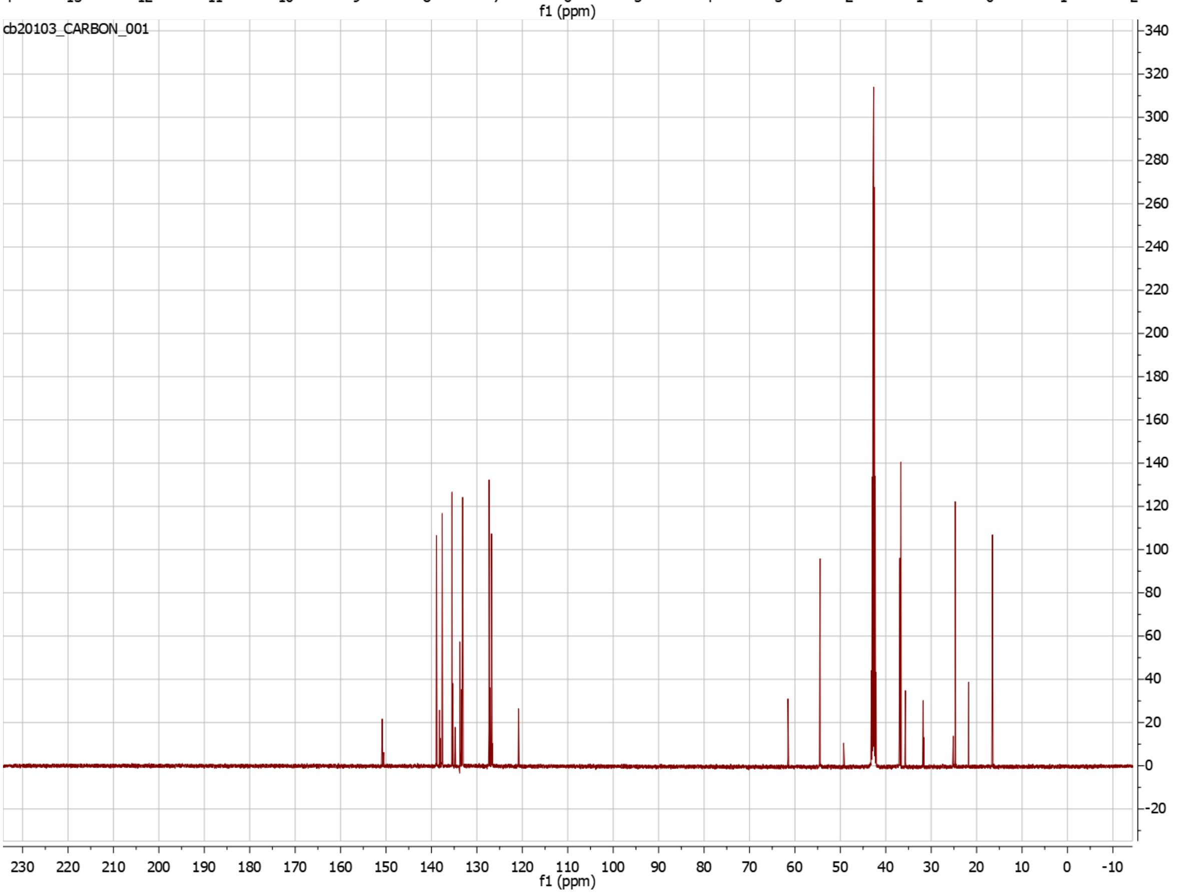

Spectrum compound 14

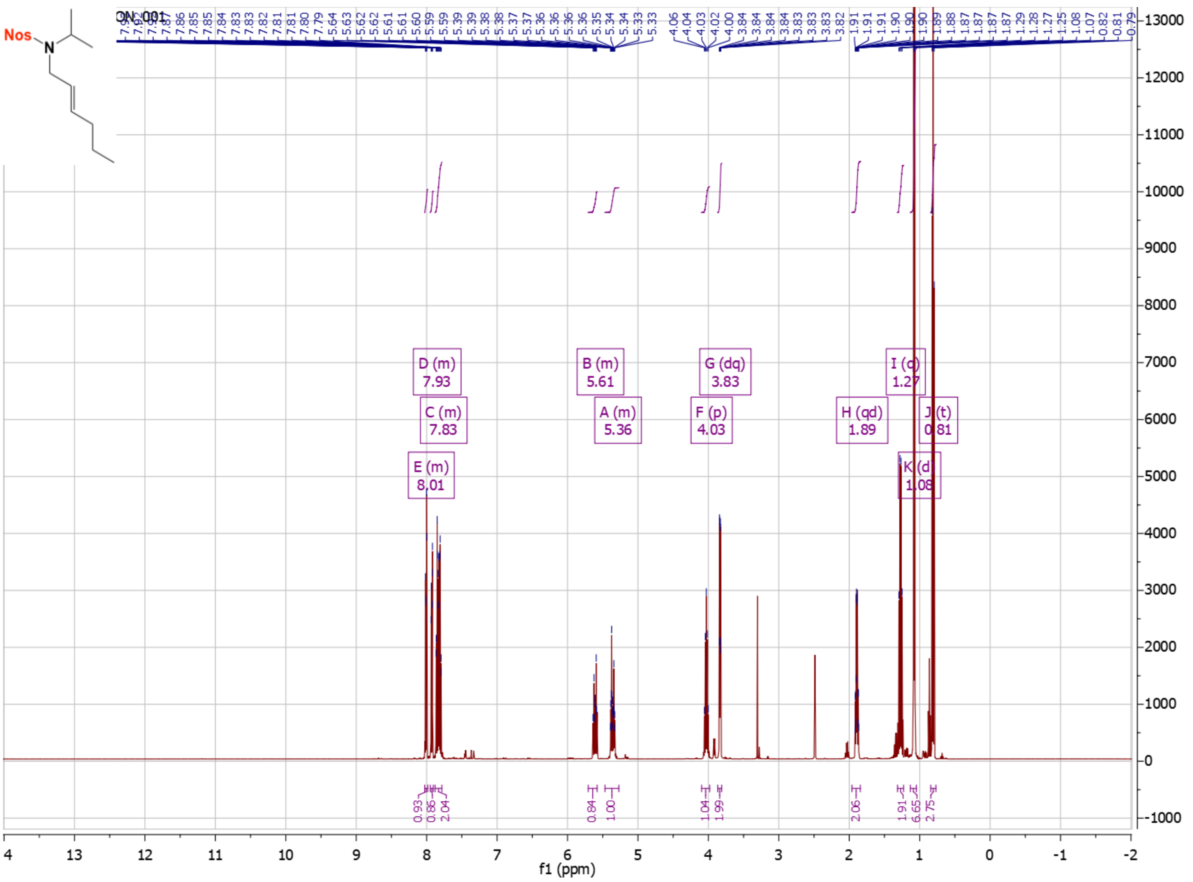

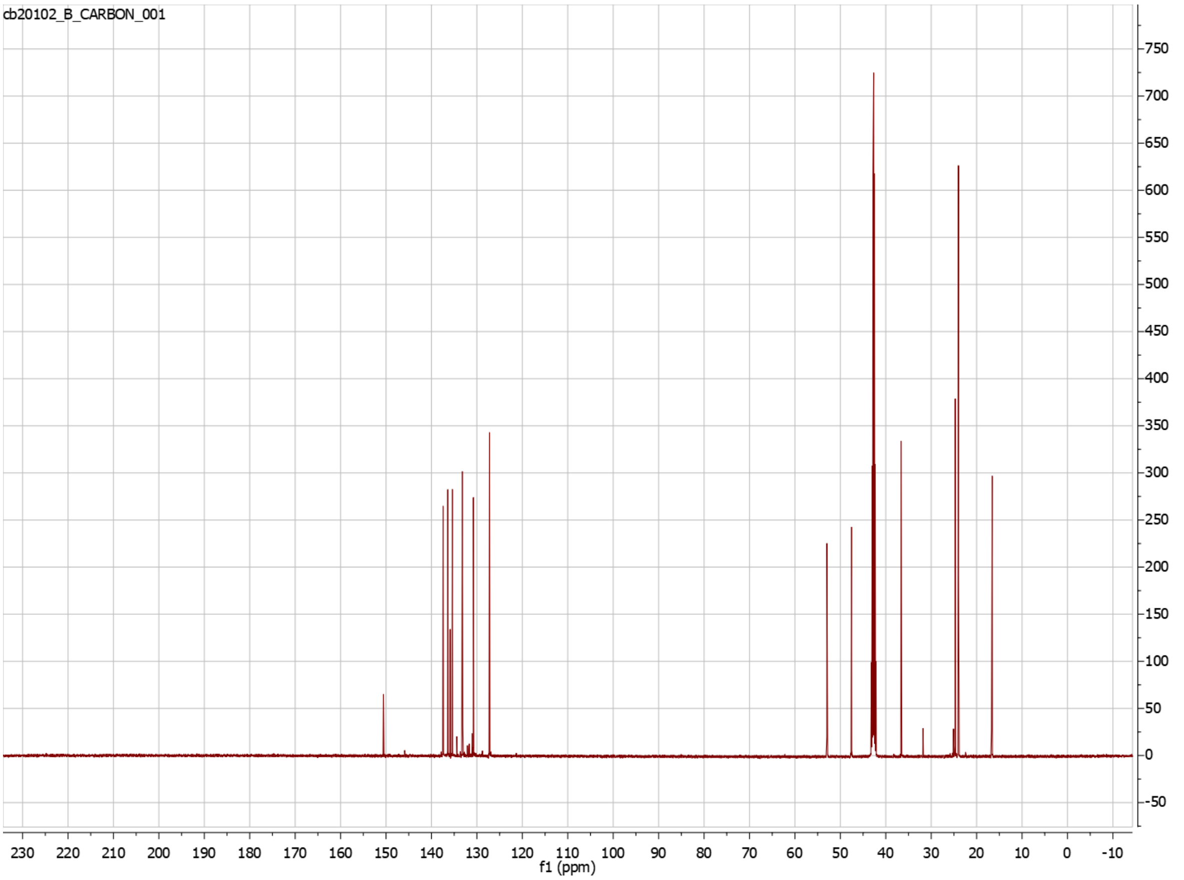

Spectrum compound 15

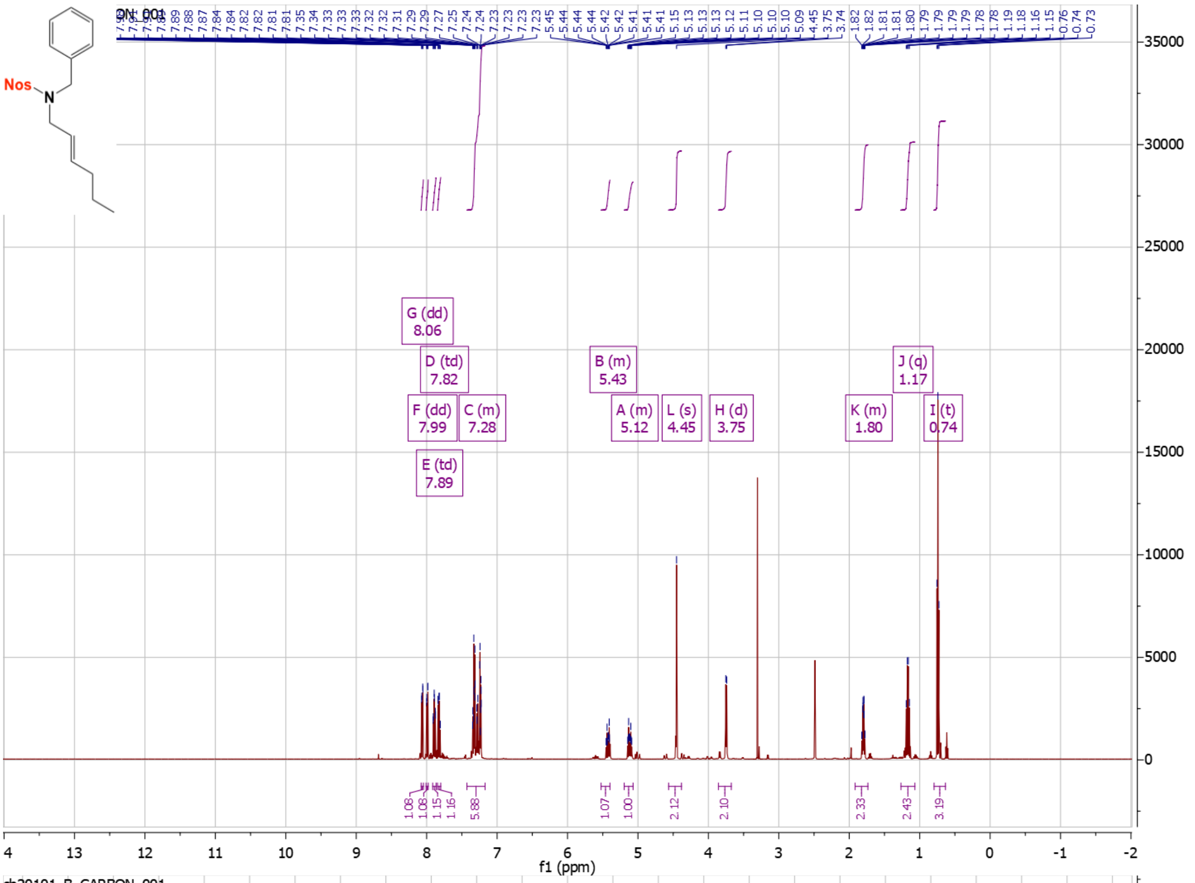

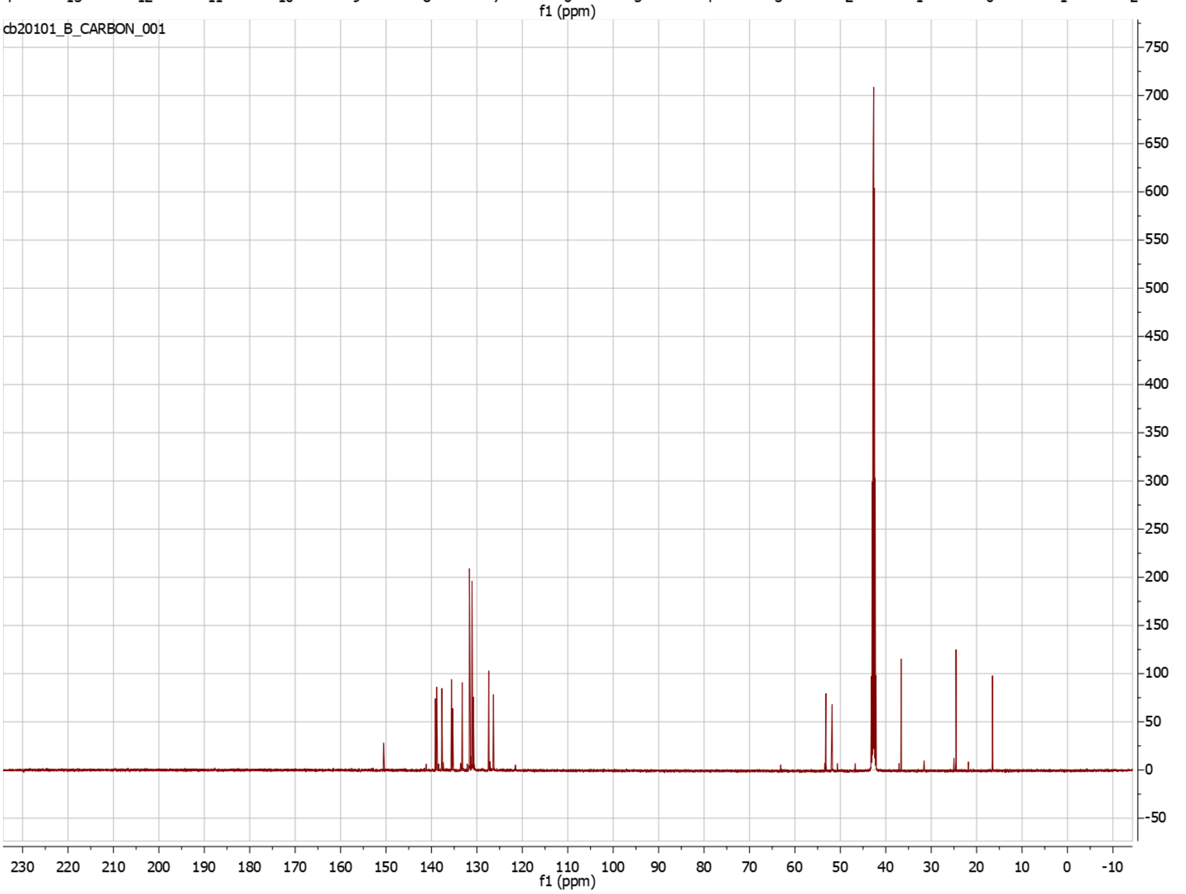

Spectrum compound 10

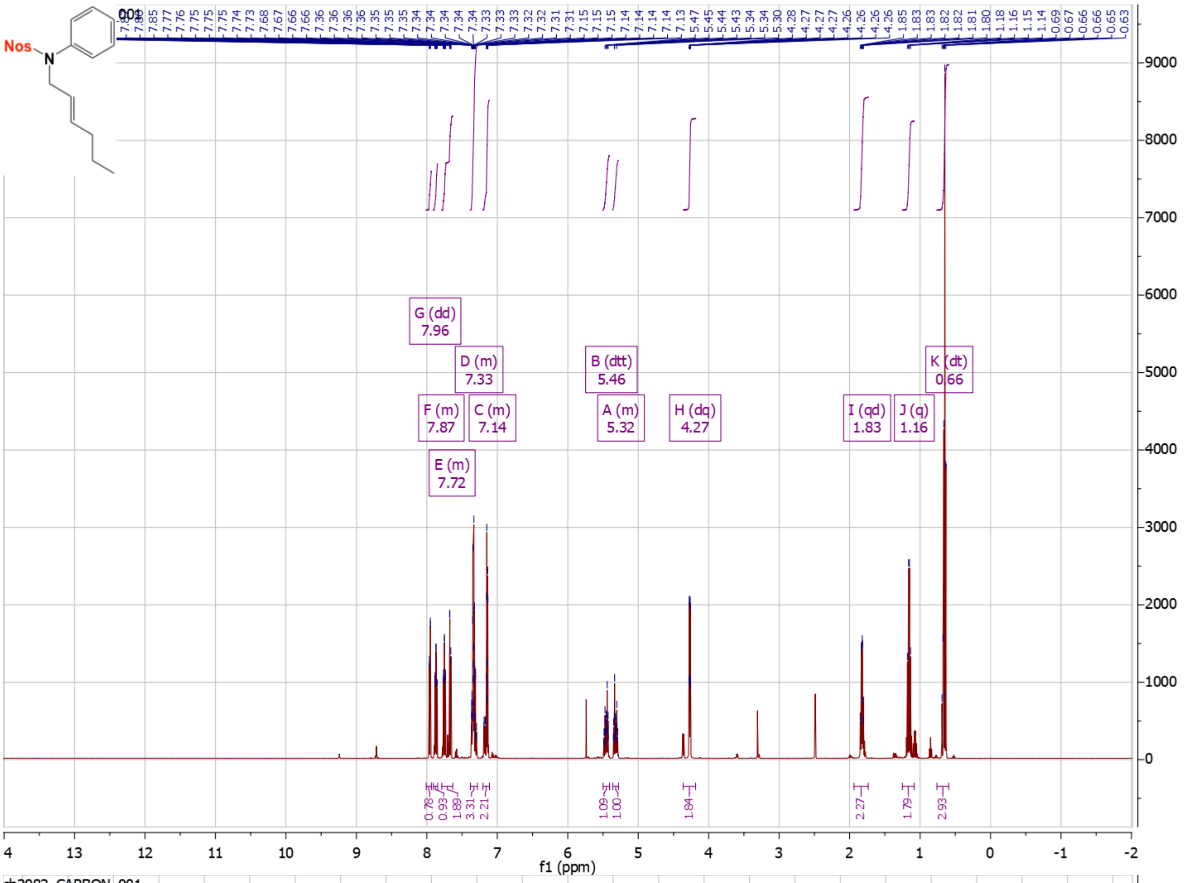

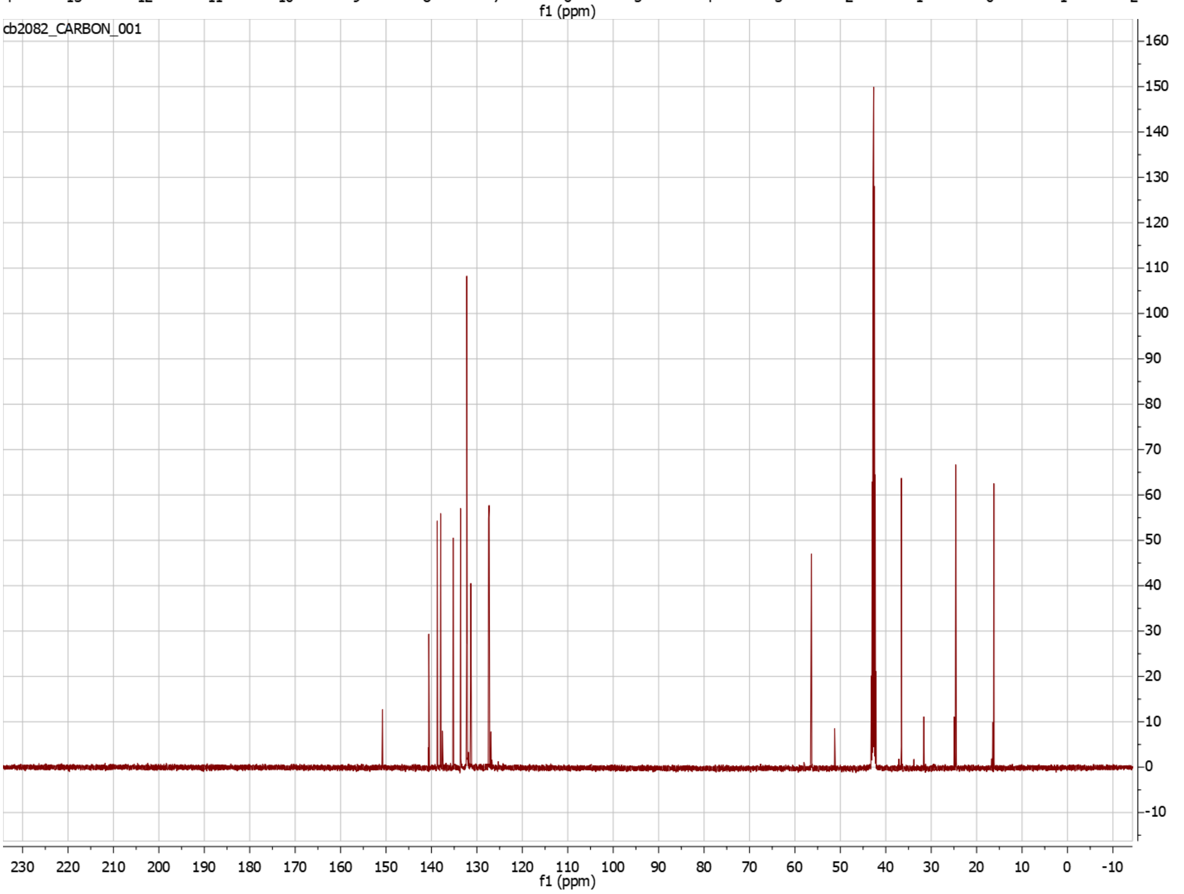

Spectrum compound 16

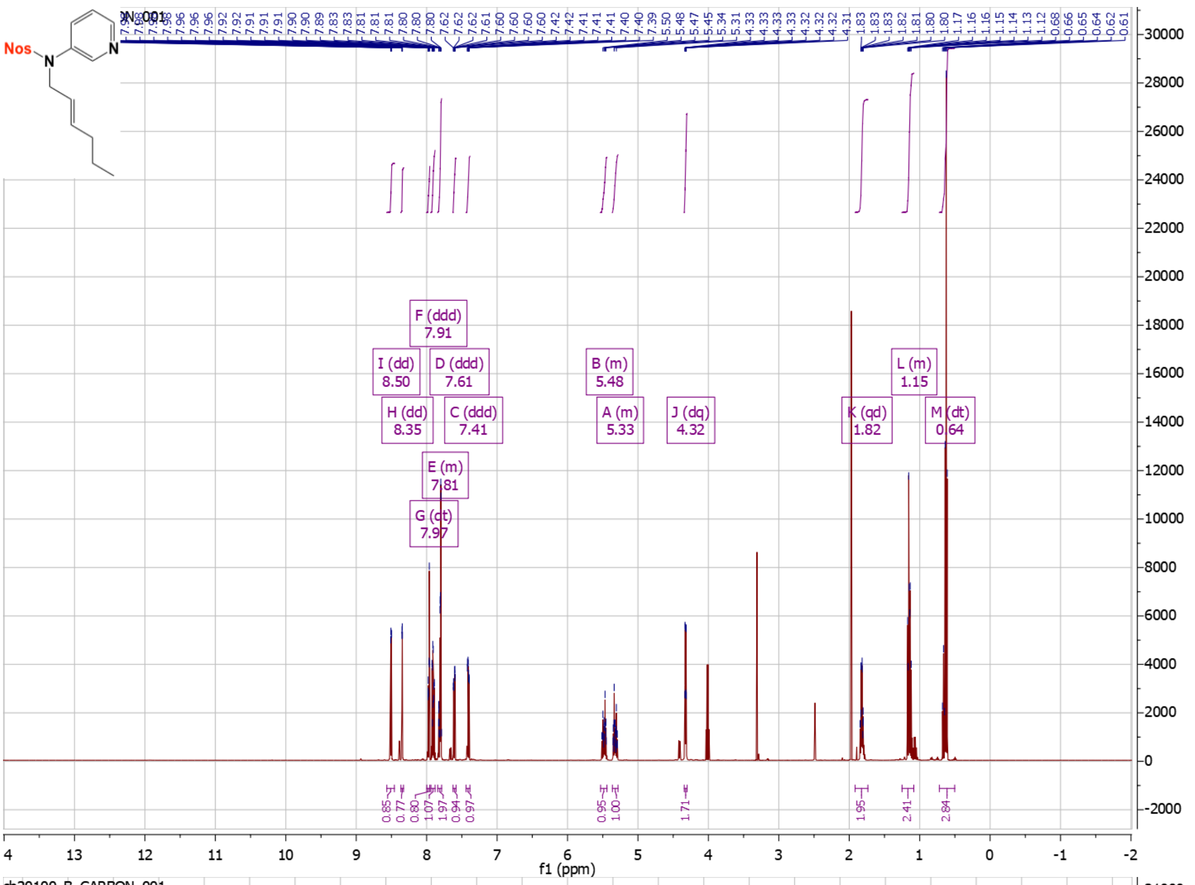

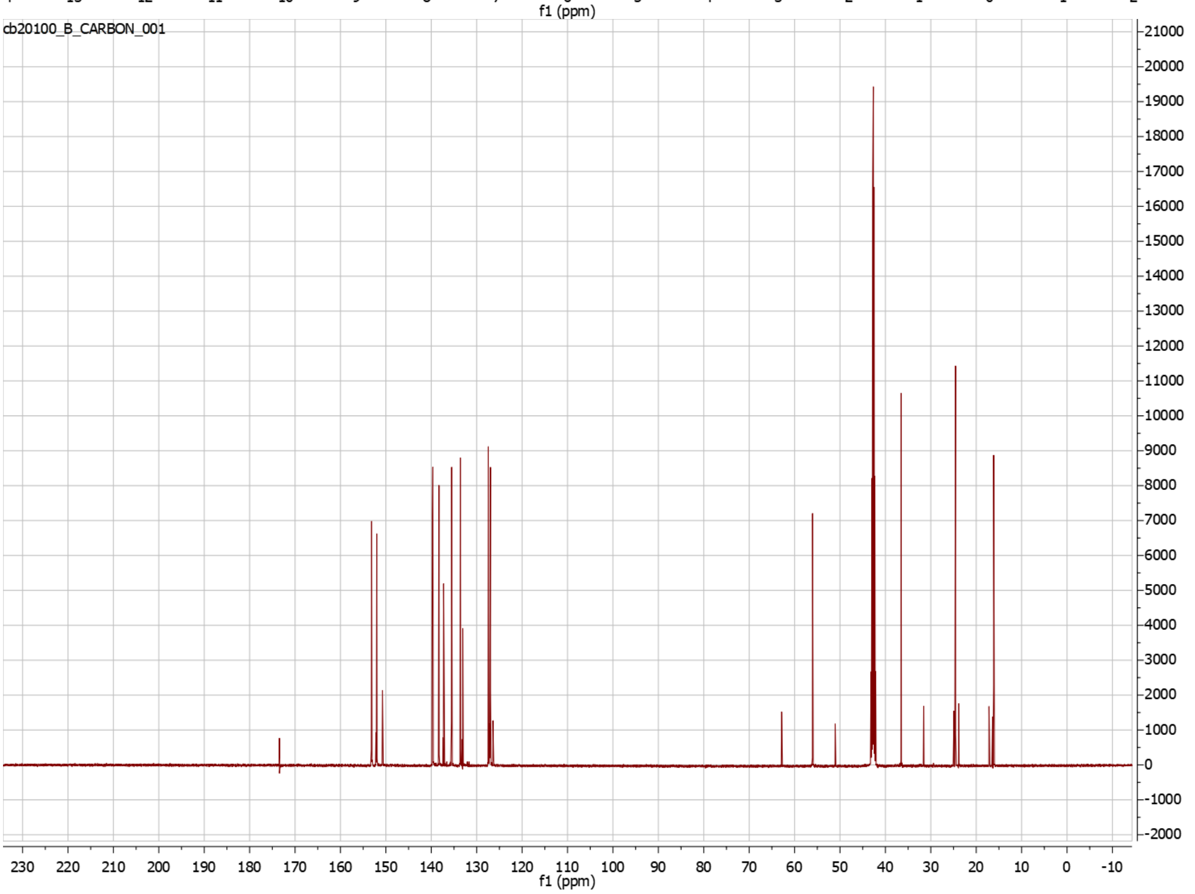

Spectrum compound 18

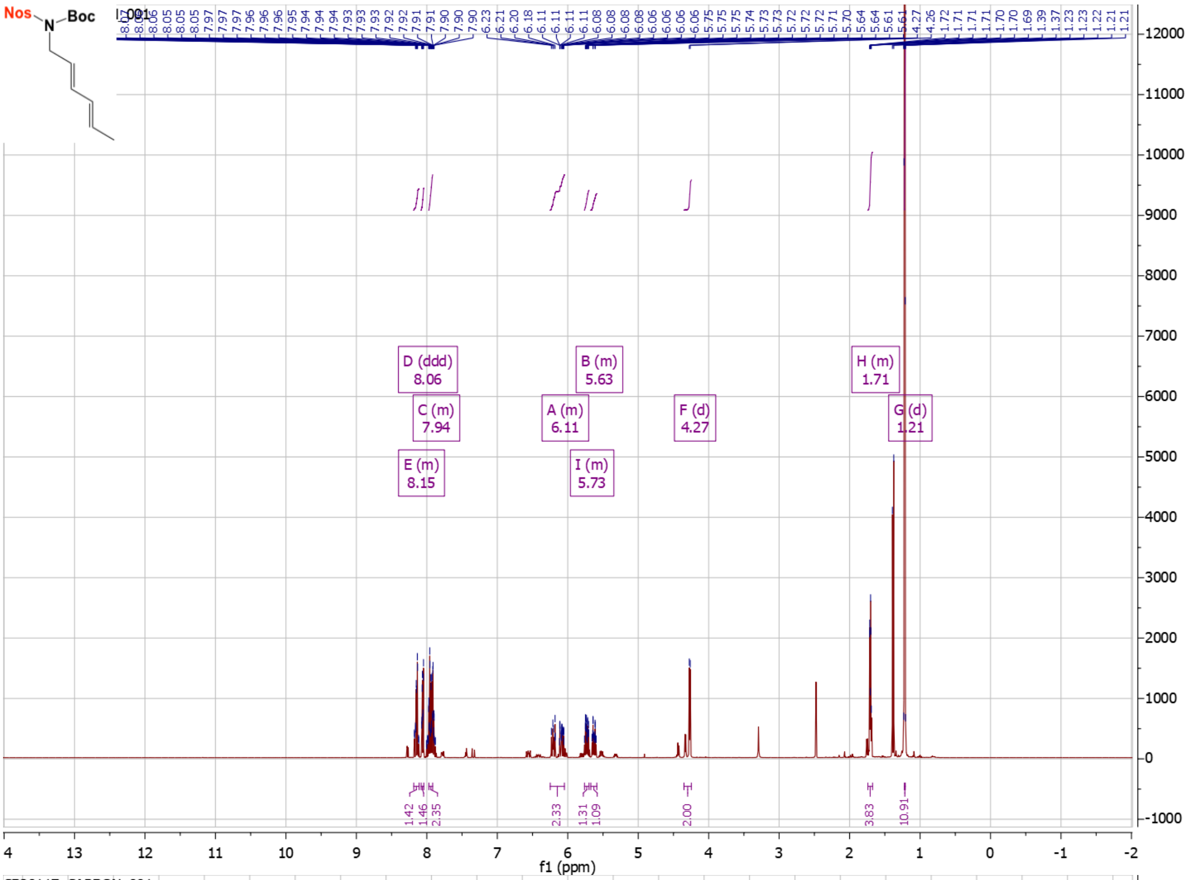

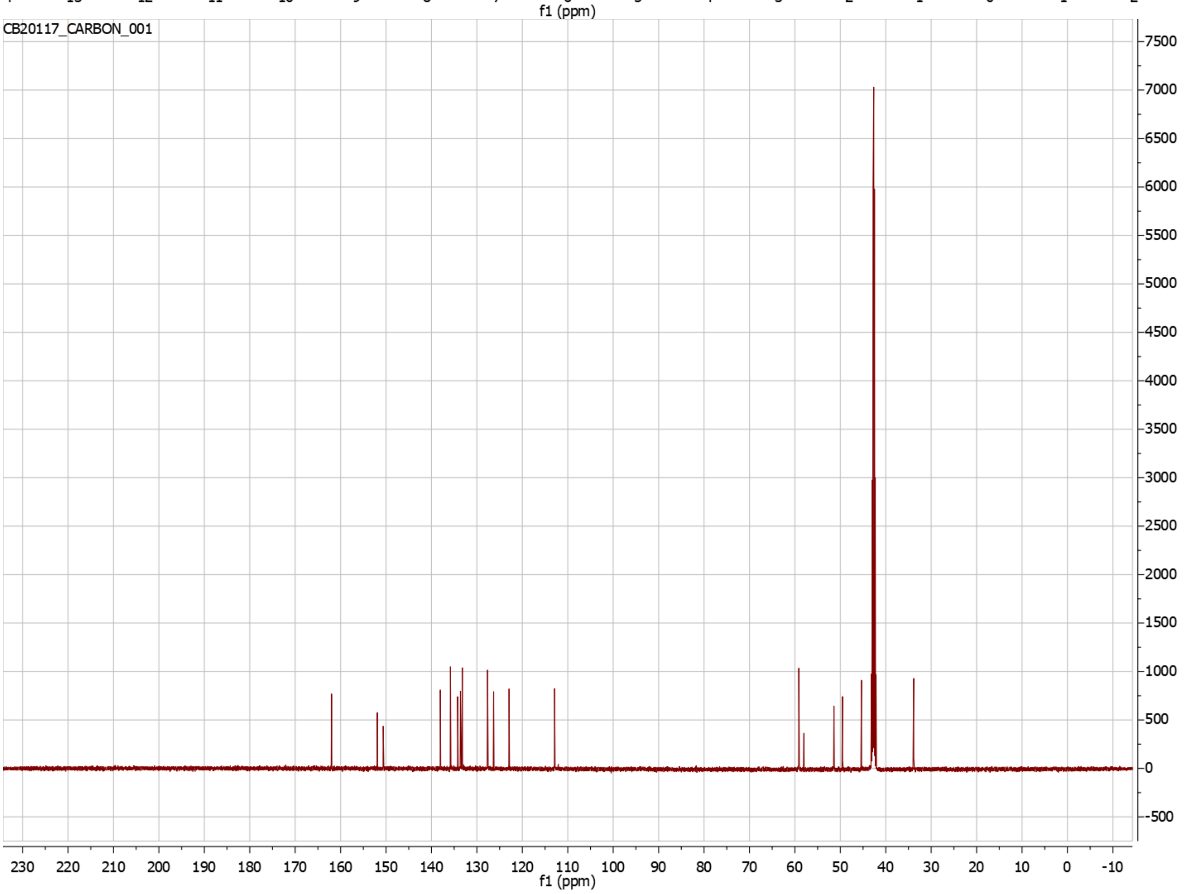

Spectrum compound 19

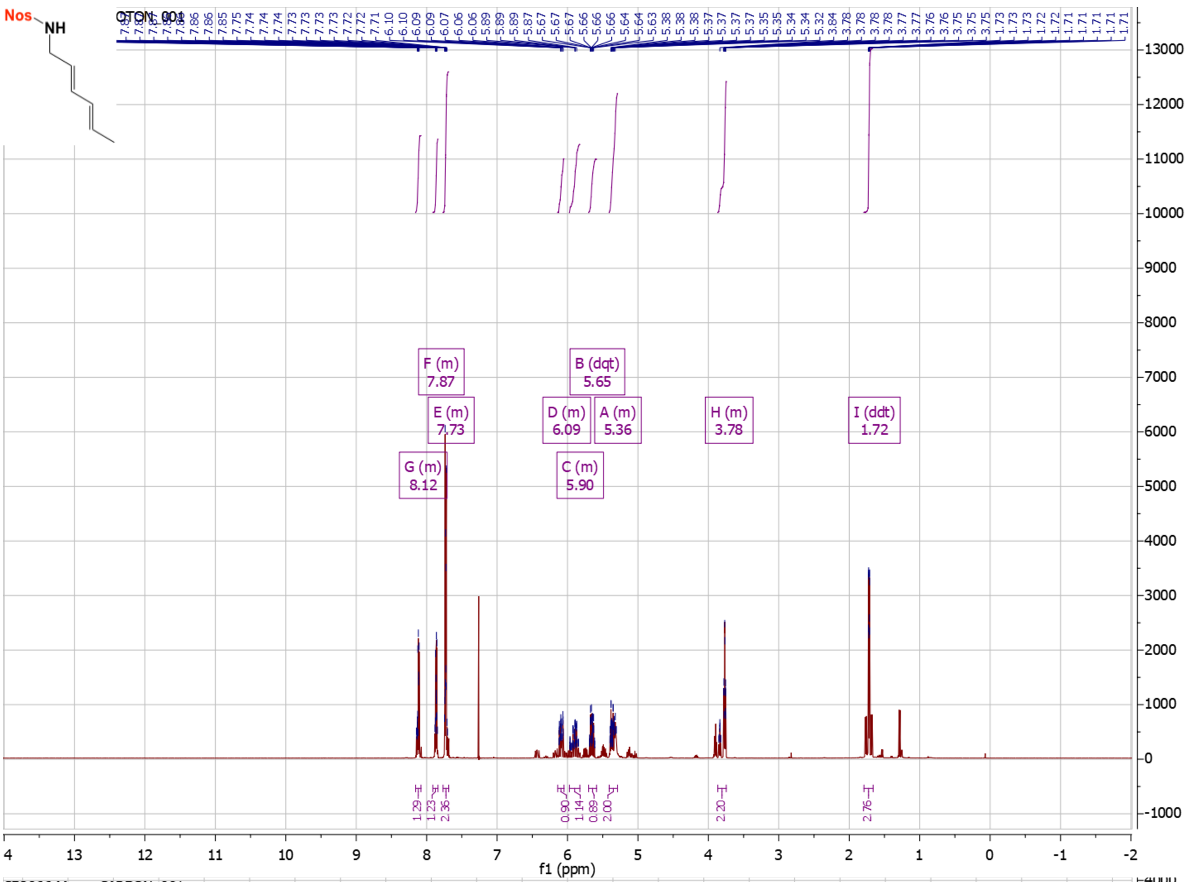

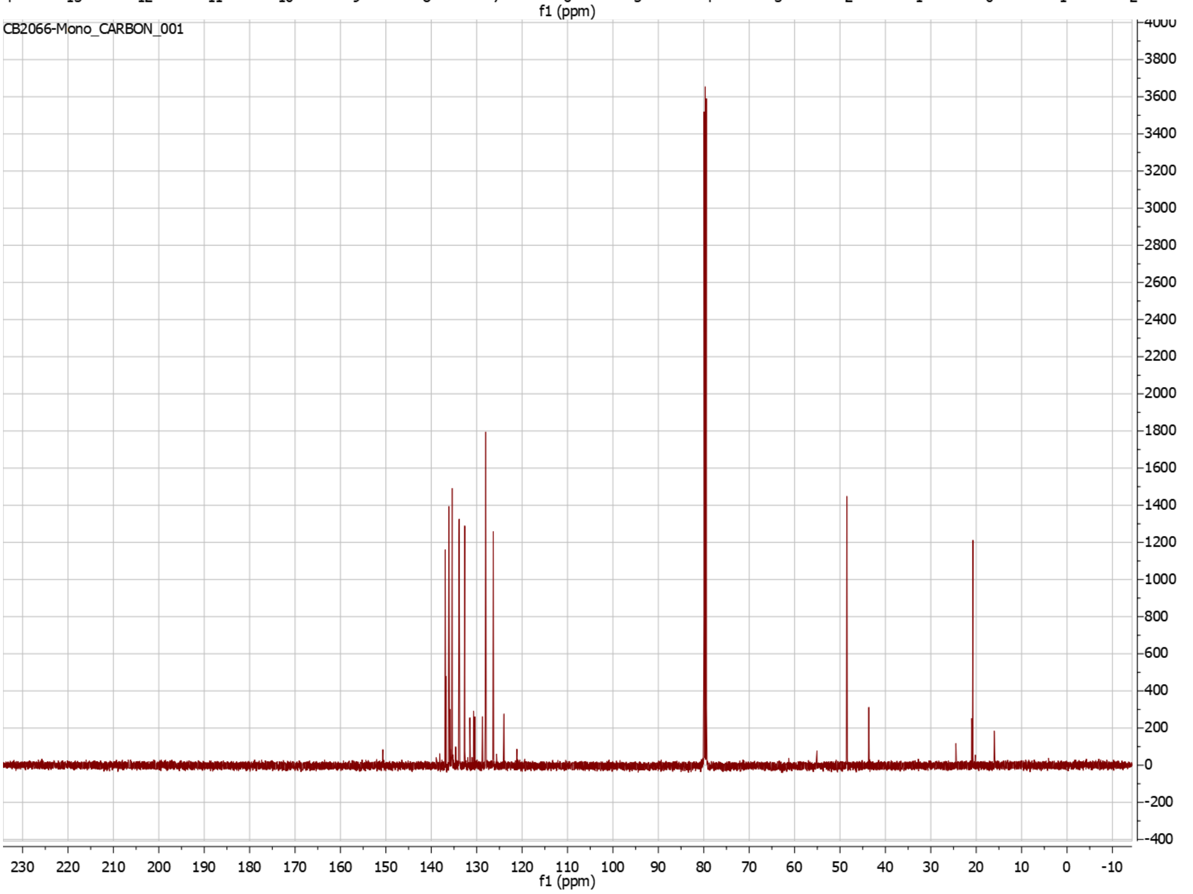

Spectrum compound 20

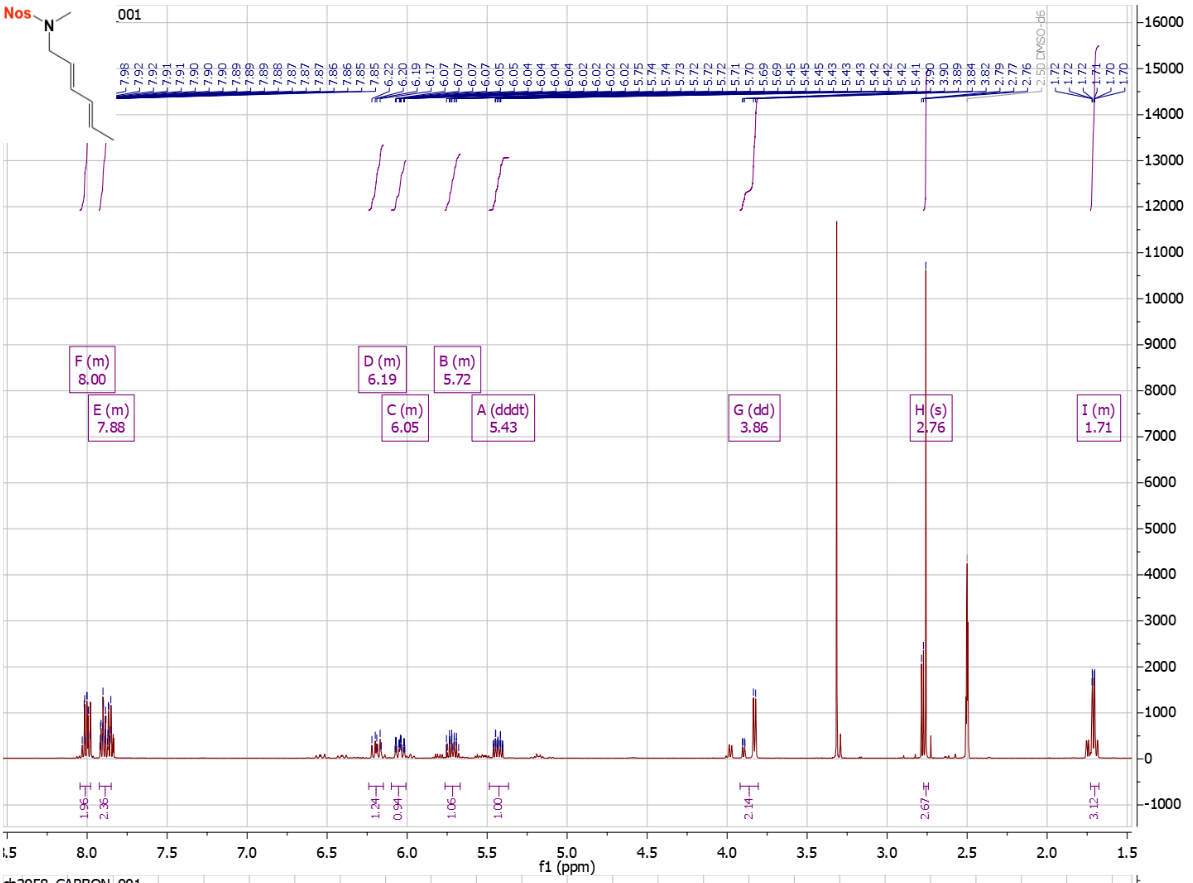

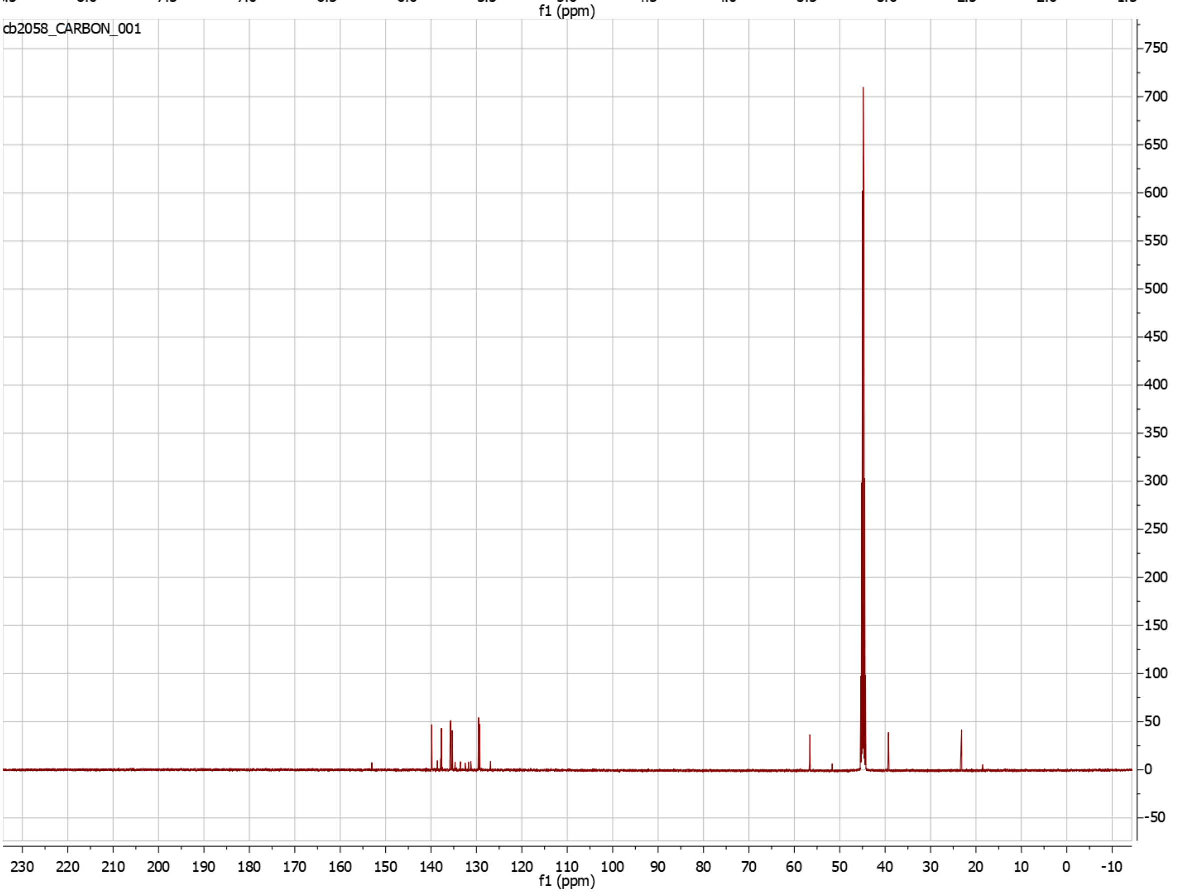

Spectrum compound 21

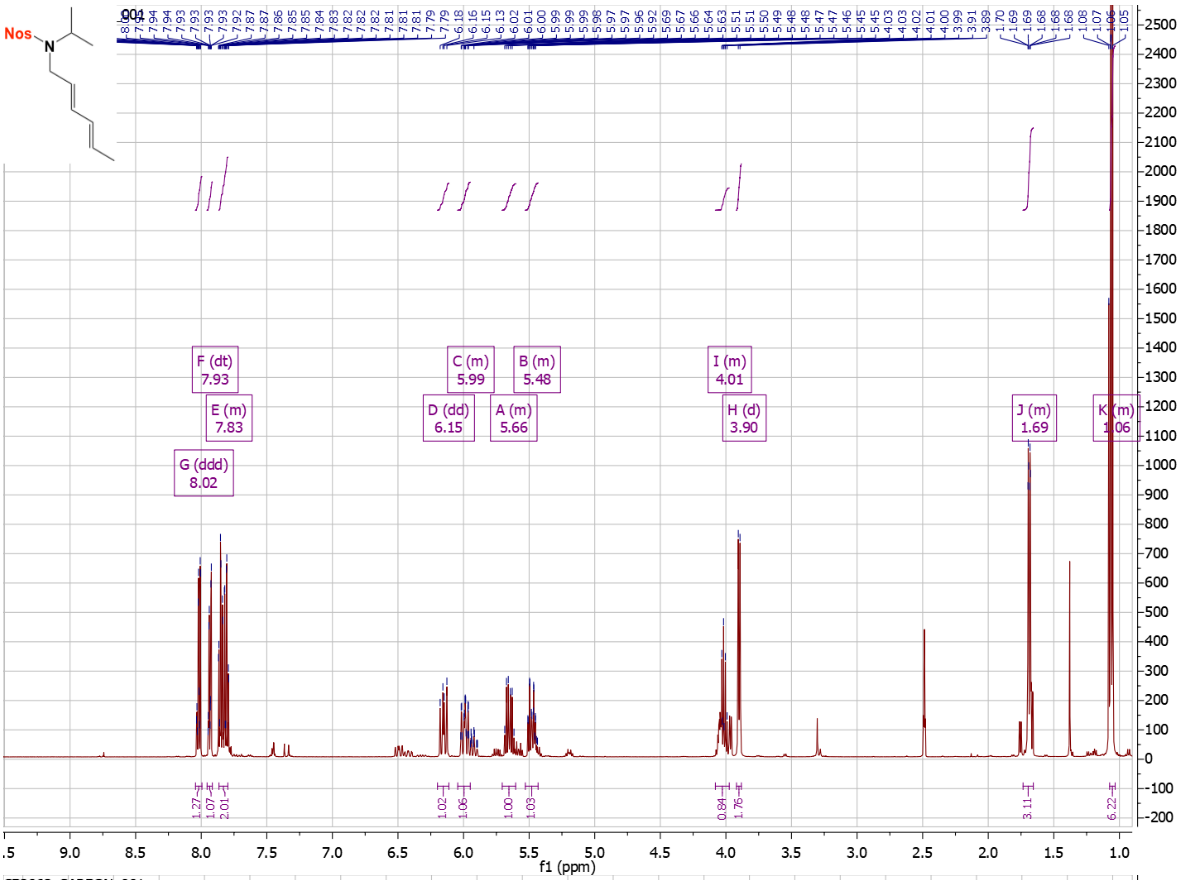

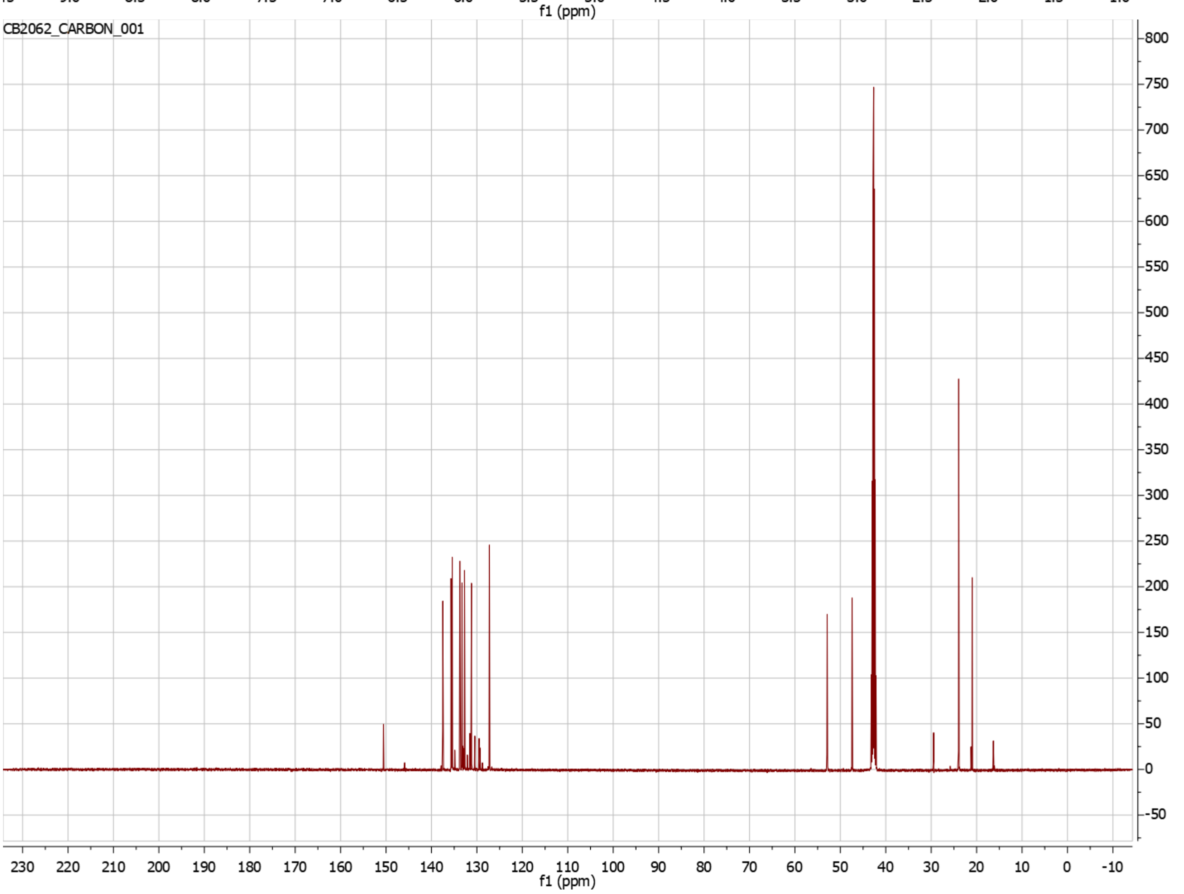

Spectrum compound 22

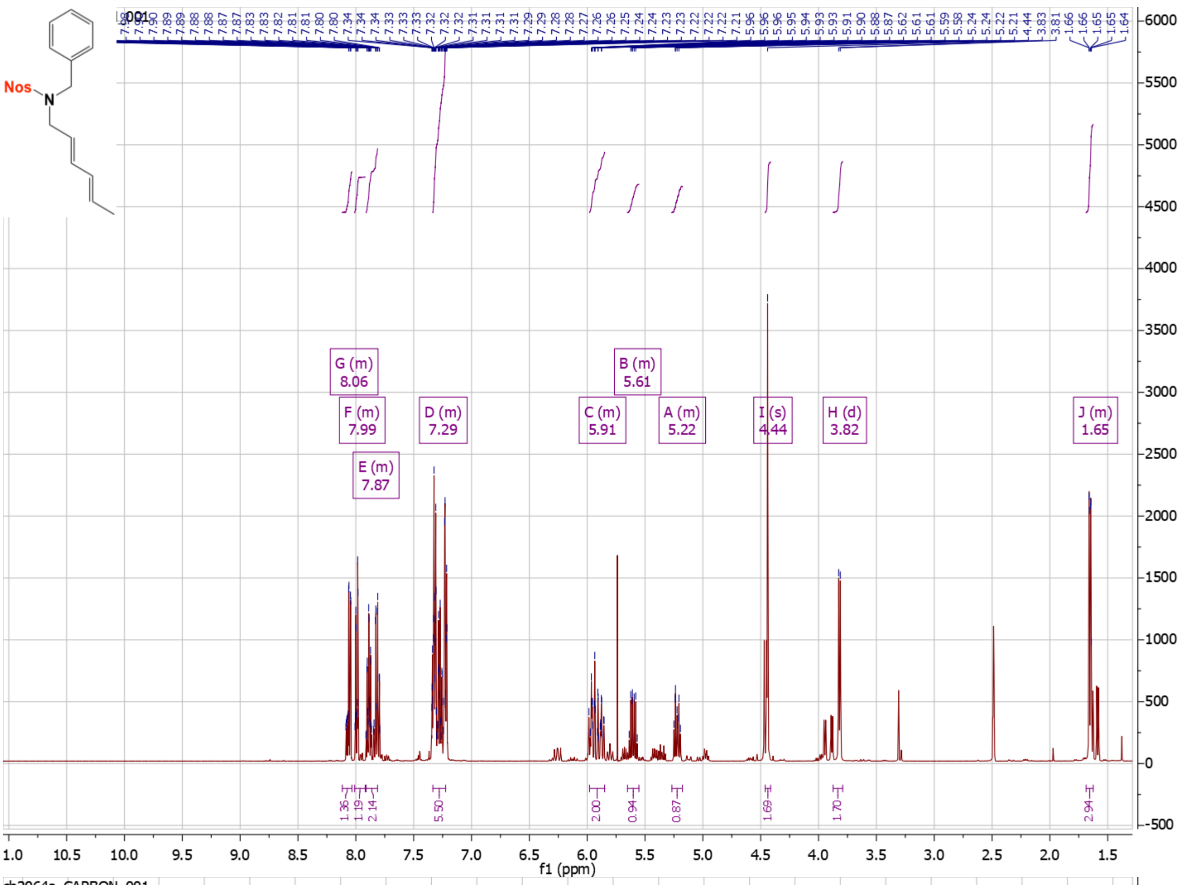

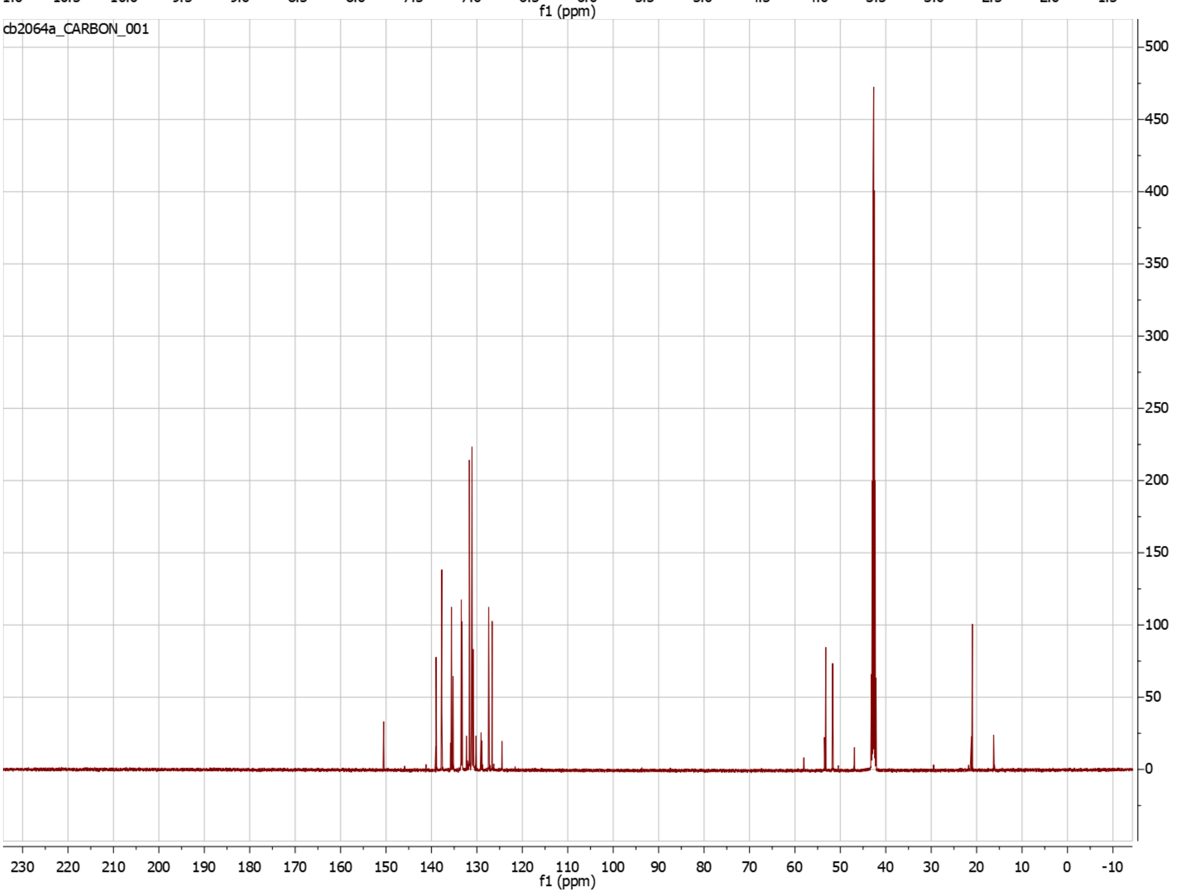

Spectrum compound 17

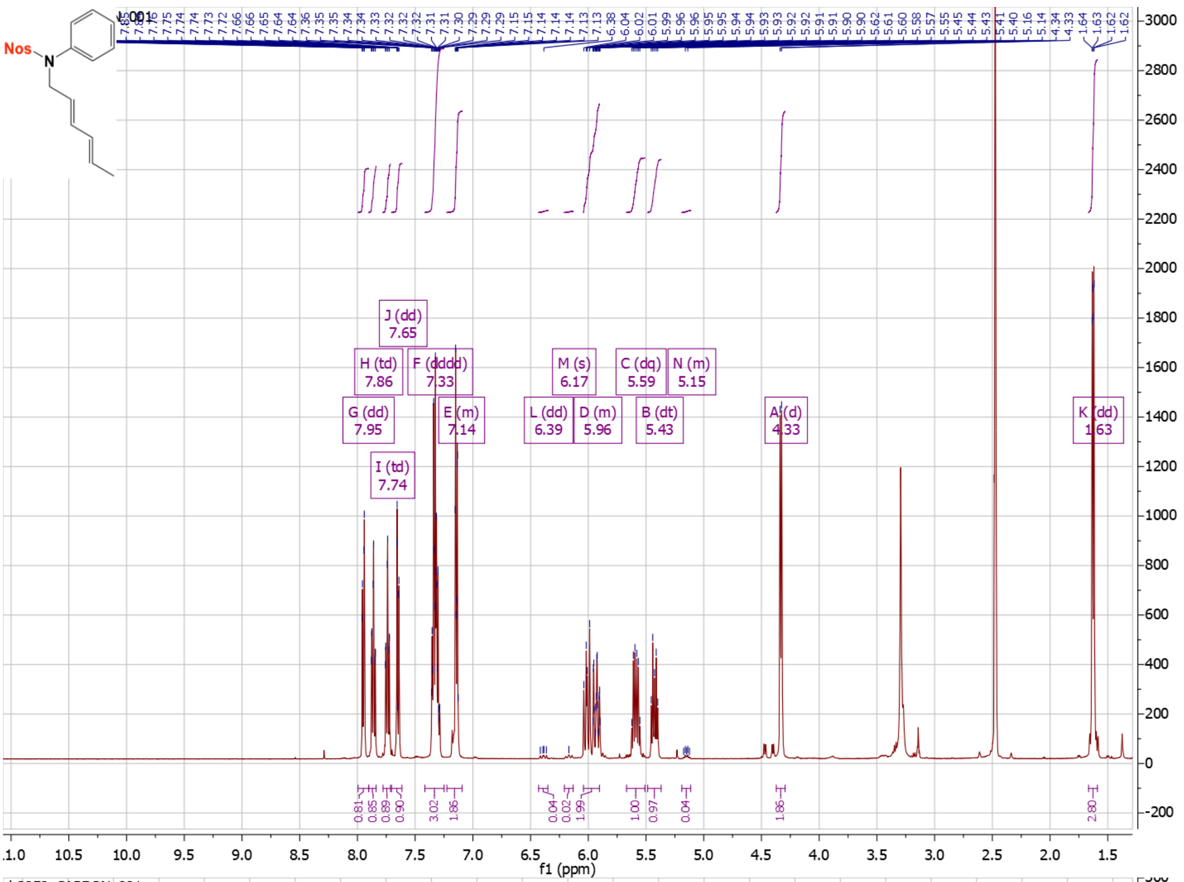

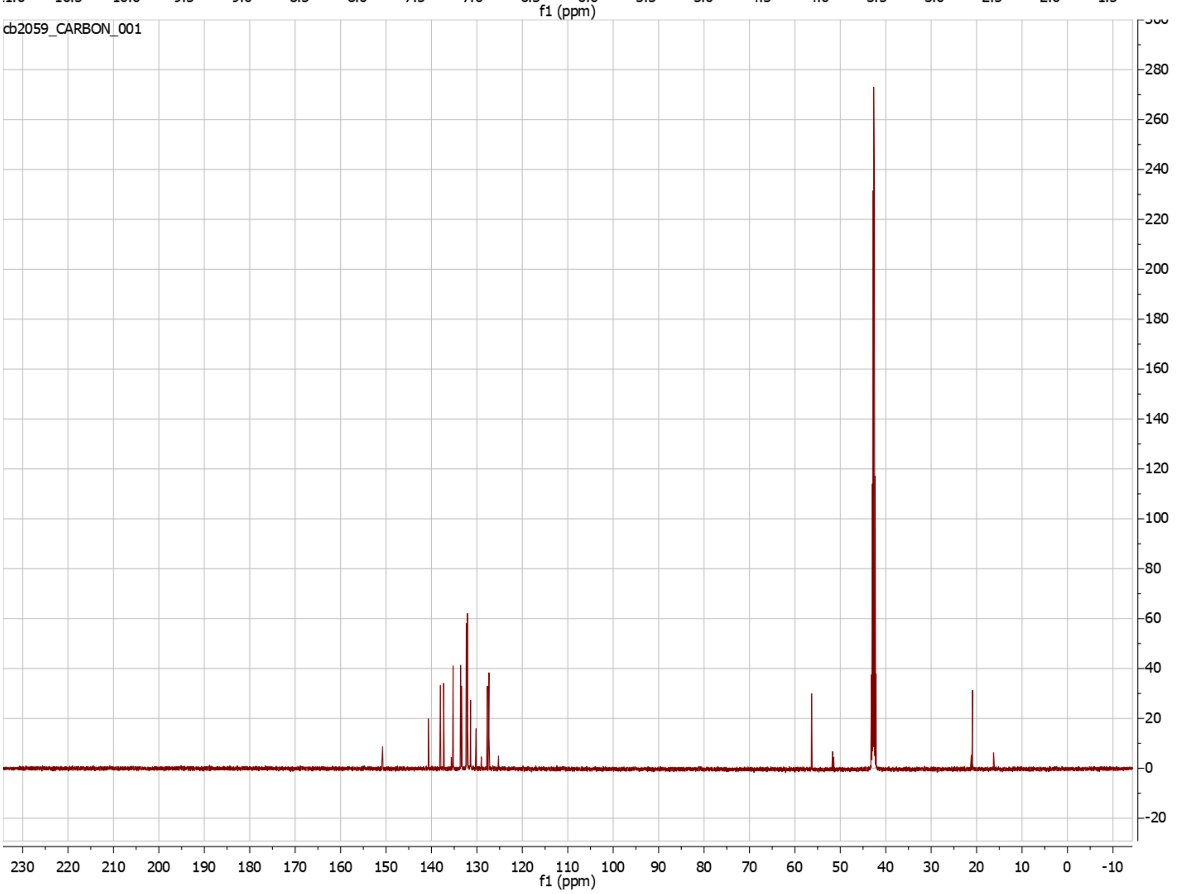

Spectrum compound 23

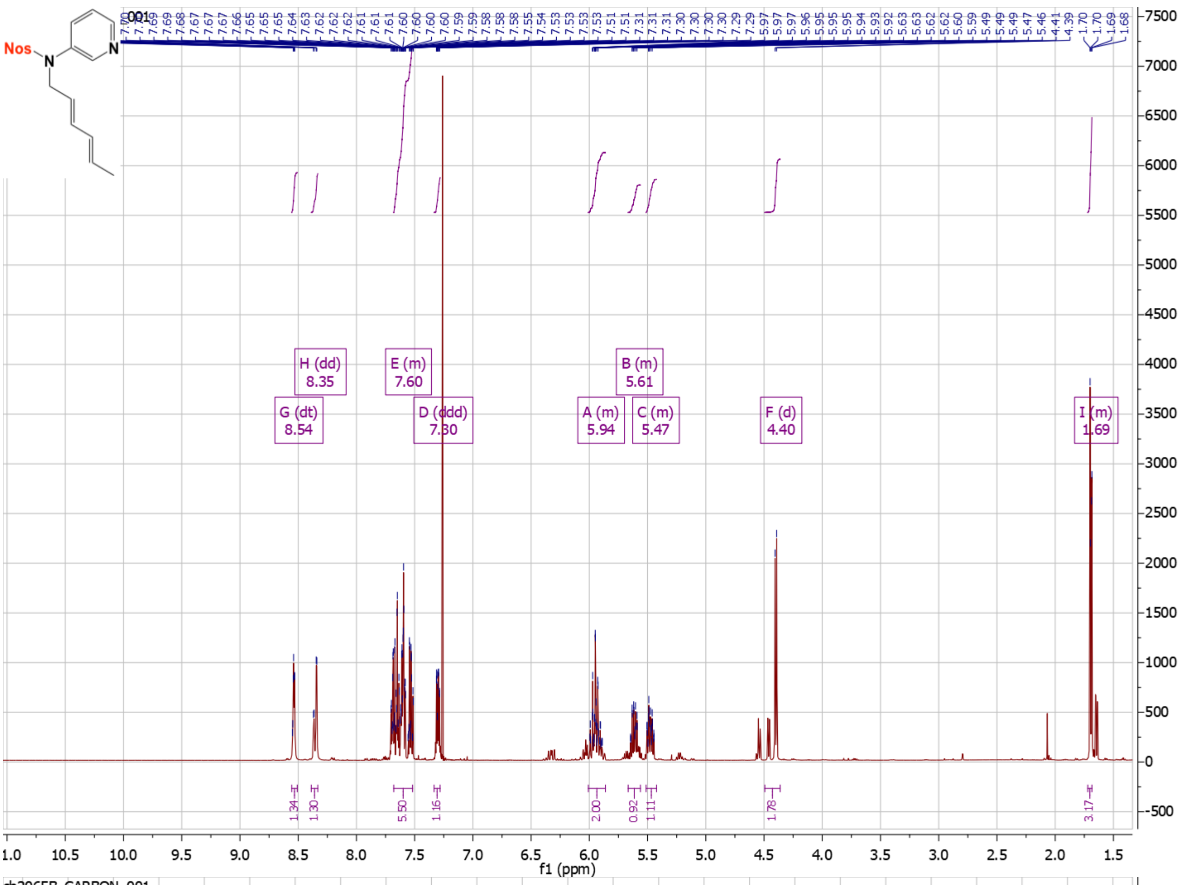

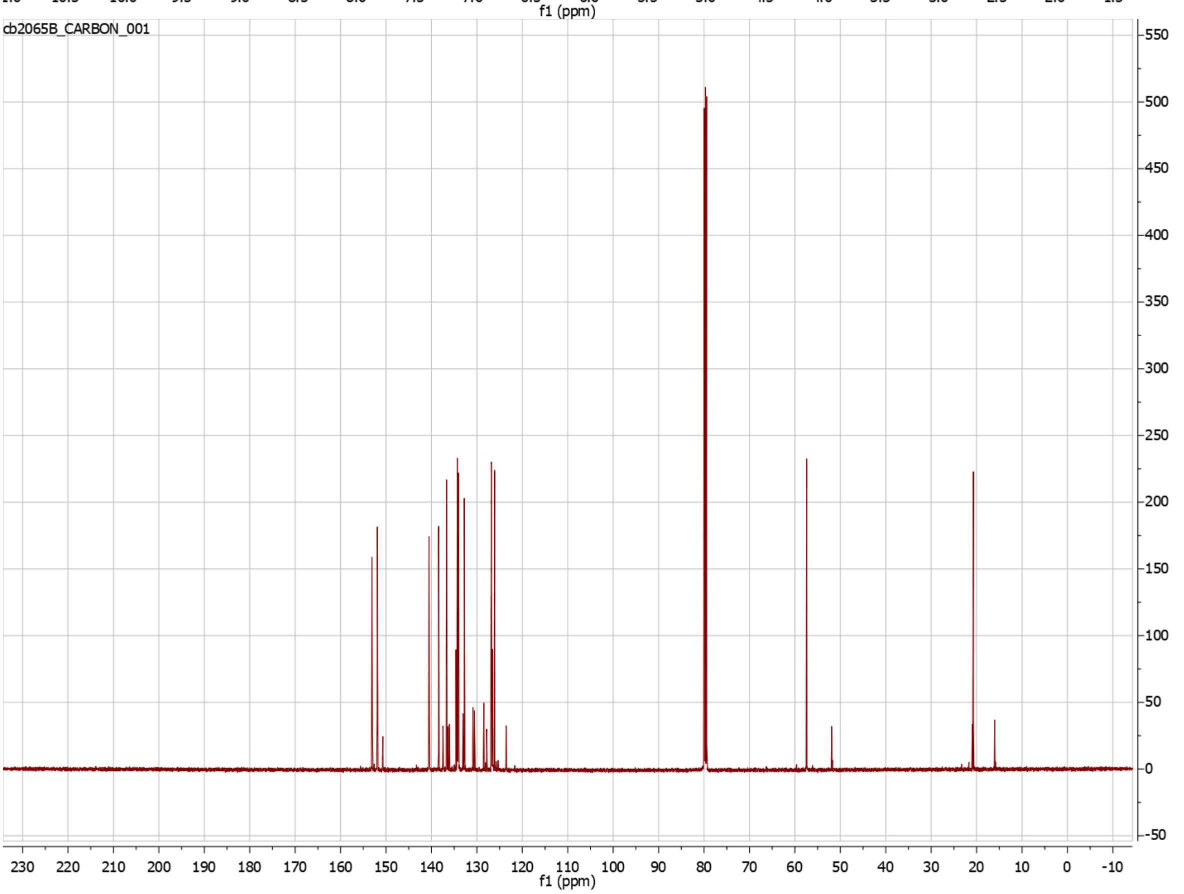

Spectrum compound 26

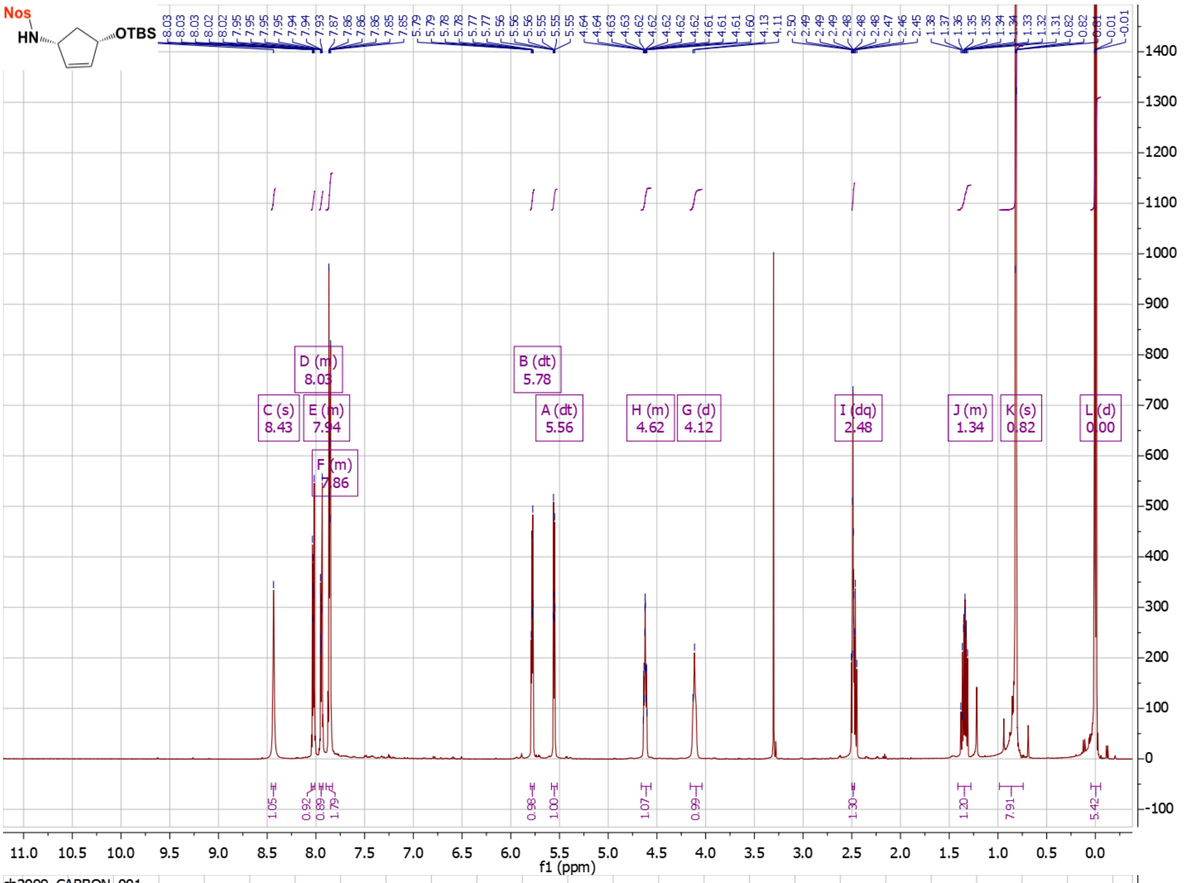

cb2099_CARBON_001

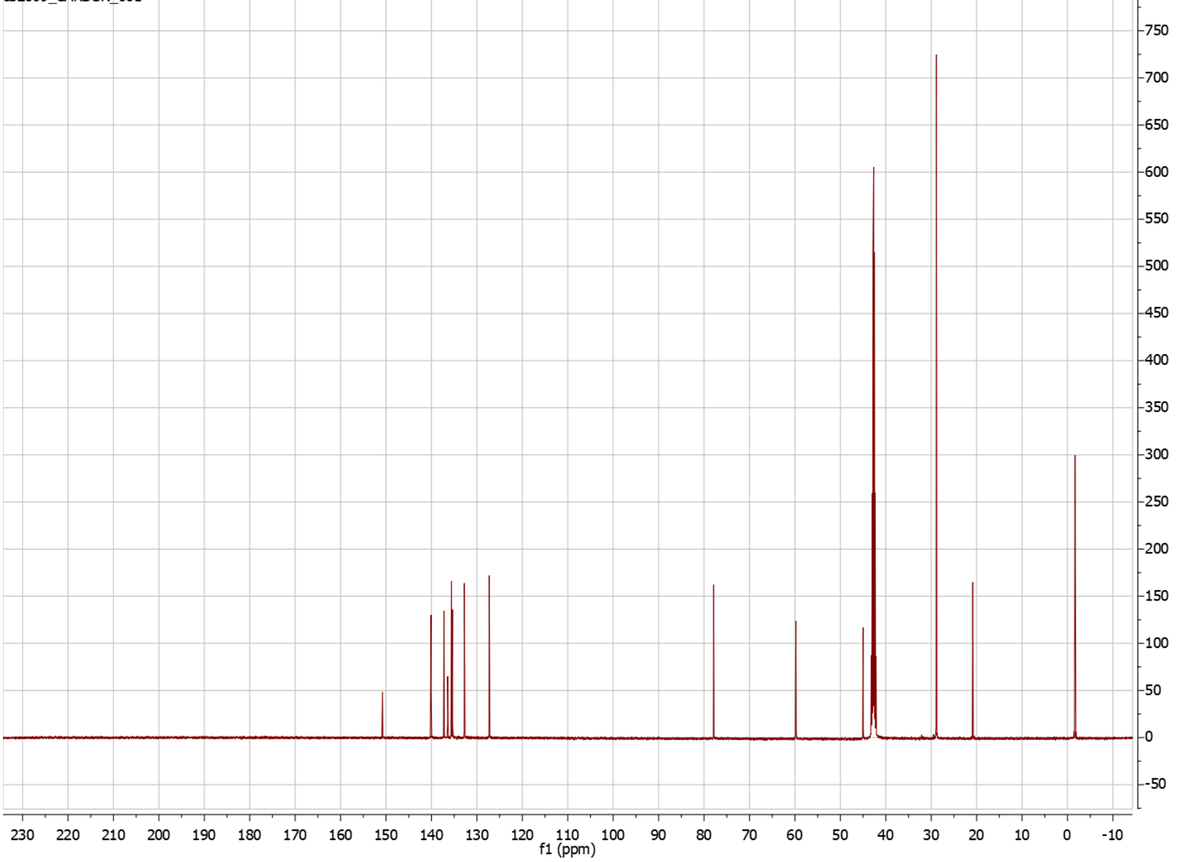

Spectrum compound 27

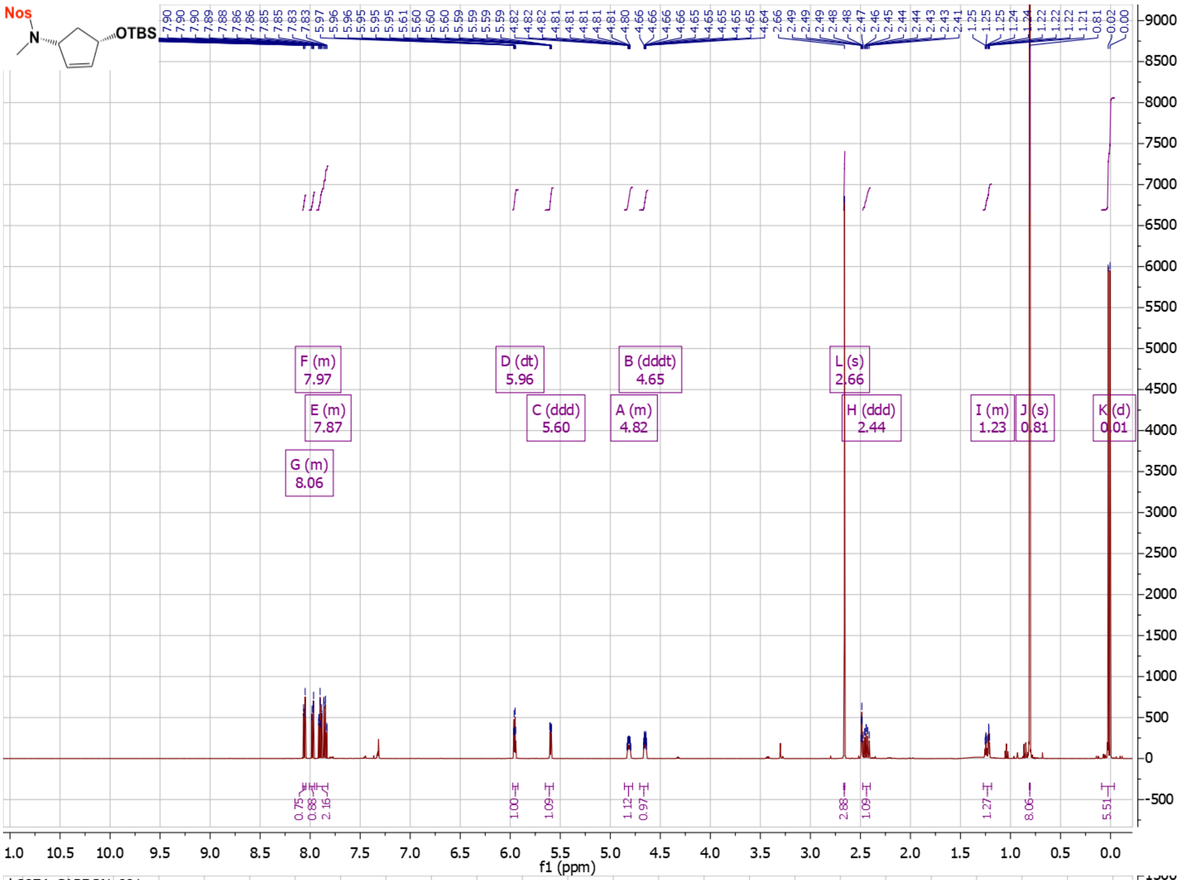

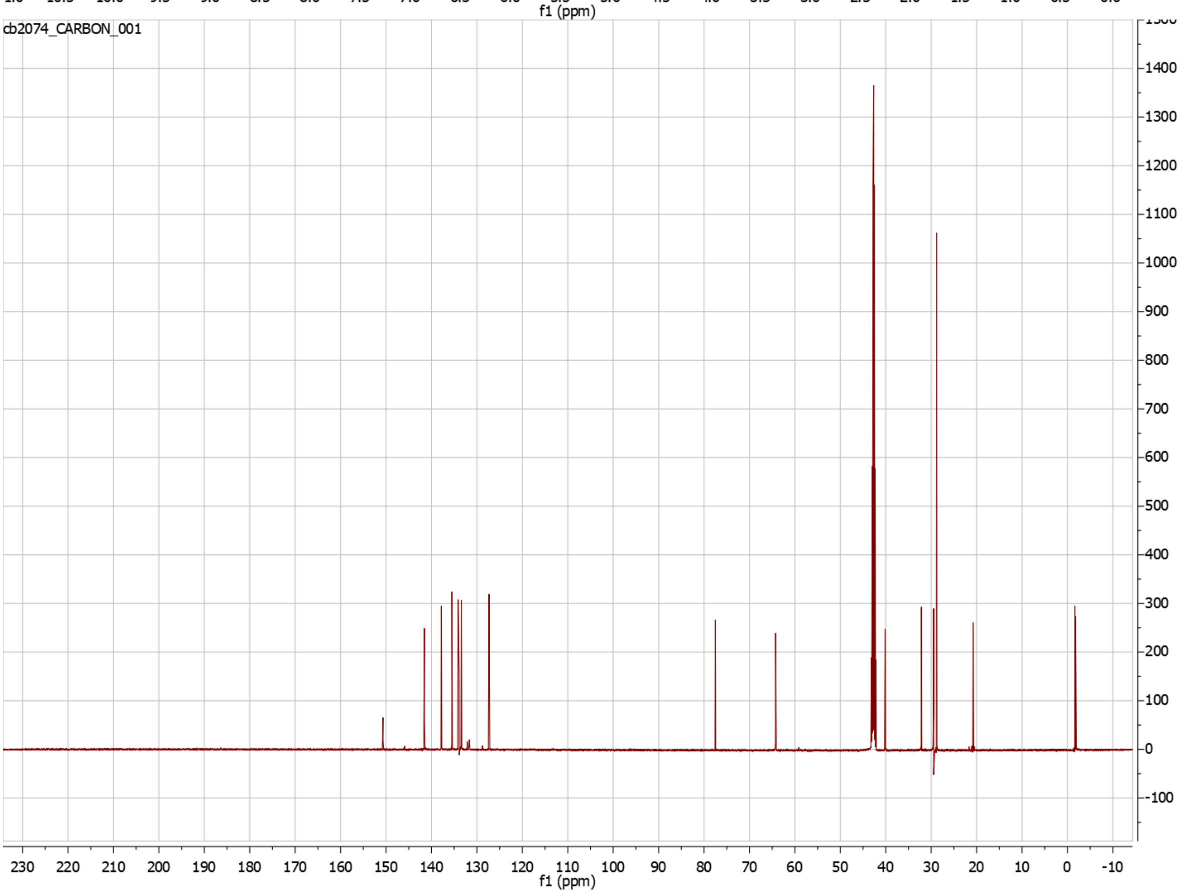

Spectrum compound 28

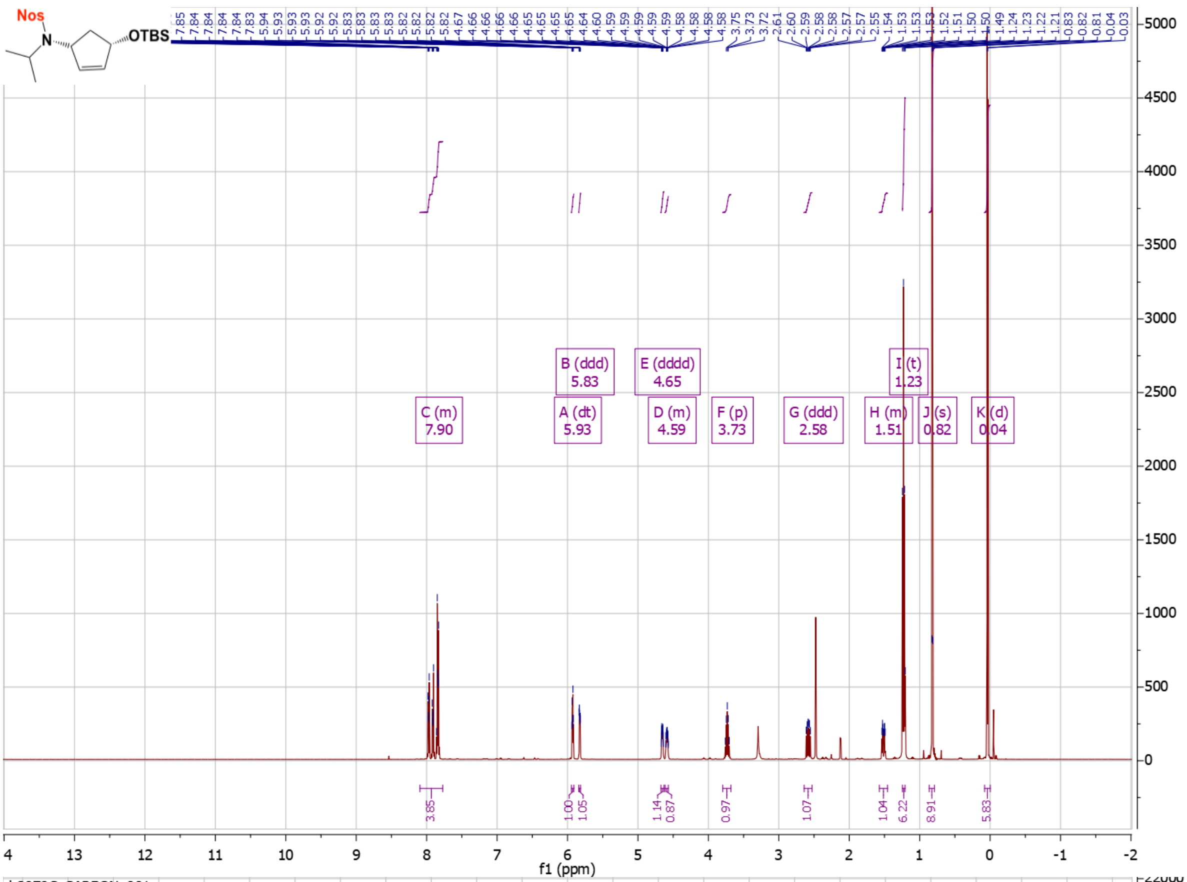

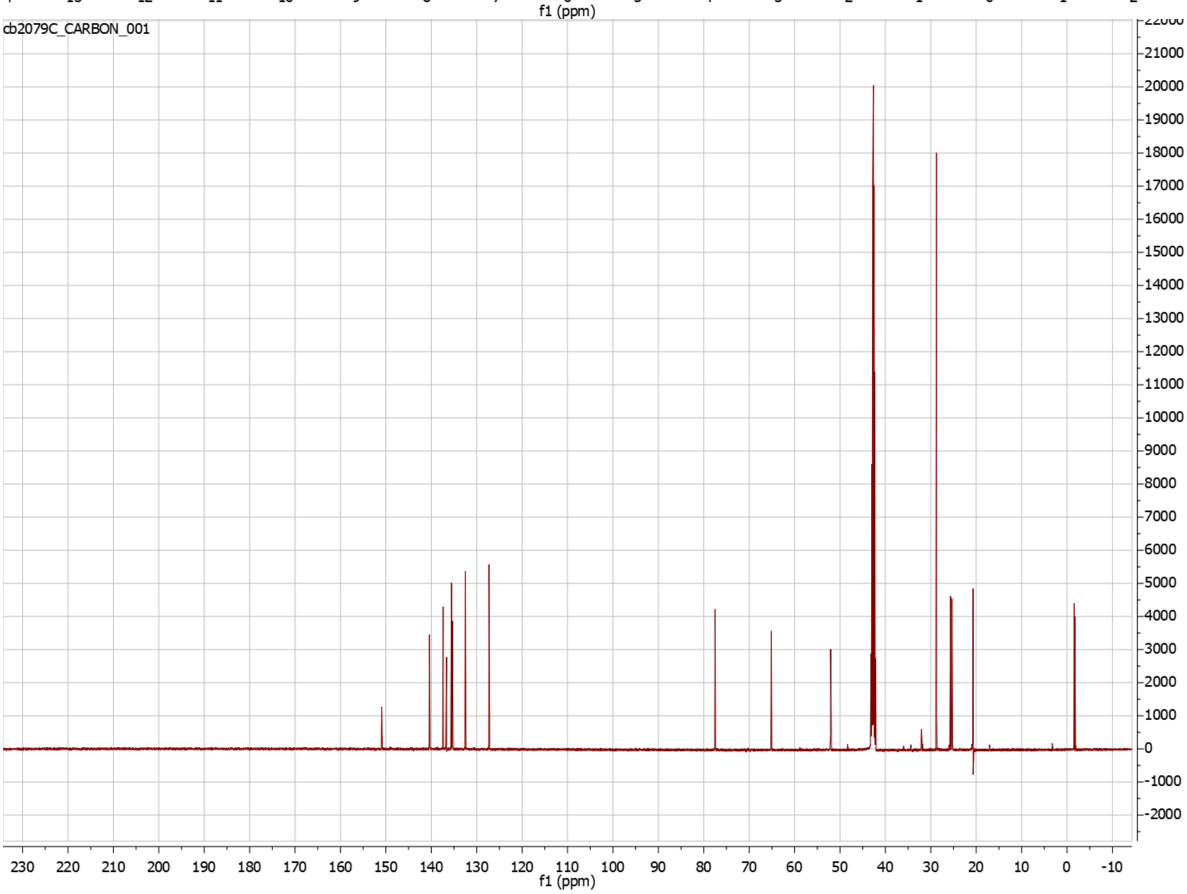

Spectrum compound 29

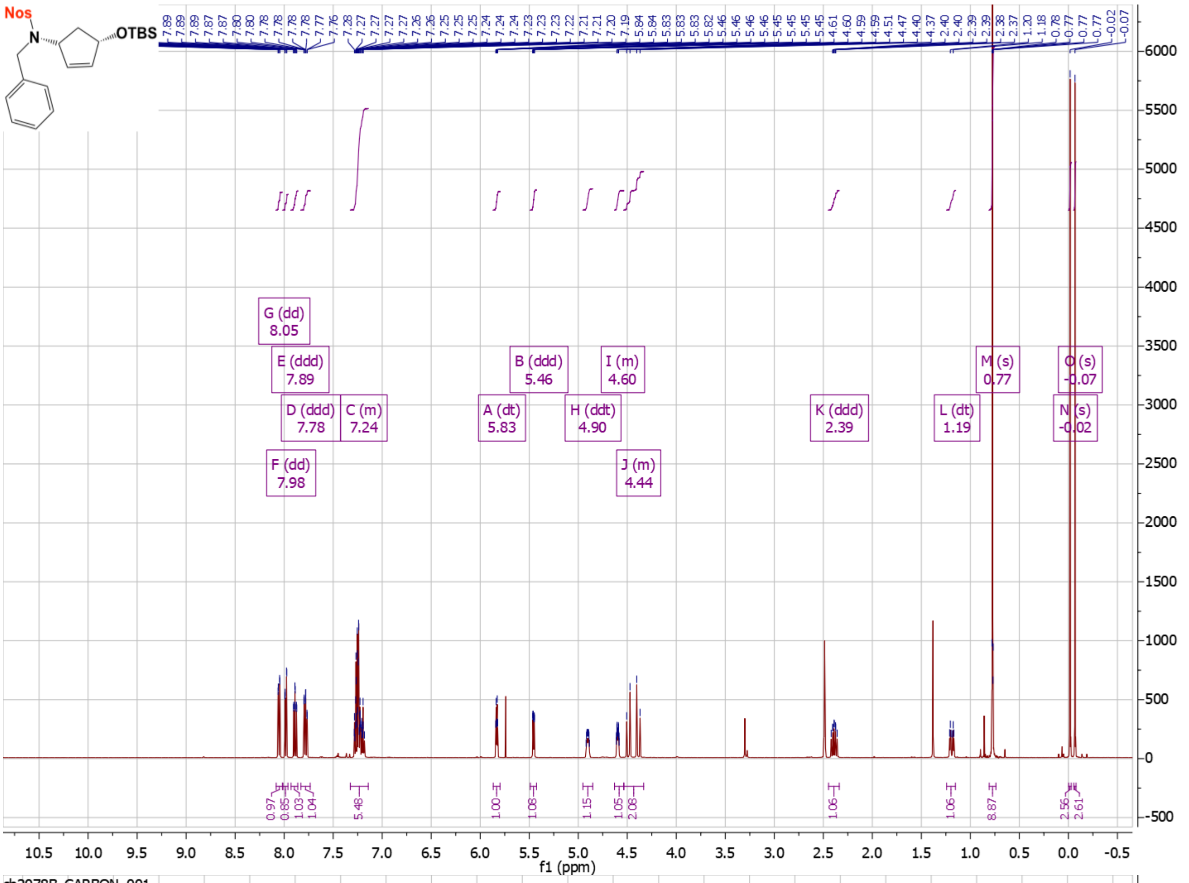

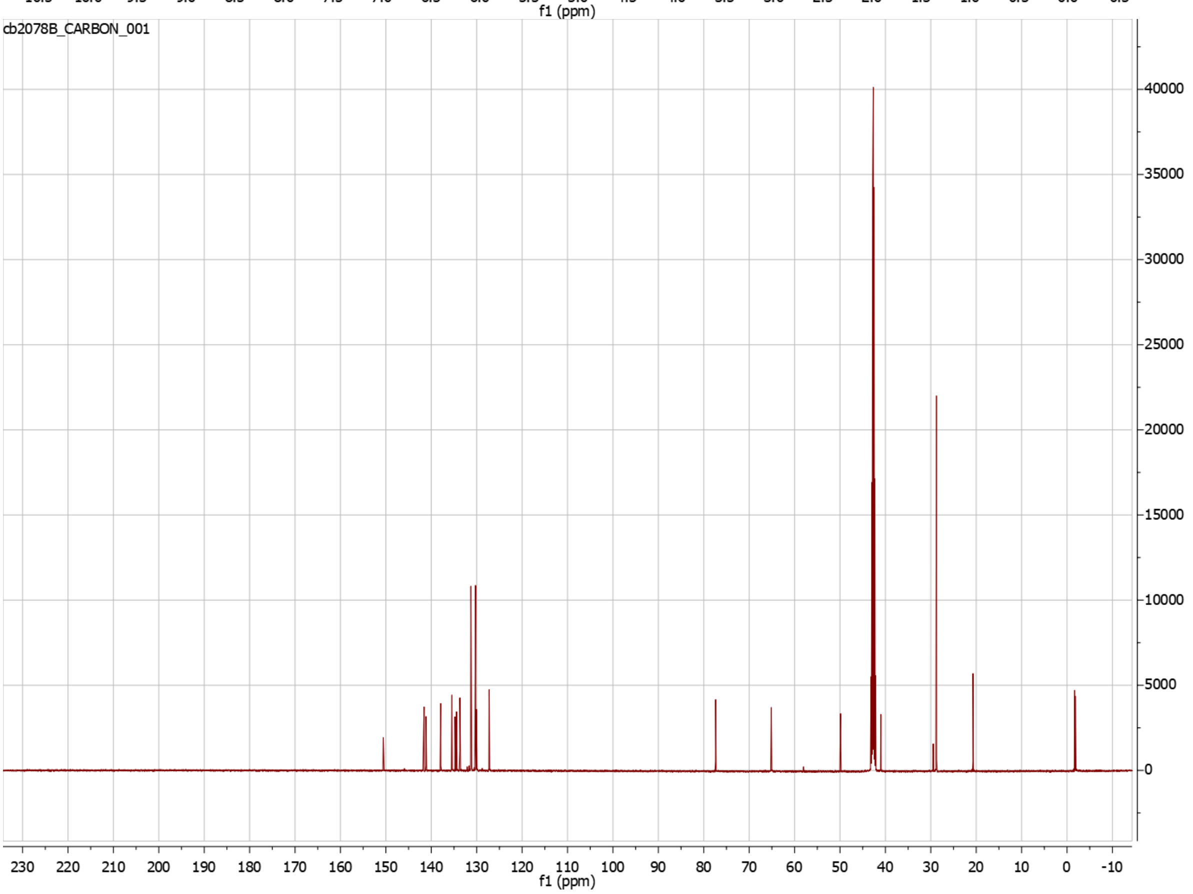

Spectrum compound 24

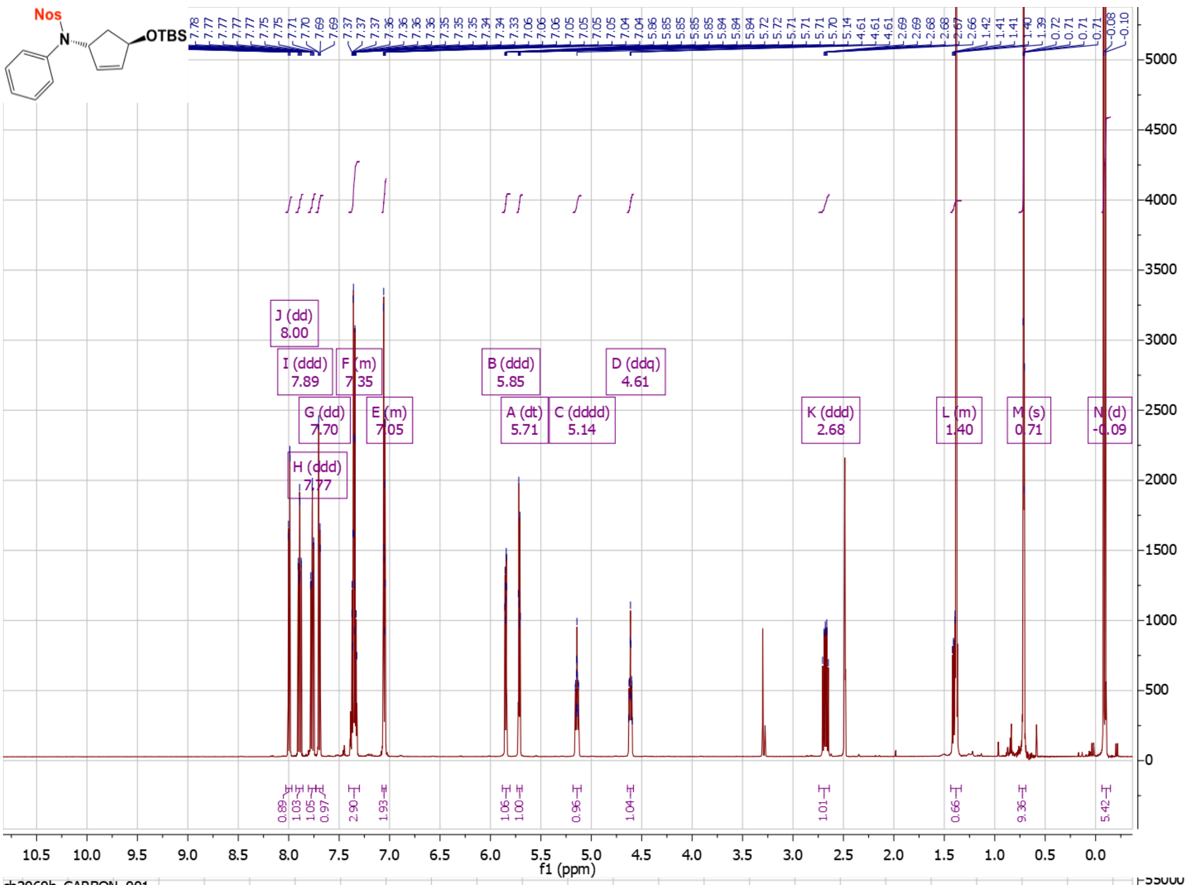

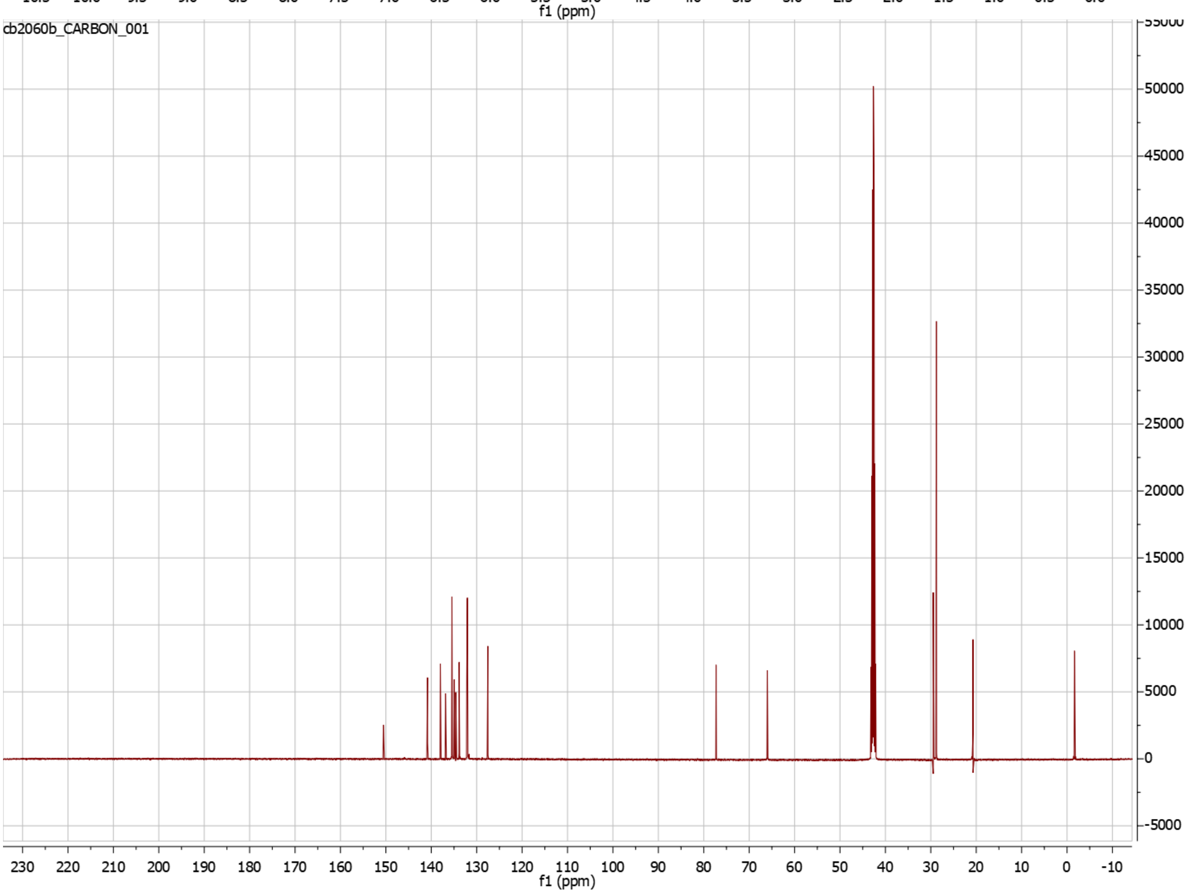

Spectrum compound 30

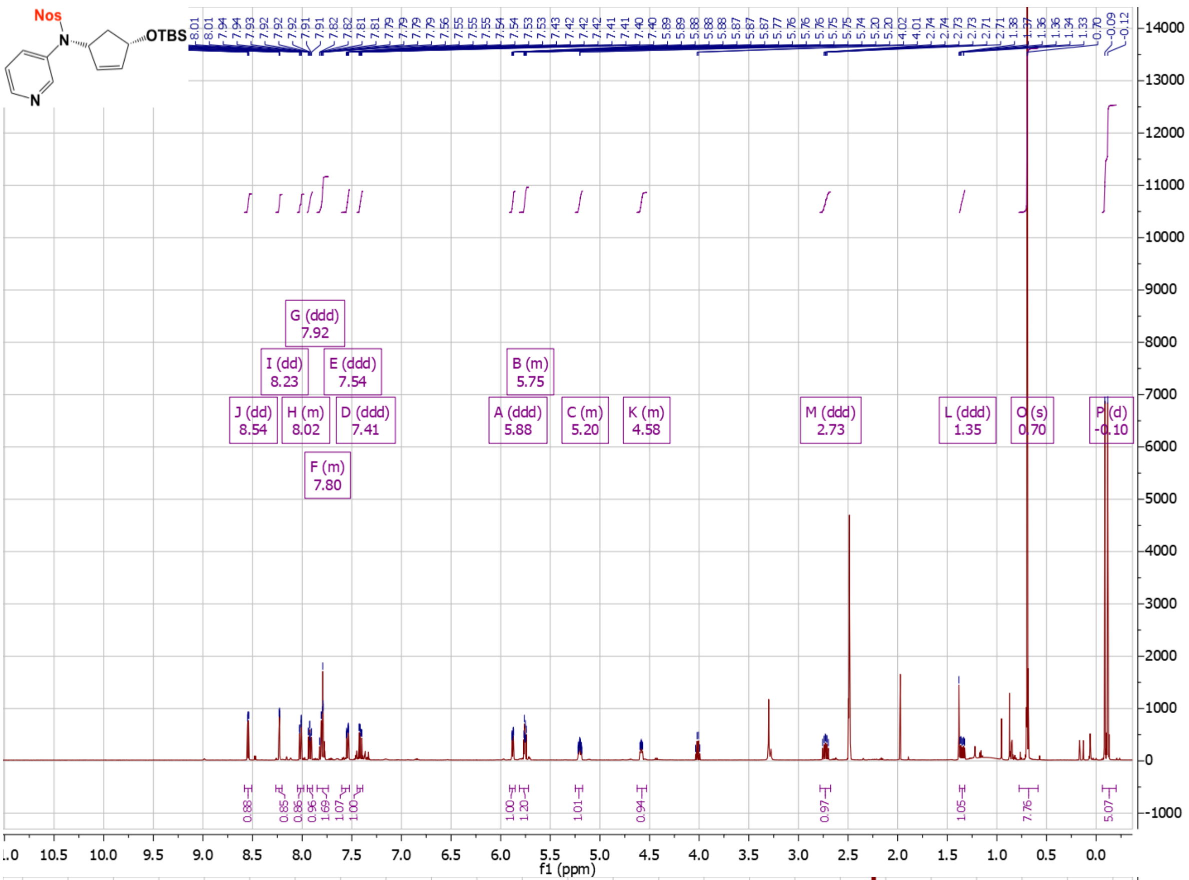

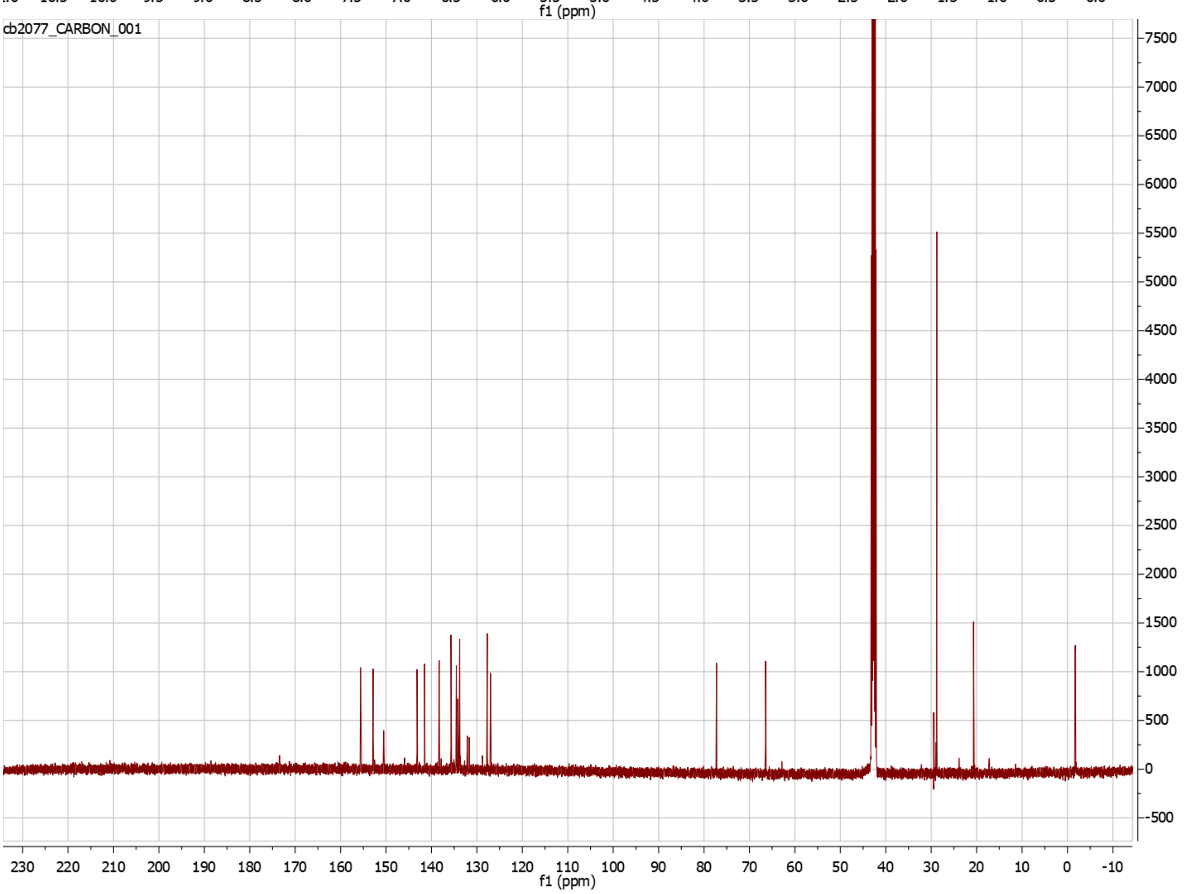

Supplement: Supplementary file 1 — Supporting Information [file OPEN-10-1166-s001.pdf]
